# Screening for adverse childhood experiences in pediatrics: A randomized trial of aggregate-level versus item-level response screening formats

**DOI:** 10.1371/journal.pone.0273491

**Published:** 2022-12-15

**Authors:** Dayna Long, Danielle Hessler, Kadiatou Koita, Monica Bucci, Mindy Benson, Rachel Gilgoff, Neeta Thakur, Nadine Burke Harris

**Affiliations:** 1 Center for Child and Community Health, Benioff Children’s Hospital Oakland, University of California, San Francisco, San Francisco, California, United States of America; 2 Department of Medicine and Family and Community Medicine, University of California, San Francisco, San Francisco, California, United States of America; 3 Center for Youth Wellness, San Francisco, California, United States of America; PhD, PLOS, UNITED KINGDOM

## Abstract

**Background:**

While there is growing support for screening for Adverse Childhood Experiences (ACEs), rigorous evidence on the efficacy and preference of screening methods is needed.

**Objective:**

To examine caregiver: (1) rates of disclosure of their child’s exposure to ACEs using item-level response (each item can be endorsed) versus aggregate-level response (only total score reported) screening format, (2) associations between family demographic factors and disclosure by screening format, and (3) emotional reaction and experience of screening formats in a diverse, low-income pediatric population.

**Methods:**

Caregiver participants (n = 367) were randomized to complete the Pediatric ACEs and Related Life Events Screener (PEARLS) tool, in an aggregate-level response vs item-level response format from 2016–2019. Select caregivers (n = 182) participated in debriefing interviews. T-test and chi-square analyses in 2019 compared PEARLS disclosure rates and reactions between the screening modalities. Regression models explored interactions with child characteristics. Thematic analysis of interview notes captured caregiver screening experience.

**Results:**

PEARLS disclosure rates were significantly higher in the aggregate-level response compared to the item-level response screening arm (p <0.05). This difference was accentuated for children identified as black and/or male (p <0.05). Caregiver reactions to PEARLS screening were rarely negative in either screening format. Qualitative data demonstrated strong caregiver preference for the item-level response format; additional themes include provider relationship, fear with disclosure, and screening outcome expectations.

**Conclusion:**

While caregivers reported a preference for the item-level response format, the aggregate-level response screening format elicited higher disclosures rates particularly for children who are black or ma.

**Trial registration:**

**Clinical trial registry:** NCT04182906.

## Introduction

Childhood adversity and trauma are common and consequential [1]. Adverse Childhood Experiences (ACEs), an important and specific set of childhood adversities, refer to ten categories of adversity (in 3 domains of abuse, neglect and household challenges) and are associated with poor health outcomes (i.e., chronic respiratory disease, heart disease, and cancer) in the landmark ACE Study conducted by the Centers for Disease Control and Prevention and Kaiser Permanente [2]. Preventing ACEs in the U.S. has the potential to reduce up to 1.9 million cases of heart disease, 2.5 million cases of overweight/obesity and 21 million cases of depression [1, 2]. A 2019 consensus report of the National Academies of Sciences, Engineering and Medicine recommended that providers “adopt and implement screening for trauma and adversities” [3] and the American Academy of Pediatrics (AAP) calls on pediatricians to “screen for the precipitants of toxic stress.” [4]. However, best-practices in screening methodologies for primary care practitioners are needed to ensure that providers can clinically incorporate screening into daily workflows and respond to positive screens. Prior to developing best practices, it is critical to understand patient and caregiver experiences of and preferences for screening, as well as factors that may facilitate disclosure of sensitive information. Extrapolating from screening for ACEs in adult patients and related interpersonal violence (IPV) literatures, patient acceptance of adversity screening has been noted to be high [5] with multiple factors associated with respondent comfort with disclosure, including: family demographic factors (e.g., race), provider relationship (e.g., trust, continuity of care), as well as privacy considerations including screening formats that allow for greater privacy (e.g., aggregated number of adversities vs. individual adversities) [6–8]. In pediatrics research is still at the nascent stages of our understanding of considerations and implementation of adversity and trauma screening in primary care workflows. Only one previous study has examined screening format, where the authors reported greater disclosure of child adversities when privacy is provided through an aggregate over single item response format [9] and little is known around additional factors contributing to disclosure or screening format preference. As prospective screening for childhood adversity and trauma expands, there remains a great need to better understand respondents’ experience of screening as well as factors that influence their disclosure of adversity and trauma. The Pediatric ACEs and Resiliency Study is a randomized controlled trial on early childhood adversity and trauma in a pediatric primary care clinic serving a mostly low income community. The current report from the Pediatric ACEs and Resiliency Study aimed to 1) compare disclosure rates on the Pediatric ACEs and other Related Life Events Screener (PEARLS) tool in an item-level response format (in which each item is endorsed yes/no) versus an aggregate-level response screening format (in which a total numeric count is reported); 2) examine associations between participant demographic factors (e.g., gender, race) and the PEARLS tool by screening format, and 3) to understand caregiver preferences and experience (including emotional reaction) to completing the aggregate-level response and item-level response screening methods of the PEARLS tool [10]. Distinct from studies focused on the validation of screener tools or intervention efficacy, the aim of the current paper is to describe the challenges and opportunities with varying formats of screening for childhood adversity and trauma in the pediatric primary care setting.

## Methods

### Study population and design

**Fig 1:**
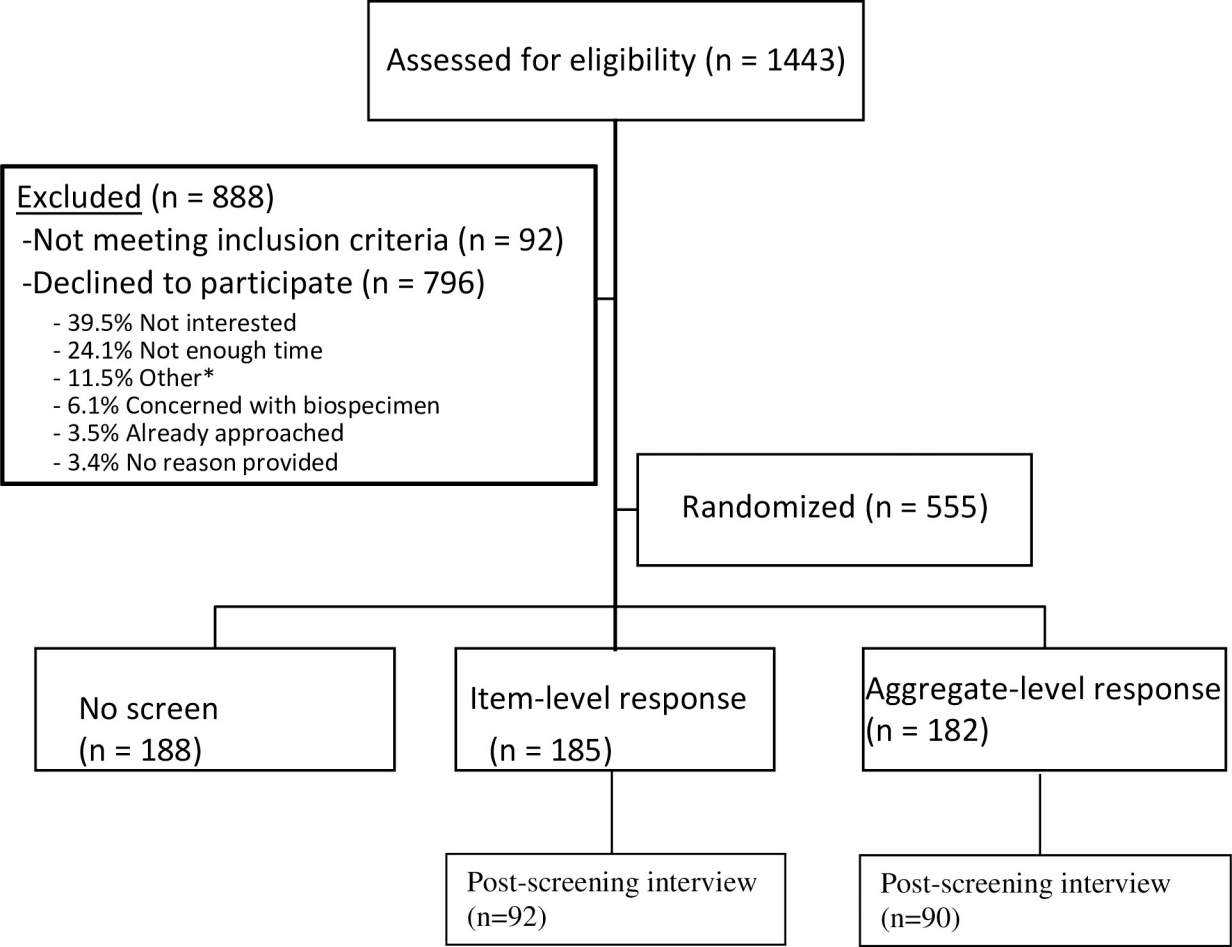
Consort diagram of randomization by screening format. *Other includes not primary caregiver (n = 27), did not want to consent without spouse (n = 20), sibling in study (n = 17), child declined (n = 16), transportation (n = 13), more time to think about study (n = 12) moving from area/ traveling (n = 6), not feeling well (n = 3).

The PEdiatric ACEs and ResiLiency Study is a 12-month randomized control study (NCT04182906) designed to 1) validate a prospective pediatric screen for ACEs and related life events (the PEARLS tool), 2) examine the association between stress-related biomarkers and adversities identified with PEARLS, and 3) pilot interventions to prevent and mitigate the toxic stress response in pediatric settings. Participation in the larger study included four study visits for survey completion (time 1–4), biomarker collection (time 2–4), and dependent upon PEARLS screening score and randomization, participation in a social or psychosocial intervention (between time 2–3). Of 1443 individuals approached to participate, 888 were excluded (796 declined participation with most common reasons included lack of time and interest, 92 were ineligible). Eligible caregivers (n = 555) were enrolled and randomized via a random number generator (randomization blocks of 12) and programmed by the study analyst to automatically display to the research coordinator via RedCap to one of the three screening formats in a 1:1:1 allocation ratio (n = 188 no screening, n = 185 Item-level response screening, and n = 182 Aggregate-level response screening) (Fig 1). The present study focuses on caregivers randomized to the Item-level or Aggregate-level screening formats, resulting in a study sample of n = 367. Child participants were eligible if they were between the ages of 3 months to 11 years, and caregivers were eligible if they were aged 18 years or older, the primary caregiver, and speak English and/or Spanish, and receiving primary care at UCSF Benioff Children’s Hospital Oakland (BCHO), a large, urban Federally Qualified Health Center. Siblings were excluded from participation. All participants provided written informed consent and the study was approved by UCSF BCHO Institutional Review Board. The authors confirm that all ongoing and related trials for this drug/intervention are registered with clinicaltrials.gov. Registration approval occurred after the date of the first participant enrollment due to a research team administrative error. Data was collected from 2016–2019 and analyzed in 2019. Participants were compensated a total of $300 for participating in the entire 12-month period. Trained, bilingual research staff randomized participants via an automated randomization table (RedCap assigned PEARLS tool condition), administered the PEARLS tool, followed by additional baseline psychological and health questionnaires. Research staff were not blind to PEARLS tool condition following randomization given the nature of the interaction with participants around their screening (where applicable). A subset of caregiver participants then additionally completed a questionnaire about their emotional experience and/or a brief interview about their experience completing the PEARLS tool (see Cognitive Interview section below for details). Finally, the caregivers in the Total ACEs Score screening group were asked to specify the items they previously endorsed to collect individual item level endorsement for this group. Caregivers in both screening formats received anticipatory guidance from and had the opportunity to discuss endorsed adversities with their provider, as well as access to interventions and resources.

### Assessment of adverse childhood experiences and related life events

ACEs and Related Life Events were measured using the PEARLS tool, an adversity screening tool developed with input from multiple staff and patient stakeholders [10]. The PEARLS tool includes the ten original ACE categories [2] and assessment of seven additional Related Life Events [10] thought to increase the risk of a toxic stress response [11] including separation from caregiver, caregiver death and caregiver physical illness as well as social determinants of health including food insecurity [12–15], housing instability [16–18], discrimination [19–26], and community violence (including police violence and bullying) [27]. Participants were randomized to: (1) no screening (control group), (2) screening with item-level response format of PEARLS tool (responses to each adversity were recorded as yes/no) or (3) screening with aggregate-level response format of PEARLS tool (responses were recorded as a total count, i.e., “How many of the following has your child experiences?’).

### Post-screening cognitive interviews

Two rounds of cognitive interviews were conducted that combined included a sub-sample of caregiver participants (n = 182) participated in a short 5–7 minute post-screening interview regarding their screening experience. In the first round of interviews, caregivers (n = 73; n = 35 aggregate-level response and n = 38 Item-level response) were initially asked about their screening experience in the same format they completed the screening. All caregiver participants were invited to participate in the post-screening interview until saturation was reached. After a three-month break to further review responses, a second set of caregiver participants (n = 109; n = 55 aggregate-level response and n = 54 item-level response) reported on the screening format they were not assigned (i.e., participants receiving item-level response format were asked about how they would feel about completing an aggregate-level response screen and vice-versa.). As in the first round, all caregiver participants during this period of study enrollment were invited to participate in the post-screening interview until saturation was obtained in the second round with these alternative questions and no new information was generated. Interview questions included: “What do you think of taking the PEARLS questionnaire for you child knowing your health care provider will see (how you answered each question/your total adversity score)?” (item-response/aggregated response) Do you feel that information is helpful to your health care provider? Do you feel that your provider should know more or less information? The interviewer took field notes during the discussions, and used quotation marks whenever exact statements were noted.

### Caregiver emotional response to screening

The Positive and Negative Affect Scale (PANAS) [28] was additionally completed by caregiver participants who participated in second round of interviews (n = 109; n = 55 de-identified and n = 54 identified) prior to the interview questions. The PANAS includes 10 positive (e.g., interested, excited) and 10 negative valence items (e.g., scared, ashamed) on a Likert scale, from 1 = very little/not at all to 5 = extremely, and assessed participants’ emotional reaction to the screening.

### Analysis

#### Quantitative

Descriptive statistics (frequencies, measures of central tendency) of ACEs, PANAS emotional responses, and mandated reporting cases were calculated. The original ten ACES were examined both as a total count (0–10) as well as in thresholds suggested by the literature (0, 1–3 ACEs, and 4 or more ACEs). Differences in adversity disclosure in the item-level response and aggregate-level response screening arms were evaluated with Student t-test, paired t-tests, and chi-square or McNemar analyses, as appropriate. Associations between participant demographic characteristics and reported count of adversities were examined with binomial regression models. Associations between adversities and PANAS emotional responses were evaluated using Spearman’s rho. For continuous measures or a 15% absolute difference (for dichotomous measures; e.g., % reporting ≥ 4 ACEs) endorsing based measures of adversity, which the authors identified as a meaningful difference based on clinical experience and existing literature [9]. Analysis was performed with SPSS version 26.

#### Qualitative

Qualitative notes were uploaded and coded within the qualitative program Dedoose [29], A theoretical thematic analysis, with open coding, using the Braun and Clarke six step inductive analysis technique [30] was used to identify and create codes, with related codes reviewed and organized into themes. Twenty percent of interviews were coded in common across two coders (independent of interviewer). Coding discrepancies were settled by consensus and inter-rater reliability (IRR) was 92%.

## Results

A description of the sample appears in Table 1. There were no differences in baseline demographics by screening arm, nor in the subsample of the Aggregate-level response arm that identified ACEs experienced post-screening (n = 155) or the cognitive interview procedures (n = 182).

**Table 1:** Child and caregiver characteristics by PEARLS screening format.

	Item-level Response PEARLS Screen (n = 185)	Aggregate-level Response PEARLS Screen (n = 182)	Cognitive Interview (n = 180)
	Mean (SD) or %	Mean (SD) or %	Mean (SD) or %
Child age	5.91 (3.57)	5.83 (3.46)	5.79 (3.53)
Child gender (female)	45.95%	46.15%	45.90%
Caregiver relationship (mother)	81.62%	80.21%	78.45%
Highest school (≤ high school)	30.04%	34.80%	30.7%
Household annual income (≤ 25K)	43.1%	39.90%	41.60%
Years coming to clinic (≥ 1 year)	84.30%	84.20%	83.60%
Child race—non-Hispanic White	4.32%	3.85%	4.42%
Child race—Hispanic White	16.76%	19.78%	19.88%
Child race–non-Hispanic Black	51.89%	59.34%	53.59%
Caregiver age	36.89 (10.08)	36.54 (11.23)	36.82 (10.93)
Caregiver gender (female)	90.16%	90.05%	90.05%
Caregiver race—non-Hispanic White	7.57%	8.24%	9.94%
Caregiver race—Hispanic White	10.27%	15.38%	16.02%
Caregiver race–non-Hispanic Black	55.67%	58.24%	53.03%

### ACEs disclosures by screening format

Report of adversities were common, regardless of screening format: 74–79% of families reported 1 or more adversities using the PEARLS tool (Table 2). Mean reported adversities were greater in the aggregate-level response compared to the item-level response screening format for the Ten Original ACEs and PEARLS (i.e., Ten Original ACEs plus seven Related Life Events) p< 0.05; d = .25 for both) and marginally greater for Related Life Events (p = 0.08; d = .18). No differences by screening format were noted when examining the cut-point of four or more ACEs (Table 2). Post-screening identification of adversities within the aggregate-level response screening group was obtained for 83.7% (n = 155/182) of caregivers with missing data largely attributed to participant time constraints. Disclosure of adversities in the aggregate-level screening group compared to post-screening identification did not reach statistical significance (Table 2). Among participants in the aggregate-level response screening condition, there were no statistically significant differences in disclosure of adversities when reporting under the aggregate-level vs item-level response format.

**Table 2:** Disclosure of adversities by PEARLS screening group.

	A: Item-level response (n = 185)	B: Aggregate-level response (n = 182)	C: Post-screening Item level response (within aggregate-level response) (n = 155)	p value Item-level vs. Aggregate-level (A vs. B)	p value Item-level vs. post-screen item level (A vs. C)	p value Aggregate-level vs. post screening item level (B vs. C)
Primary analysis						
Total PEARLS Score (Original ACEs + Related Life Events) (mean, sd)	2.84 (3.03)	3.63 (3.28)	3.44 (3.35)	0.02	0.09	0.40
Exploratory and Sub-analyses						
Original ACEs score (mean, sd)	1.78 (2.04)	2.32 (2.21)	2.17 (2.18)	0.02	0.09	0.13
Related Life Events score (mean, sd)	1.06 (1.36)	1.31 (1.38)	1.26 (1.46)	0.08	0.18	0.75
0 ACEs (%) Original ACEs	35.1% (65)	28.0% (51)	32.3% (50)	0.14	0.58	0.50
0 Related Life Events (%)	47.0% (87)	36.8% (67)	41.9% (65)	0.05	0.35	0.51
0 ACEs (%) Total PEARLS score	26.5% (49)	21.4% (39)	25.8% (40)	0.26	0.89	0.99
≥4 Original ACEs (%)	18.9% (35)	25.2% (46)	23.2% (36)	0.14	0.33	0.58

Note: Mean (SD) or frequency percentage (n) are presented. Comparisons of items and aggregate level (A vs. B_ and item vs. post-screen item (A to C) were tested with t-tests and chi-square analyses. Comparisons of the two versions of the aggregate-level screening (within subjects; n = 155 for comparison) were additionally tested with paired t-tests and McNemar tests.

**Fig 2:**
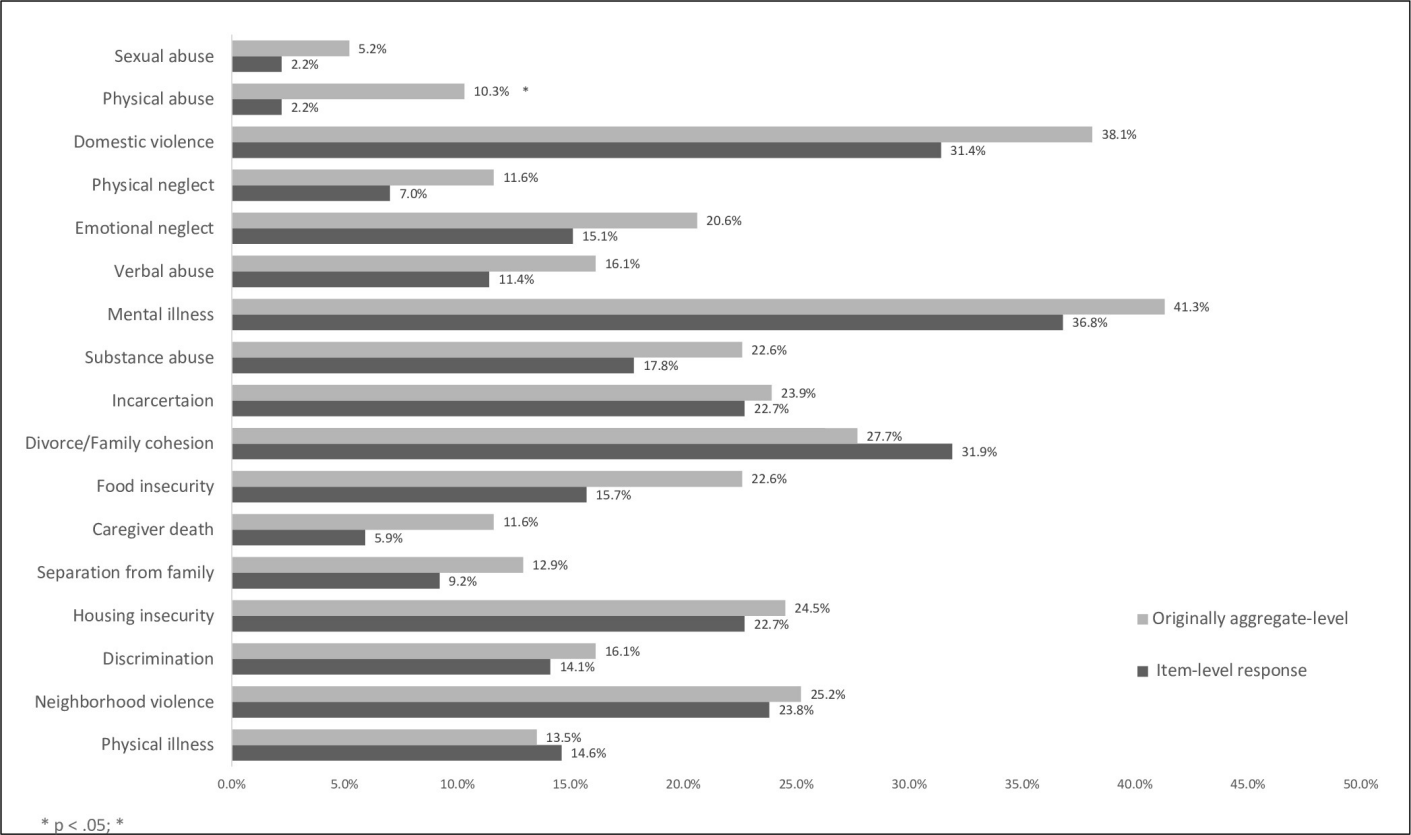
Disclosure of individual item adversities by initial screening format.

Rates of endorsement of specific adversity items also differed by screening arm with higher rates of disclosure of physical abuse (p = .001; d = .28) in the aggregate-level response screening arm (Fig 2). Of the 367 families screened, 54 possible cases were documented for physical and sexual abuse and neglect. Each family met with the provider and/or mental health clinician and safety was assessed. Four Child Protective Service Reports were generated, three of these cases had been previously reported, and one new case was made.

### Associations between ACEs and family demographics by screening format

Exploring whether differences in disclosure rates by screening modality differed by participant demographics, a significant interaction for child gender (p<0.01 Ten Original ACEs, p<0.01 Related Life Events, and p<0.001 PEARLS tool) was found. While the number of the total PEARLS tool adversities was nearly identical under the two screening formats for female children (mean±sd = 3.11±3.36 and 3.08±3.09 item-level response and aggregate-level response respectively), among male children the total number of reported adversities was considerably higher in the aggregate-level response vs item-level response format (4.09±3.39 vs. 2.61±2.75). Findings for the Ten Original ACEs were similar (girls mean±sd 1.96±2.11 and 1.92±2.22; boys 2.62±2.25 and 1.66±1.87 for aggregate-level response and item-level response formats respectively). Results for Related Life Events demonstrated similar gender differences by format.

Similarly, there was a significant interaction for race for the Ten Original ACEs and PEARLS scores (both p<0.05). Among children identified as non-Hispanic black, mean disclosure was greater in the aggregate-level response than the item-level response screening format (2.51±2.27 vs. 1.85±2.10 for the Ten Original ACES and 3.94±3.34 vs. 2.97±3.23 for PEARLS). Differences by screening format were present but less prominent for children identified as Latino for PEARLS score (3.69±3.33 vs. 3.10±2.61 for Aggregate-level response versus Item-level response format respectively) and did not differ for Ten Original ACEs. No differences were seen for other racial/ethnic groups or for Related Life Events.

### Caregiver preference and experience of PEARLS screening by screening format

#### Caregiver emotional response

To screening assessed through PANAS scores (n = 109; 1–5 scale) noted overall moderate positive affect (mean±sd) in the item-level response (3.75±0.97) and aggregate-level response groups (3.56±0.90) and infrequent reports of negative affect (1.37±0.53 and 1.34±0.45) for the item-level response and aggregate-level response groups respectively, with no significant differences between screening formats. Number of reported adversities was not associated with emotional response in aggregate-level response screening; while caregivers reporting more adversities on the item-level response screen reported greater positive (Spearman’s rho = 0.25, 0.38 and 0.32; all p<0.05) and negative affect (Spearman’s rho = 0.49, 0.32 and 0.49; all p<0.05) for the Ten Original ACEs, Related Life Events, and PEARLS score respectively. Average negative affect (mean±SE) among caregivers was 1.05±0.02 for those reporting no ACEs, 1.48±0.10 for 1–3 ACEs and 1.61±0.20 for 4 or more ACEs; while positive affect was 3.30±0.33, 3.88±0.13 and 4.13±0.26 for the same ascending ACEs categories.

#### Caregiver screening preference

Screening preference by format and interview round appear in S2 Table. Overall, seventy percent (n = 110/182) of interviewees stated a preference for the item-level response format, while 12% (n = 19/182) expressed a preference for the aggregate-level response format. Among families with a preference for the aggregate-level response, 95% ha reported a total PEARLS score of ≥1 (range 1–12).

*Caregiver experience of the PEARLS tool*. Three themes (including 11 codes; S1 Table) centered around: (1) expectations for the screening outcome (including screening outcome expectancy and the screening serving as an “icebreaker”), (2) quality of provider relationship (including level of familiarity vs. unfamiliarity, duration and quality of relationship and comfort with provider), and (3) caregiver personality and emotional state (including personal attributes of the caregiver, fear, and adversity exposure status) were identified and examined by screening preference to understand the themes in the context of both PEARLS tool formats.

*Expectations for the screening outcomes*. When responding about their feeling and preference for The screening format (item-level response versus aggregate-level response), participants frequently referred to the screening outcome and their perceived benefit from their provider knowing the details of each item as the rationale for their choice. Expectations for screening outcome was mentioned by 89% of those who stated preference for item-level response screening; expressing a concern that they would not get the support they needed if the provider did not know their specific responses: “*Best for the doctor to know in case of any resources she might be able to recommend*. *If she doesn’t know what’s going on*, *then we can’t get help* (female, 29yrs).” Similarly, the majority of participants who stated a preference for item-level response screening expressed that completing the screener prompted a conversation they would not have brought up themselves with their provider. Participants whose stated their preference was the aggregate-level response format acknowledged that the item-level response screening format would more likely result in directed support or better care than the aggregate-level response format: *“I liked it anonymous*, *but in this case*, *having it known wa/s more beneficial*, *and I knew that* (female, 25yrs).”

*Quality of relationship with the provider*. Respondents frequently referred to their strong relationship with their provider as a reason for sharing adversities. Many, especially those reporting a preference for the item-level response screening format, stated they had seen the same provider for a long time, he/she was familiar with their lives, and they were comfortable with disclosing adversities because of the trusting relationship. One participant (female, 29 years) noted, “*If there was something serious happening with us*, *we’d tell her*!” The quality of relationship with the provider was also cited by participants whose stated preference was the aggregate-level response format. They stated aggregate-level response screening format was fine as their provider already knew about their adversities, or that they would feel more comfortable directly discussing their adversities with the provider.

*Caregiver personality and emotional state*. The third theme that emerged was the participants’ personality and emotional response to their experience with adversity. Participants self-described their personal style, ranging from being private to “I’m an open book.” Participants who preferred the item-level response format were more likely to describe themselves as open; whereas, those who preferred the aggregate-level response format described themselves as private, or not easily trusting. Several participants with a preference for aggregate-level response format, expressed fear of being judged, embarrassed, and afraid about who might see their answers. Participants connected fear to race/ethnicity. “*Race and culture are a part of this*, *too*. *and immigration status*. *You don’t really know who is collecting this information…(Male 43yrs)*.*”* “*A lot of people of color fear being questioned about their parenting practices (male*, *35yrs);” “As a Black man*, *I have to ask myself*, *what is the motive here (male*, *28yrs)*?*”* These comments highlight the importance of understanding the trauma experience of people of color and immigrants when implementing screening for ACEs.

## Discussion

With the growing momentum to implement universal ACE screening to facilitate early intervention and improve patient outcomes, there is a pressing need to understand the best format for screening. In this study, families were randomized to complete the PEARLS tool under two modalities–an item-level response format in which caregivers disclosed which individual adversities had occurred and an aggregate-level response format in which only the total number of adversities was disclosed. Disclosure rates by screening modality as well as a caregiver experience and preferences with each screening format were examined with clear differences when comparing the qualitative results of patient preference and experience with the quantitative disclosure rates.

Adversities were reported by approximately three-quarters of caregivers (73.5% item-level, 78.6% aggregate format). While mandated reporting is commonly cited as a screening concern [31–33], screening for PEARLS led to only one new reported CPS case. In qualitative post-screening interviews, caregivers expressed a strong preference for the item-level response format of the PEARLS tool, regardless of screening arm. Caregivers described their desire to talk with providers about the adversities they endorsed because they want their providers “to do something about it.” Another important theme was the strength of the trusted relationship between providers and patients. Yet, in agreement with the only identified pediatric study of adversity screening methodology to date [9], quantitative data showed that PEARLS screening disclosure rates were higher among families that completed the aggregate-level response format for both the ten original ACEs, the related life events, and the PEARLS score. Results of the current study expand understanding of differential disclosure rates by screening format by pointing to specific adversity items in which disclosure varied (i.e., physical abuse) as well as family demographic factors (i.e., child gender and race) in which disclosure rates were found to differ.

It is noteworthy that caregiver emotional response following screening suggested low or infrequent reports of negative affect. Higher adversity scores correlated with increased affective response (both positive and negative), but only in the item-level response screening format. In the aggregate-level response format, higher scores were not associated with a difference in affective responses. The finding of increased affective responses may relate to the emotional response of feeling understood or positive expectation of a screening outcome such as connection to services (positive affect), or possibly fear or negative emotions associated with stigma (negative affect).

The higher rates of disclosure observed in the aggregate-level response versus item-level response screening format was accentuated for children identified as black and/or male. It is well documented that racism is linked to trauma [34, 35]. Blacks, and in particular black men have higher morbidity and mortality for 7 out of 10 of the leading causes of death in the U.S [35]. Findings suggest that caregivers of young black boys may be less likely to disclose adversities via an item-level response screening tool for many reasons including lack of trust and feeling safe. Medical providers have a unique role in identifying and addressing the trauma experienced by black boys who may be at high risk for a toxic stress response. Often in the pediatric practices, providers and patients are most typically not from the same race or social class. Within the study clinic 50% of the patient population is black/non-Hispanic, while 2.5% of the pediatric faculty is black [36]. Conversations about racism and trauma, between providers and patients from divergent backgrounds can lead to discomfort and distrust, if not conducted with a trauma informed approach [37, 38]. Results support the recommendation that providers engage in conversations about race with patients and family by listening intently and using compassion and empathy. Acknowledging the trauma and supporting families is critical first step in addressing racial inequities; and will decrease the risk of doing harm during these conversations [39, 40].

### Limitations and future directions

Generalizability of the findings may be limited by fact that the study was conducted at a single urban pediatric clinic during well child checks, data comes from individuals knowingly engaged in a research study, and the study refusal rate. The refusal rate was high likely secondary to the fact that families were approached during their well child visits and the lengthy consenting process. Families reported not having enough time to extend the visit as well as needing to review the consent with caregivers who were not present at the medical appointment. Regarding study procedures, researchers engaged in analyses were not blind to randomization condition, and qualitative coding is based on field notes rather than transcripts from recordings. The sample size was modest and results require replication in other clinical settings and larger samples where additional screening consideration can be examined (e.g., method of screening, ordering of items). Finally, as is common with family/caregiver reported measures, the results of the screening were dependent upon caregiver report and as such, may be subject to under and/or over reporting. Results of the randomized study suggest that screening method (item-level response versus aggregate-level response) is one such important factor that can influence rates of disclosure.

### Clinical implications

The goal of universal screening for ACEs is to improve the health of children and adults yet many practices struggle with how to implement screening. Results point to a higher rate of disclosure of adversities and less caregiver negative emotional response to screening (for those with multiple adversities) with an aggregated level format. The role of the pediatrician is to routinely screen children at the highest risk for poor health, provide anticipatory guidance, and link families to resources and interventions. Using an aggregate-level format enables disclosure of more adversities, facilitating early detection of risk of toxic stress on a population level. Clinics may want to also consider their patient-provider relationships and access to clinic support in choosing screening practice. Clinics where the patient is often seeing a different provider every visit or where social work and mental health support is limited may also need to use an aggregate-level format. However, clinics with long-standing patient-provider relationships and/or easy access to mental health and care coordination may want to choose identified screens as a majority of families, especially those who felt a strong relationship with their provider, wanted their providers to be able to offer support specific to item-level responses. Given the increasing policy and practice recommendations to screen for adversities in primary care, this study provides important evidence about the features of item-level response and aggregate-level response screening modalities as more providers and clinics move towards universal adversity and trauma screening.

## Conclusion

When health care providers screen for trauma, screening format for childhood adversities affects disclosure rates. Families that endorsed a trusting relationship with providers were more likely to report preference for an Item-level response screening format. However, disclosure rates were significantly higher on the aggregate-level response format particularly for black boys. More research is necessary to associate screening format to disclosure rate based on gender and race/ethnicity, as well as patient preference, and the long term health outcomes associated with.

## Supporting information

S1 Checklist CONSORT 2010 checklist of information to include when reporting a randomised trial. (DOC)

S1 Table Caregiver interview of PEARLS tool experience codes and definitions. (DOCX)

S2 Table Caregiver interview of PEARLS tool format preference. (DOCX)

S1 Appendix Interview questions for de-identified vs identified ACES screen. (DOC)

S2 Appendix Application for study review. (PDF)

S3 Appendix Study application (version 1.2). (PDF)

S4 Appendix PEARLS amendments 2016–2021. (DOCX)

## Abbreviations

ACEs: Adverse Childhood Experiences
EHR: Electronic Health Record
PEARLS: Pediatrics ACE and Related Life Events Screener
BCHO: Benioff Children’s Hospital Oakland
PANAS: Positive and Negative Affect Scale
OR: Odds Ratio
SD: Standard Deviation
