# Supplementary material for: Screening for adverse childhood experiences in pediatrics: A randomized trial of aggregate-level versus item-level response screening formats
Source: PLoS One. 2022 Dec 15;17(12):e0273491. doi: 10.1371/journal.pone.0273491 (PMC9754205; doi:10.1371/journal.pone.0273491)
Supplement: S1 Table — (DOCX) [file pone.0273491.s002.docx]

**Appendix Table 1**: Caregiver interview of PEARLS tool experience codes and definitions

| **Theme** | **Codes** | **Description and Example** |
| --- | --- | --- |
| Preference | Prefer de-identified | Respondents state they prefer de-identified.  Example: “I like it anonymous”, or “a number is fine.” |
| Preference | Prefer identified | Respondents state they prefer identified.  Example: “I want my doctor to know”, or “I like the way I took it, a number doesn’t make sense.” |
| Preference | No choice | Respondent didn’t state preference for one format over the other. Example: “it doesn’t matter”, “it wouldn’t have made a difference”. |
| Screening Outcome Expectation | Outcome expectancy | Respondent states their needs will be met if known, or more information is helpful to the provider.  Example: “They can help us if they know”, or “the information would give the doctor a bigger picture.” |
| Screening Outcome Expectation | Ice breaker | Respondent states the screen allowed them to start the conversation.  Example: “it gave us something to talk about”, or “I wouldn’t have brought it up myself”. |
| Quality of Provider Relationship | Relationship | Respondents state relationship with the provider as a motivating factor in opening up.  Example: “This is the doctor for all my kids; she knows everything about us, she is so supportive or so resourceful” |
| Quality of Provider Relationship | Duration | Respondent mentions duration of relationship with the provider in making a choice for the format.  Example: “I have been with this Dr for 5 years” |
| Quality of Provider Relationship | Familiarity | Respondent states being familiar with the provider.  Example: “we have known this provider for a long time, she already knows what we have been through” |
| Quality of Provider Relationship | Trust | Respondents express trust in provider, or states the role of trust in disclosing adversities.  Example: “I trust her (the provider)”, “there is the role of trust here”. |
| Quality of Provider Relationship | Comfortable | Respondent states being comfortable talking with the provider.  Example: “I’ll tell the doctor anyway.” |
| Quality of Provider Relationship | Unfamiliarity | Respondents state not knowing well the provider, or seeing the provider for the first time, or for a short time, or new at the clinic.  Example: "This is our first time seeing this provider", "I don't really know this provider." |
| Caregiver personality /  emotional state | Attribute | Personal and emotional characteristics of the respondent  Example: “I am an open book”, or “I am a private person.” |
| Caregiver personality /  emotional state | Fear | Respondent states fear of being embarrassed, reported, or about what is going to be done with the information on the screen.  Example: “I don’t know who is going to see this information”, or “I might feel a bit embarrassed”. |
| Caregiver personality /  emotional state | Exposure | Respondent states being exposed to some adversities, or not exposed to any of the adversities.  Example: “I checked a few”, or “we have experienced none of the examples on the questionnaire.” |
