# Supplementary material for: Screening for adverse childhood experiences in pediatrics: A randomized trial of aggregate-level versus item-level response screening formats
Source: PLoS One. 2022 Dec 15;17(12):e0273491. doi: 10.1371/journal.pone.0273491 (PMC9754205; doi:10.1371/journal.pone.0273491)
Supplement: S2 Table — (DOCX) [file pone.0273491.s003.docx]

**Appendix Table 2**: Caregiver interview of PEARLS tool format preference

| Preference | Screener format and type of question | | | |  |
| --- | --- | --- | --- | --- | --- |
|  | Aggregate-level Response asked about de-identified | Item-level Response asked about identified | Aggregate-level Response asked about identified | Item-level Response asked about de-identified | Total |
|  | n = 35 | n = 38 | n = 55 | n = 54 | 182 |
| De-identified | 7 | 0 | 8 | 4 | 19 |
| Identified | 22 | 30 | 26 | 32 | 110 |
| No choice | 3 | 1 | 14 | 11 | 29 |
| No answer | 3 | 7 | 7 | 7 | 24 |
