# Supplementary material for: Screening for adverse childhood experiences in pediatrics: A randomized trial of aggregate-level versus item-level response screening formats
Source: PLoS One. 2022 Dec 15;17(12):e0273491. doi: 10.1371/journal.pone.0273491 (PMC9754205; doi:10.1371/journal.pone.0273491)
Supplement: S1 Appendix — (DOC) [file pone.0273491.s004.doc]

Interview Questions for de-Identified vs Identified ACES Screen

1. What do you think of taking the ACES questionnaire for your child knowing that your health care provider will see how you answered each questions?

Or

1. What do you think of taking the ACES questionnaire for your child knowing that your health provider will not know the individual answers. They will know the number of items endorsed, not which answers?
2. Do you feel as if this information is helpful to your health care provider?
3. Do you feel that your provider should know more or less information?
