# Supplementary material for: Screening for adverse childhood experiences in pediatrics: A randomized trial of aggregate-level versus item-level response screening formats
Source: PLoS One. 2022 Dec 15;17(12):e0273491. doi: 10.1371/journal.pone.0273491 (PMC9754205; doi:10.1371/journal.pone.0273491)
Supplement: S2 Appendix — (PDF) [file pone.0273491.s005.pdf]

Application for Study Review

Study Information

**IRB Number:**	2016-089
**Protocol Title:**	**PE**diatric **A**CEs Screening and **R**esi**L**iency **S**tudy (PEARLS)
**Principal Investigator:**	Dr. Dayna Long
Department:	Center for Community Health and Engagement
Address	UCSF Benioff Children's Hospital Oakland 4705 Shattuck Ave<br>Oakland, CA 94618
Phone:	5104283129
Fax:	
Email:	dlong@mail.cho.org
**Co-Investigator:**	Dr. Dayna Long
Department:	
Address	5220 Claremont Avenue 5220 Claremont Avenue<br>Oakland, CA 94618
Phone:	5104283129
Fax:	
Email:	Dlong@mail.cho.org
**Co-Investigator:**	Ms. Mindy Benson
Department:	UCSF Benioff Children''s Hospital Oakland
Address	Children's Hospital & Research Center Oakland 5220 Claremont Ave<br>Oakland, CA 94618
Phone:	5104284145
Fax:	
Email:	mbenson@mail.cho.org
**Co-Investigator:**	Ms. Karen Daley MA, MFTI #87525
Department:	UCSF Benioff Children''s Hospital Oakland

Address	UCSF Benioff Children's Hospital Oakland 5220 Claremont Avenue<br>Oakland, CA 94618
Phone:	5104283885
Fax:	
Email:	Kdaley@mail.cho.org
**Co-Investigator:**	Mr. Adam Davis
Department:	UCSF Benioff Children''s Hospital Oakland
Address	Ambulatory Care<br>Oakland, Ca 94609
Phone:	5104282094
Fax:	
Email:	AdDavis@mail.cho.org
**Co-Investigator:**	Dr. Laura Frame
Department:	Early Intervention Services
Address	UCSF Benioff Children's Hospital Oakland 747 52nd Street<br>Oakland, CA 94609
Phone:	5104288407
Fax:	
Email:	Lframe@mail.cho.org
**Co-Investigator:**	Dr. Rachel Gilgoff
Department:	Center for Child Protection
Address	UCSF Benioff Children's Hospital Oakland 747 52nd Street<br>Oakland, CA 94609
Phone:	5104282269
Fax:	
Email:	rgilgoff@mail.cho.org
**Study Coordinator:**	Ms. Ana Hernandez
Phone:	5104283408
Fax:	
Email:	Anahernandez@mail.cho.org
**Primary Contact Person:**	Mr. Adam Davis

Funding

☐ **No External Funding**
☐ Federal ☐ Children's Oncology Group (COG) ☑ Private Foundation<br>☐ Industry ☐ Internal Grant Program ☐ Other

**Funding Source:**	Tara Health Family Foundation
**Contract or Grant Title:**	Adverse Childhood Events Study
**Contract or Grant #:**	Not Applicable
**Address of Sponsor:**	Not available
**Contact Person:**	Ruth Shaber
**Phone:**	Not applicable
**Email:**	rshaber@tarahealthfoundation.org

Subject Category

☐	Subjects admitted strictly for research purposes. Hospitalization and laboratory costs are paid by the funding source.
☑	Research subjects receiving established medical care. Hospitalization and laboratory costs paid by third party. (except for tests performed exclusively for the study)
☐	Research subjects admitted on an industry-sponsored protocol. All costs paid by industry sponsor. Requires $2,200 IRB application and set-up fee.

ClinicalTrials.Gov Registration

Does this study need to be registered on *www.clinicaltrials.gov*? (see below)	○ No ◉ Yes

If this trial will be registered, be sure to include the following paragraph at the end of the privacy section of the consent form:

A description of this clinical trial will be available on *http://www.ClinicalTrials.gov*, as required by U.S. Law. This We site will not include information that can identify you. At most, the Web site will include a summary of the results. Yo can search this Web site at any time.

General Requirements
U.S. Public Law 110-85 (Food and Drug Administration Amendments Act of 2007 or FDAAA), Title VIII, Section 801 mandates that a "responsible party" (i.e., the sponsor or designated principal investigator) register and report results of certain "applicable clinical trials":

- Trials of Drugs and Biologics: Controlled, clinical investigations, other than Phase I investigations, of a product subject to FDA regulation;
- Trials of Devices: Controlled trials with health outcomes of a product subject to FDA regulation (other than small feasibility studies) and pediatric post-market surveillance studies.

"Applicable clinical trials" generally include interventional studies (with one or more arms) of drugs, biological products, or devices that are subject to FDA regulation, meaning that the trial has one or more sites in the U.S, involves a drug, biologic, or device that is manufactured in the US (or its territories), or is conducted under an investigational new drug application (IND).

Principal Investigator Assurance

As the Principal Investigator I have ultimate responsibility for the performance of this study, the protection of the rights and welfare of the human subjects, and strict adherence by all coinvestigators and research personnel to all IRB requirements, and all applicable federal and state regulations and laws for human subject research. I hereby assure the following:

- All named individuals on this project have read and understand the procedures outlined in the protocol.
- All experiments and procedures involving human subjects will be done under my supervision or that of another qualified professional listed on this protocol.
- No changes will be made to the approved protocol or consent form without prior IRB approval (except in an emergency to safeguard the well-being of subjects).
- Only the most current, approved, IRB stamped consent form will be used to obtain informed consent from subjects or their legally authorized representative (unless waived by the IRB).
- I will promptly report to the IRB in writing any protocol violations/deviations, unanticipated problems involving risks to subjects or others, and adverse events (AEs), within the time specified by IRB policy.
- If I will be unavailable to direct this research personally, as when on leave or vacation, I will arrange for a co-investigator to assume direct responsibility in my absence. If this is not a coinvestigator named in my absence, I will notify the IRB in writing of the responsible party.
- I will obtain continuing review before the date approval for this study expires. I understand if I fail to apply for continuing review, approval for the study will automatically expire, and study activity must cease until current IRB approval is obtained.

Dr., Center for Community Health and Engagement, Dr. Dayna Long	Signed by Dayna Long on Nov 07, 2016

Faculty Sponsor's Assurance (if applicable) ☐ NA

By my signature, I certify that the student/investigator listed on page three is knowledgeable about the regulations and policies governing research with human subjects and has sufficient training and experience to conduct this particular study in accord with the approved protocol. In addition,

- I agree to meet with the student/investigator on a regular basis to monitor study progress.
- Should problems arise in the course of the study, I agree to be available to personally supervise the student in solving them.
- I assure that the student/investigator will promptly report to the IRB any protocol violations/deviations, unanticipated problems involving risks to subjects or others, and adverse events within the time specified by IRB policy.
- If I will be unavailable, as on vacation, I will arrange an alternate faculty sponsor to assume responsibility during my absence and I will advise the IRB of such arrangements.

Dr., Dr. Dayna Long	Signed by Dayna Long on Nov 07, 2016

Statement of Financial Interests

By the signatures below, each investigator is certifying that either no financial interest exists or a complete listing of all financial interests related to the proposed project is provided. All individuals named below further acknowledge their responsibility to disclose any new reportable financial interest obtained during the term of the project. The Principal Investigator's signature also certifies that all individuals required to make disclosures have been listed below:

Do you, your spouse, or dependent children, have a financial interest in the work to be conducted under the proposed project?

Principal Investigator

Dr. Dayna Long	Signed by Dayna Long on Nov 07, 2016	No. If incorrect, please change this information on Page 2 of the Application.

Co-Investigator

Dr. Dayna Long	Awaiting Signature	
Ms. Mindy Benson	Signed by Mindy Benson on Oct 31, 2016	No. If incorrect, please change this information on Page 4 of the Application.
Ms. Karen Daley MA, MFTI #87525	Signed by Karen Daley on Oct 31, 2016	No. If incorrect, please change this information on Page 4 of the Application.
Mr. Adam Davis	Signed by Adam Davis on Oct 31, 2016	No. If incorrect, please change this information on Page 4 of the Application.
Dr. Laura Frame	Signed by Laura Frame on Oct 31, 2016	No. If incorrect, please change this information on Page 4 of the Application.
Dr. Rachel Gilgoff	Signed by Rachel Gilgoff on Nov 04, 2016	No. If incorrect, please change this information on Page 4 of the Application.

Principal Investigator's Statement of Regulatory Compliance

By my signature, I certify that:

1. Neither I nor any other investigators on this study have been suspended or debarred under the Federal Food, Drug, and Cosmetic Act, 21 USC § 335a (a) or (b) or disqualified under 21 CFR § 312.70 or § 812.119.
2. There are no pending regulatory for cause audits, investigations or proceedings involving study investigators relating to compliance with laws regarding the conduct of any clinical research.

Documentation of Investigator Education in Human Subject Research

Training in Human Research Subject Protections is required for all individuals who are participating in research activities at Children's Hospital & Research Center Oakland and Children's Hospital Oakland Research Institute. The Principal Investigator, Co-Investigators and other study staff interacting with research subjects must complete the University of Miami School of Medicine CITI Program in the Protection of Human Research Subjects to obtain IRB approval of a new study. Core Modules (Basic Course) must be completed only once, and Continuing Education Modules (Refresher Course) must be completed annually.

Basic/Refresher CITI Course Completed

Principal Investigator

Dr. Dayna Long	Yes, Please attached CITI Materials below

Co-Investigator

Dr. Dayna Long	
Ms. Mindy Benson	Yes, Please attached CITI Materials below

Ms. Karen Daley MA, MFTI #87525	Yes, Please attached CITI Materials below
Mr. Adam Davis	Yes, Please attached CITI Materials below
Dr. Laura Frame	Yes, Please attached CITI Materials below
Dr. Rachel Gilgoff	Yes, Please attached CITI Materials below

CITI Training can be completed at *https://www.citiprogram.org/Default.asp?*

Expedited Review

Do you want to submit for Expedited Review? (●) Yes ( ) No

To determine whether a research project qualifies for expedited review, the details of the protocol must indicate that the research activities fulfill requirements A and B, and one of the categories outlined under C. For a protocol to be considered for expedited review, please check A and B and the appropriate category under C.

- [x] A. The research activity poses no greater than minimal risk; ***and***

- [x] B. The identification of the subjects and/or their responses would not reasonably place the subject at risk of criminal or civil liability or be damaging to the subject's financial standing, employability, insurability, reputation, or be stigmatizing, unless reasonable and appropriate protections will be implemented so that the risks related to invasion of privacy and breach of confidentiality are no greater than minimal; ***and***

- [x] C. The project falls under one or more of the expedited categories below; **Please check all that apply.**

- [ ] **Category 1.** Clinical studies of drugs and medical devices only when condition (a) or (b) is met.

  (a). Research on drugs for which an investigational new drug application **(21 CFR Part 312)** is not required. (Note: Research on marketed drugs that significantly increases the risks or decreases the acceptability of the risks associated with the use of the product is not eligible for expedited review), or

  (b). Research on medical devices for which (i) an investigational device exemption **(21 CFR Part 812)** is not required or (ii) the medical device is cleared/approved for marketing and the medical device is being used in accordance with its cleared/approved labeling.

- [x] **Category 2.** Collection of blood samples by finger stick, heel stick, ear stick, or venipuncture as follows:

  (a) from healthy, nonpregnant adults who weigh at least 110 pounds. For these subjects, the amounts drawn may not exceed 550 ml in an 8 week period and collection may not occur more frequently than 2 times per week; or

  (b) from other adults and children, considering the age, weight, and health of the subjects, the collection procedure, the amount of blood to be collected, and the frequency with which it will be collected. For these subjects, the amount drawn may not exceed the lesser of 50 ml or 3 ml per kg in an 8 week period and collection may not occur more frequently than 2 times per week.

- [x] **Category 3.** Prospective collection of biological specimens for research purposes by noninvasive means.

  Examples: (a) hair and nail clippings in a nondisfiguring manner; (b) deciduous teeth at time of exfoliation or if routine patient care indicates a need for extraction; (c) permanent teeth if routine patient care indicates a need for extraction; (d) excreta and external secretions (including sweat); (e) uncannulated saliva collected either in an unstimulated fashion or stimulated by chewing gumbase or wax or by applying a dilute citric solution to the tongue; (f) placenta removed at delivery; (g) amniotic fluid obtained at the time of rupture of the membrane prior to or during labor; (h) supra- and subgingival dental plaque and calculus, provided the collection procedure is not more

invasive than routine prophylactic scaling of the teeth and the process is accomplished in accordance with accepted prophylactic techniques; (i) mucosal and skin cells collected by buccal scraping or swab, skin swab, or mouth washings; (j) sputum collected after saline mist nebulization.
☑ **Category 4.** Collection of data through noninvasive procedures (not involving general anesthesia or sedation) routinely employed in clinical practice, excluding procedures involving x-rays, or microwaves. Where medical devices are employed, they must be cleared/approved for marketing. (Studies intended to evaluate the safety and effectiveness of the medical device are not generally eligible for expedited review, including studies of cleared medical devices for new indications.)<br><br>Examples: (a) physical sensors that are applied either to the surface of the body or at a distance and do not involve input of significant amounts of energy into the subject or an invasion of the subject's privacy; (b) weighing or testing sensory acuity; (c) magnetic resonance imaging; (d) electrocardiography, electroencephalography, thermography, detection of naturally occurring radioactivity, electroretinography, ultrasound, diagnostic infrared imaging, Doppler blood flow, and echocardiography; (e) moderate exercise, muscular strength testing, body composition assessment, and flexibility testing where appropriate given the age, weight, and health of the individual. (Note: MRI involving introduction of a contrast medium or dye is not be eligible for expedited review.)
☑ **Category 5.** Research involving materials (data, documents, records, or specimens) that have been collected, or will be collected solely for nonresearch purposes (such as medical treatment or diagnosis). (NOTE: Some research in this category may be exempt from the HHS regulations for the protection of human subjects. 45 CFR 46.101(b)(4). This listing refers only to research that is not exempt.)
☑ **Category 6.** Collection of data from voice, video, digital, or image recordings made for research purposes.
☑ **Category 7.** Research on individual or group characteristics or behavior (including, but not limited to, research on perception, cognition, motivation, identity, language, communication, cultural beliefs or practices, and social behavior) or research employing survey, interview, oral history, focus group, program evaluation, human factors evaluation, or quality assurance methodologies. (NOTE: Some research in this category may be exempt from the HHS regulations for the protection of human subjects. 45 CFR 46.101(b)(2) and (b)(3). This listing refers only to research that is not exempt.)

Requests for Waivers

Applying for:

- ☐ Partial Waiver of HIPAA Authorization
- ☐ Complete Waiver of HIPAA Authorization
- ☑ Not Requesting HIPAA Waiver

Waiving informed consent for research much satisfy Federal regulations for Protection of Human Subjects 45 CFR 46 and Standards for Privacy of Individually Identifiable Health Information (a.k.a. HIPAA) 45 CFR 164.512.

- ☐ Waiver of Consent
- ☐ Waiver of Documentation of Consent
- ☑ Not Requesting Waiver of Consent

Summary Information

1. Age Range of Eligible Subjects: 3 mths to 11 yrs, 11 mths & their adult caregivers

2. Subject Population: (Please check all that apply)

☐	a. neonates	☑	f. minors	☐	k. cancer patients
☑	b. minorities/immigrants	☑	g. non-English speaking	☐	l. terminally ill

☑	c. normal volunteers	☑	h. students	☐	m. wards of the court
☐	d. institutionalized	☐	i. prisoners or parolees	☐	n. pregnant women
☐	e. decisionally impaired	☑	j. other adult parents/guardians (ages 18 and over)		

3. Study Type: If the research involves any of the following, please check all that apply.

☐	a. Investigator-sponsored IND or IDE. A Sponsor-Investigator is an individual who both initiates and conducts a clinical trial. Regardless of funding source, the individual has the responsibilities of both a sponsor and investigator.		
☐	b. Investigational Drug (IND)	☐	m. Investigational Device (IDE – HUD)
☑	c. Genetic Research (DNA)	☐	n. Vaccine Trial
☑	d. Collection of Biological Specimens for Banking	☐	o. Respiratory Treatments, Sleep Studies, PFTs for clinical purposes
☑	e. Collection of PHI (identified data) for Database	☐	p. Gene Transfer Therapy
☐	f. Collection of Remnant Surgical Specimens	☐	q. Biohazardous Waste
☐	g. Magnetic Resonance Imaging (MRI)	☐	r. Radiation (including X-ray, DXA)
☐	h. HIV Screening	☐	s. HIV/AIDS Research
☐	i. Alcohol and Drug Abuse Research	☐	t. Controlled Substances
☐	j. Acute Care Waiver of Informed Consent	☐	u. Transplantation
☑	k. Behavioral Observations	☐	v. Deception
☑	l. Surveys, Questionnaires or Psychological Testing	☑	w. Audio/Videotapes or Focus Groups
		☐	x. Other

Protocol Information

5. Data Safety Monitoring: All interventional studies involving greater than minimal risk must include a Data Safety Monitoring Plan (DSMP). A DSMP is a plan established to assure that each research study has a system for appropriate oversight and monitoring of the conduct of the study to ensure the safety of participants and the validity and integrity of the data. The DSMP should indicate specifically whether there will be a formal Data Safety Monitoring Board (DSMB) or Data Monitoring Committee (DMC).

All Investigator-sponsored IND or IDE studies must have a DSMP, including an SOP for data monitoring.

Has a Data Safety Monitoring Plan been established to review data and/or adverse events related to this study?	◉ N/A (minimal risk) ○ Yes ○ No

6. Research Sites: Except for multi-center clinical trials (e.g., industry, COG) list all sites in which the research is to be conducted. If applicable, attach letters of support from those institutions. ☐ N/A – Multi-center clinical trial
UCSFBCHO Primary Care Clinic, 5220 Claremont Avenue, Oakland CA 94609
UCSF Mission Bay Campus, Adversity BioCore (ABC) Biobank, 1550 4th Street, Bldg 19B, Room 582, San Francisco, CA 94143 Contact: Neeta Thakur, *neeta.thakur@ucsf.edu*, 415 514-9931

Center for Youth Wellness, 3450 3rd St #201, San Francisco, CA 94124
**7. Investigational drugs/devices:**

Departmental Reviews

**IRB Number: 2016-089**	**Principal Investigator:** Dr. Dayna Long
**Protocol Title:PE**diatric **A**CEs Screening and **R**esi**L**iency **S**tudy (PEARLS)	

CHORI Staff
(NOTE: IRB Staff will forward the application to the CHORI staff after initial review)

Sponsored Programs Office, Associate Director, Cheng/ Rosales	Signed by Cheng/ Rosales on Nov 28, 2016
Interim Vice President, Research Operations, David Lynch	Signed by David Lynch on Nov 28, 2016

HOSPITAL STAFF
(NOTE: PI and/or Study Coordinator must select the following departments for their review, if required)

Director CHRCO Pharmacy Services or designated Research Pharmacist	N/A
Medical Director Pulmonary Center	N/A
Medical Director Cardiology	N/A
Other CHRCO Resource	N/A
Administrative Director CRC Signature	N/A

Protocol Summary

**Protocol Version Date:** 1.0	**Amendment #:** Not applicable
**Protocol Number:**	☑ **N/A** (no number)

Please complete the requested information in the categories below. If the item does not apply to your research, please indicate that the question is not applicable. The information should be intelligible to IRB reviewers from a variety of lay and scientific backgrounds.

8. Hypothesis: Briefly explain the hypothesis(es) to be tested. If the study is not designed to test a hypothesis, simply state "None."

Children with higher Adverse Childhood Events (ACE) scores will on average have worse health symptoms and indicators such as, more frequent infections, poorly controlled asthma and behavioral difficulties.

ACEs disclosure rates and strength of associations with key outcomes will vary based on different ACEs screening formats.

Higher ACE scores will be associated with various biomarkers of stress physiology including immune function and the neuroendocrine system.

Primary Care-based preventive interventions for toxic stress will be positively associated with changes in biomarkers, self-regulation, health and well-being.

9. Purpose of the study: What are the specific scientific aims of this study?

SPECIFIC AIMS

- **To validate the ACEs screening tool by (a) assessing the validity of the tool's association with physical and behavioral health indicators and biomarkers of stress, (b) determining the internal**

consistency of the tool, and (c) assessing the feasibility of universal implementation of the screening tool in a pediatric health care setting. %u2028

- **Examine the relationships between ACEs, stress biomarkers, and symptoms in children and caregivers over time.**
- **Test whether providing primary care-based preventive interventions for children at risk of toxic stress can lead to change in biomarkers, self-regulation, behavioral and psychological outcomes for children and caregivers.**

10. Background and Significance: Include a brief summary of previous work that provides a basis for the proposed research and that supports the expectations of obtaining useful information without undue risk to human subjects. **Provide a Bibliography (References)**
This information aids IRB reviewers in assessing how valuable the project is likely to be. If graphs or tables are used to convey information, please maintain a consistent style and make sure that fonts are no less than 11-point in size. If no preliminary data are available, it may be helpful to indicate briefly why this proposed study is a reasonable starting point. Note that some IRB members are non-scientists and may not be familiar with scientific or technical terms.

Adverse Childhood Experiences (ACES) are stressful or traumatic events experienced in childhood that have been associated with poor health outcomes that extend into adulthood. The term “ACEs” was coined in 1998 following the publication of the Adverse Childhood Experiences Study (ACE study), which found that ACEs were common within the population, were associated with negative health outcomes in adulthood, and these associations occurred in a dose-response relationship (Felitti et al., 1998). Despite research showing a clear link between childhood adversity and poor health outcomes in adulthood, the pediatric medical community does not have a validated screening tool to identify children at risk. In addition, there are limited evidence-based interventions for the physiological consequences of toxic stress. Traditionally, interventions for traumatic stress have been mental health focused and little is known about whether these same interventions can effectively prevent and address the lifelong effects of toxic stress on physical disease. Lastly, while the concept of traumatic, or “toxic,” stress has been used to imply a mechanistic link between adversity, stress physiology and future poor health outcomes, the underlying mechanisms by which trauma leads to poor health are not well understood. This study begins to address these gaps. Using the medical model of identify, evaluate, educate and treat, this project will:

- Validate a pediatric ACE screening tool to identify children exposed to adversity,
- Provide anticipatory guidance to all families to further evaluate risk and educate caregivers about ACEs and toxic stress,
- Offer one of two clinic-based preventive interventions:
- care coordination to ensure that families get connected with the specific ACES-related services, or
- resiliency clinic that will target toxic stress physiology by teaching children and caregivers mindful self-regulation,
- Collect bio-specimens on all patients to begin to make correlations between ACEs, stress physiology and health outcomes.

Since the ACEs study, on-going research has supported similar findings in studies of children and adolescents. One study reported that over 90% of urban adolescent girls endorsed having at least one adverse experience, and 85% had specifically witnessed violence in their community (Lipschitz, 2000). Another study found that 67% of youth (mean age of 8) screened positive for at least one Adverse Childhood Experience (ACE). (Burke, 2011). ACEs in children have been correlated with fair or poor general health (Bethell, Newacheck, Hawes, & Halfon, 2014; Flaherty et al., 2013), illness requiring a doctor (Flaherty et al., 2013), fair or poor dental health (Bright, Alford, Hinojosa, Knapp, & Fernandez-Baca, 2015), lifetime asthma risk (Bethell et al., 2014; Wing, Gjelsvik, Nocera, & McQuaid, 2015), ADHD (Bethell et al., 2014), autism (Bethell et al., 2014), and being overweight or obese (Bethell et al., 2014; Burke, Hellman, Scott, Weems, & Carrion, 2011). In addition, studies on ACEs during childhood and adolescence have found an association between ACEs and violent behavior (delinquent behavior, bullying, physical fighting, dating violence, weapon-carrying) (Duke, Pettingell, McMorris, & Borowsky, 2010) and learning difficulties (Burke et al., 2011).

Given these health outcomes associated with childhood adversity, the American Academy of Pediatrics (AAP) policy statement calls on pediatricians to actively screen for precipitants of toxic stress(Garner et al., 2012). Pediatric providers offer a unique opportunity for identifying and ameliorating toxic stress. Pediatricians see children at regular intervals, are trained to provide anticipatory guidance to prevent and educate families about a wide variety of public health issues, and understand the important role of parents and communities in determining a child's well-being (Garner et al., 2012). Yet, there is no validated, prospective, age-appropriate ACEs screening tool. The first arm of this study aims to validate a pediatric ACEs screening tool. The creation of a scientifically validated, standard screening tool is a crucial step that will facilitate widespread screening for ACEs in clinical pediatric settings.

Screening has limited utility in the absence of effective long-term interventions, and to date there are limited evidence-based interventions for the physiological and physical health consequences of toxic stress. This study will provide all families with ACEs-specific anticipatory guidance. Families with one or more ACEs will then get randomized to one of two interventions: care-coordination or the resiliency clinic. Both of these interventions will specifically address ACEs-related issues, and will be evaluated based on mental and physical health outcomes as well as biomarker profiles before and after the intervention.

Care coordination will be based on the Family Information & Navigation Desk (FIND) Program model. The overarching goal of care-coordination is to routinely identify a family's unmet basic social needs, in this case based on a modified ACE screener, and then connect the family to appropriate community resources. This model moves beyond a focus on biomedical and risk-behavior explanations of health to understand the root causes of health inequities and provides a preventive approach to population health. The goal is to target the social and environmental factors that profoundly impact health. A recent study of the FIND program found that families who had received care-coordination with appropriate referrals had significantly decreased social needs four months after starting the program (Gottlieb, 2016). More importantly, caregivers reported significantly improved children's overall health status four months later (Gottlieb, 2016).

The resiliency clinic is a monthly, mindfulness-based, caregiver-child group intervention focused on understanding toxic stress, and the development of self-regulation and co-regulation skills in caregivers and children identified as exposed to ACEs. The curriculum is based on existing models of mindfulness intervention (Kabat-Zinn, 2005; Liehr & Diaz, 2010; Gilkerson & Gray, 2014). The structure of the caregiver-child group will be based upon 30 years of experience at UCSF Benioff Children's Hospital Oakland's Early Intervention Services, and group collaboration from pediatric medical providers, developmental and behavioral specialists, and mental health specialists. While there are studies evaluating the buffering effect of a supportive caregiver (Dozier, Peloso, Lewis, Laurenceau, & Levine, 2008; Shonkoff et al., 2012; Scheering & Zeanah, 2001), the physical and mental health benefits of mindfulness-based interventions (Black, Semple Pokhrel & Grenard, 2011; Hölzel et al., 2011;Chambers, Gullone, & Allen, 2009; Burg, Wolf, & Michalak, 2012; Linehan, 1993; Roemer, 2015; Slopen, McLaughlin, & Shonkoff, 2014), the impact of mindfulness and self-awareness training for providers (Erikson on Children, 2015; Gilkerson et al., 2016), the effectiveness of mindfulness-based interventions in adult Primary Care (Demarzo, Montero-Marin, Cuijpers, et al., 2015), the health benefits of group pediatric visits targeted to specific medical conditions such as diabetes (Edelman, Gierisch, McDuffie, Oddone, & Williams, 2015; Housden, Wong & Dawes, 2013; Wall-Haas, Kulbok, Kirchgessner, & Rovnyak, 2012) and the power of group mental health interventions (Jones, Hutchings, Bywater, & Earmes, 2007; Meezan & O'Keefe, 1998; Gross, Fogg, & Tucker, 1995; Fristad, Goldberg-Arnold, & Gavazzi, 2003; Frame, Conley, & Berrick, 2006), to our knowledge an intervention that builds on the strengths of all of these isolated interventions has not been tested in a pediatric primary care setting.

The entire research project will be anchored by the collection of bio-specimens on each child. According to the Life Course Health Development Model, "health is a consequence of multiple determinants operating in nested genetic, biological, behavioral, social, and economic contexts that change as a person develops" (Halfon, 2002) and chaos (defined as crowding, unpredictability, etc.) of the surrounding environment leads to deleterious health effects (Kamp 2013). The normal stress response to acute life events induces several physiological responses that aid in adaptation and survival. Chronic exposure to adversity may result in a disruption of these normal stress pathways and is known as the toxic stress response (TSR). Several pathways have been suggested and include inflammatory and non-inflammatory mechanisms (disrupted neuroendocrine and/or autonomic nervous system functioning), epigenetic modification, and alteration of the body's microbiome. These pathways are sensitive to

individual differences and adaptation mechanisms and aberrations in any of these pathways may negatively affect disease outcomes. An increased understanding of the multiple pathways of stress may unveil causal mechanisms that can lead to novel clinical interventions and allow for better targeting of these interventions. The biomarkers selected for this proposal are representative of the hypothesized pathways to disease and were selected based on relevance, clinical availability, and novelty (Seeman, 1997; Seeman 2001; Karlamangla, 2002).

The collection of bio-specimens on all patients enrolled in the study will deepen our understanding of the correlations between ACEs, stress physiology and health outcomes. It will ground all aspects of this project in the underlying bio-chemical and genetic links between adversity and health outcomes. Biomarkers may help evaluate the concurrent validity of the pediatric ACEs screening tool and may offer insight on the pathways to poor health outcomes as a result of exposure to ACEs. Evaluating biomarker patterns and health outcomes in relation to ACE scores may allow for the establishment of a meaningful threshold for a clinical cut-point to the ACE score. Measuring biomarkers and therefore stress physiology before and after interventions may lead to more targeted and appropriate referrals and treatment modalities.

Ultimately, this study will help advance ACE screening in the pediatric clinic setting, offer families and providers further direction in choosing ACEs-related interventions, provide insight into the underlying biochemical patterns associated with adverse childhood experiences, and lay a strong foundation for future work elucidating the specific underlying mechanisms causing childhood adversity to lead to poor mental and physical health outcomes.

References

1. Black, D. S., Semple, R. J., Pokhrel, P., & Grenard, J. L. (2011). Component Processes of Executive Function —Mindfulness, Self-control, and Working Memory—and Their Relationships with Mental and Behavioral Health. Mindfulness, 2(3), 179–185. http://doi.org/10.1007/s12671-011-0057-2
2. Bethell CD, Newacheck P, Hawes E, Halfon N. Adverse child- hood experiences: Assessing the impact on health and school engagement and the mitigating role of resilience. Health Aff (Millwood). 2014;33(12):2106-2115. doi:10.1377/ hlthaff.2014.0914. %u2028
3. Bright MA, Alford SM, Hinojosa MS, Knapp C, Fernandez-Ba- ca DE. Adverse childhood experiences and dental health in children and adolescents. Community Dent Oral Epidemiol. 2015;43(3):193-199. doi:10.1111/cdoe.12137. %u2028
4. Burke NJ, Hellman JL, Scott BG, Weems CF, Carrion VG. The impact of adverse childhood experiences on an urban pediatric population. Child Abuse Negl. 2011;35(6):408-413.
5. Burg, J. M., Wolf, O. T., & Johannes Michalak. (2012). Mindfulness as Self-Regulated Attention. Swiss Journal of Psychology, 71(3), 135–139. http://doi.org/10.1024/1421-0185/a000080
6. Chambers, R., Gullone, E., & Allen, N. B. (2009). Mindful emotion regulation: An integrative review. Clinical Psychology Review, 29(6), 560–572. http://doi.org/10.1016/j.cpr.2009.06.005
7. Demarzo, M. M. P., Montero-Marin, J., Cuijpers, P., Zabaleta-del-Olmo, E., Mahtani, K. R., Vellinga, A., … García-Campayo, J. (2015). The Efficacy of Mindfulness-Based Interventions in Primary Care: A Meta-Analytic Review. Annals of Family Medicine, 13(6), 573–582. http://doi.org/10.1370/afm.1863
8. Dozier, M., Peloso, E., Lewis, E., Laurenceau, J., & Levine, S. (2008). Effects of an attachment-based intervention on the cortisol production of infants and toddlers in foster care. Development and Psychopathology, 20(3), 845–59.
9. Duke NN, Pettingell SL, McMorris BJ, Borowsky IW. Ad- olescent violence perpetration: Associations with multiple types of adverse childhood experiences. Pediatrics. 2010;125(4):e778-e786. doi:10.1542/peds.2009-0597. %u2028
10. Edelman, D., Gierisch, J. M., McDuffie, J. R., Oddone, E., & Williams, J. W. (2015). Shared Medical Appointments for Patients with Diabetes Mellitus: A Systematic Review. Journal of General Internal Medicine, 30(1), 99–106. http://doi.org/10.1007/s11606-014-2978-7
11. Erikson on Children. (2015). A FAN for all seasons. Spring 2015
12. Felitti, V. J., Anda, R. F., Nordenberg, D., Williamson, D. F., Spitz, A. M., Edwards, V., … Marks, J. S. (1998). Relationship of childhood abuse and household dysfunction to many of the leading causes of death in adults: The Adverse Childhood Experiences (ACE) Study. American Journal of Preventive Medicine, 14(4), 245–258.
13. Flaherty EG, Thompson R, Dubowitz H, et al. Adverse child- hood experiences and child health in early adolescence. JAMA Pediatr. 2013;167(7):622-629. %u2028
14. Frame, L., Conley, A., & Berrick, J. D. (2006). "The Real Work is What They Do Together": Peer Support and Birth Parent Change. Families in Society: The Journal of Contemporary Social Services, 87(4), 509–520.

http://doi.org/10.1606/1044-3894.3566
15. Fristad, M. A., Goldberg-Arnold, J. S., & Gavazzi, S. M. (2003). Multi-family psycho-education groups in the treatment of children with mood disorders. Journal of Marital and Family Therapy, 29(4), 491–504.
16. Garner, A. S., Shonkoff, J. P., Siegel, B. S., Dobbins, M. I., Earls, M. F., Garner, A. S., … Wood, D. L. (2012). Early childhood adversity, toxic stress, and the role of the pediatrician: Translating developmental science into lifelong health. PEDIATRICS, 129(1), e224–e231. http://doi.org/10.1542/peds.2011-2662
17. Gilkerson, L., & Gray, L. (2014). Fussy babies: Early challenges in regulation, impact on the dyad and family, and longer-term implications. Alexandria, VA: In K. Brandt, B. Perry, S. Seligman, & E. Tronick (Eds.), Infant and Early Childhood Mental Health. American Psychiatric Publishing.
18. Gilkerson, L., Justice, R., Gray, L., Barnes, M., Osta, A., Pryce, J., & Wildman, A. (2016, January). Facilitating Attuned Interactions in Primary Care. Poster presented at the Erikson Institute, Chicago, IL.
19. Gottlieb LM, Hessler D, Long D, Laves E, Burns AR, Amaya A, Sweeney P, Schudel C, Adler NE. Effects of Social Needs Screening and In-Person Service Navigation on Child Health: A Randomized Clinical Trial. JAMA Pediatr. Published online September 06, 2016.
20. Gross, D., Fogg, L., & Tucker, S. (1995). The efficacy of parent training for promoting positive parent-toddler relationships. Research in Nursing & Health, 18(6), 489–499.
21. Halfon, N., & Hochstein, M. (2002). Life Course Health Development: An Integrated Framework for Developing Health, Policy, and Research. The Milbank Quarterly, 80(3), 433–479. http://doi.org/10.1111/1468-0009.00019
22. Hölzel, B. K., Lazar, S. W., Gard, T., Schuman-Olivier, Z., Vago, D. R., & Ott, U. (2011). How Does Mindfulness Meditation Work? Proposing Mechanisms of Action From a Conceptual and Neural Perspective. Perspectives on Psychological Science, 6(6), 537–559. http://doi.org/10.1177/1745691611419671
23. Housden, L., Wong, S. T., & Dawes, M. (2013). Effectiveness of group medical visits for improving diabetes care: a systematic review and meta-analysis. Canadian Medical Association Journal, cmaj.130053. http://doi.org/10.1503/cmaj.130053
24. Jones, K., Daley, D., Hutchings, J., Bywater, T., & Eames, C. (2007). Efficacy of the Incredible Years Basic parent training programme as an early intervention for children with conduct problems and ADHD. Child: Care, Health and Development, 33(6), 749–756. http://doi.org/10.1111/j.1365-2214.2007.00747.x
25. Kabat-Zinn, J. (2005). Full catastrophe living: Using the wisdom of your body and mind to face stress, pain, and illness: Fifteenth anniversary edition. New York: Bantam Dell.
26. Kamp Dush CM, Schmeer KK, Taylor M. Chaos as a social determinant of child health: Reciprocal associations? Soc Sci Med 2013.
27. Karlamangla AS, Singer BH, McEwen BS, Rowe JW, Seeman TE. Allostatic load as a predictor of functional decline. MacArthur studies of successful aging. J Clin Epidemiol 2002;55:696-710.
28. Liehr, P., & Diaz, N. (2010). A Pilot Study Examining the Effect of Mindfulness on Depression and Anxiety for Minority Children. Archives of Psychiatric Nursing, 24(1), 69–71. http://doi.org/10.1016/j.apnu.2009.10.001
29. Linehan, M. M. (1993). Skills training manual for treating borderline personality disorder (Vol. xii). New York, NY, US: Guilford Press.
30. Lipschitz DS, Rasmusson AM, Anyan W, Cromwell P, Southwick SM. (2000) Clinical and functional correlates of posttraumatic stress disorder in urban adolescent girls at a primary care clinic. J Am Acad Child Adolesc Psychiatry, 39 (9), 1104-1111.
31. Meezan, W., & O'Keefe, M. (1998). Evaluating the Effectiveness of Multifamily Group Therapy in Child Abuse and Neglect. Research on Social Work Practice, 8(3), 330–353. http://doi.org/10.1177/104973159800800306
32. Roemer, L., Williston, S. K., & Rollins, L. G. (2015). Mindfulness and emotion regulation. Current Opinion in Psychology, 3, 52–57. http://doi.org/10.1016/j.copsyc.2015.02.006
33. Scheering, M. S., & Zeanah, C. H. (2001). A relational perspective on PTSD in early childhood. Journal of Traumatic Stress, 14(4), 799–815. http://doi.org/10.1023/A:1013002507972
34. Seeman TE, Singer BH, Rowe JW, Horwitz RI, McEwen BS. Price of adaptation--allostatic load and its health consequences. MacArthur studies of successful aging. Arch Intern Med 1997;157:2259-68.%u2028
35. Seeman TE, McEwen BS, Rowe JW, Singer BH. Allostatic load as a marker of cumulative biological risk: MacArthur studies of successful aging. Proceedings of the National Academy of Sciences of the United States of America 2001;98:4770-5.%u2028
36. Shonkoff, J. P., Garner, A. S., Siegel, B. S., Dobbins, M. I., Earls, M. F., McGuinn, L., … The Committee on Psychosocial Aspects of Child and Family Health, Committee on Early Childhood, Adoption, and Dependent Care, and Section on Developmental and Behavioral Pediatrics. (2012). The lifelong effects of early childhood adversity and toxic stress. Pediatrics, 129(1), e232–e246.
37. Slopen, N., McLaughlin, K. A., & Shonkoff, J. P. (2014). Interventions to improve cortisol regulation in children: A systematic review. Pediatrics, 133(2), 312–326.

38. Wall-Haas, C. L., Kulbok, P., Kirchgessner, J., & Rovnyak, V. (2012). Shared Medical Appointments: Facilitating Care for Children With Asthma and Their Caregivers. Journal of Pediatric Health Care, 26(1), 37–44. http://doi.org/10.1016/j.pedhc.2010.06.007
39. Wing R, Gjelsvik A, Nocera M, McQuaid EL. Association be- tween adverse childhood experiences in the home and pediatric asthma. Ann Allergy Asthma Immunol. 2015;114(5):379-384.

11. Study Design: (Check all that apply).

☐ Placebo	☐ Blinded	☑ Randomized	☐ Investigational intervention without random assignment	☐ Retrospective analysis/Specimen collection/Observational

If this study has any of the formal designations below, please indicate below: ☑ N/A

Additional description of general study design. Sequentially list all procedures, drugs or devices to be used on human subjects. Describe any use of placebos and indicate whether subjects will be randomized in this study. Attach flow diagram if appropriate. **If there are any investigational drugs or biologic agents used in this study, complete and attach the FDA 1572 Form .** If this is an investigator-initiated study, attach the FDA Investigational Drug Application (FDA 1571 Form).

The PEARL study uses an experimental design to (a) validate the modified ACEs Screening Tool and (b) assess the impact of Anticipatory Guidance plus Preventive Interventions on biomarkers, physical health and mental health. The study utilizes a sample of children and caregivers receiving health care services at the UCSFBCHO Primary Care Clinic. Participants will be recruited from the population of children receiving well-child clinic visits, and randomized at two time points. (See Appendix for BARC ACEs Study Design).

The first randomization (1) will take place just after baseline data collection, into one of three ACEs Screen groups (No Screen, Identified Screen, De-Identified Screen), with the No Screen group proceeding to standard usual Care, and the Identified/De-Identified Screen groups proceeding to Anticipatory Guidance. The second randomization is for study subjects who have an ACES =>1, taking place a month after Anticipatory Guidance at T2, into one of two Preventive Intervention groups (Care Coordination or Resiliency Clinic). Baseline (T1) and 12-month follow-up (T4) data (biomarkers, health, child mental/behavioral health) will be collected on all participants. At T2 and T3 data will be collected on measures of self-regulation, co-regulation, mental/behavioral health, and understanding of toxic stress.

Participants will be asked to provide a blood, and nasal swab sample. Study questionnaires and psychological measures will be administered; clinical measurements including heart rate, blood pressure, height, weight, and waist-hip circumference will be obtained; and, specimens (blood, oral, and nasal samples) will be collected on-site and sent to UCSF for processing and storage. All serologic assays, plasma/DNA/RNA extraction, genetic and biomarker testing will take place at UCSF in the Adversity BioCore (ABC) Bank (PI: Thakur).

Our goal is to obtain blood, and nasal swab samples for measurement of serum IgE, fibrinogen, CBC w/ differentials, and for DNA/RNA extraction for analysis of telomere length, candidate genes, and social and environmental risk factors for specific health outcomes including atopic diseases (asthma, rhinitis, eczema), acute infections (upper respiratory infections, otitis media, conjunctivitis, and urinary tract infections), developmental and behavioral outcomes in the presence of adversities as measured by the ACE score.

In addition to the above measurement, biomarkers of the inflammatory response (assorted cytokines) and of the neuroendocrine response (assorted lipid and hormonal measures) will be measured on blood specimens. Lastly, to examine nasal microbiome, we will deep sequence the 16s rRNA gene from DNA from nasal and oral samples of participants.

Study Population
Enrollment Plan and Recruitment

**16. Planned Enrollment at CHRCO:** Number of subjects needed to complete the study.	700 dyads
**17. Enrollment plan:** If you expect failed screenings or subject withdrawals, will they be replaced until the appropriate numbers of subjects have completed the study? If No, explain below.	○ Yes ◉ No ○ N/A

Subjects who are screened but ineligible will be replaced but subjects who withdraw after T1 will not be replaced as the total n needed for evaluation includes drop outs. We will maintain a screening log for documenting reasons for ineligibility, or reasons for nonparticipation of eligible subjects.

18. Recruitment: What methods will be used to identify and recruit potential subjects?

Potentially eligible study subjects will be identified in the UCSFBCHO EPIC, the electronic medical records system. Each afternoon study staff will review scheduled well appointments and identify those who meet the age requirements, according to current recruitment needs. Each day's list of potential study subjects will be identified on the clinic providers schedule with a sticker indicating the patient and caregiver are eligible for recruitment. When the patient is checked into Primary Care for their appointment, registration staff will notify the Study Coordinator(s) of their presence by paging them. Study staff will then approach the caregiver and child while they are waiting for their clinic appointment, and provide them with a study flyer (see Appendix). If the family is interested, the Study Coordinator will invite them to further discuss the study and review the consent materials.

Attach a copy of all planned advertisements, flyers and letters, etc. to potential subjects. ☐ N/A

Informed Consent

19. Capacity to Consent: Will all adult subjects and/or parents/guardians have the capacity to give informed consent?
◉ Yes ○ No

20. Translation of Consent Form: On the Summary Information Page, you were asked if you would be enrolling subjects who were non-English speaking, and you answered: True
If this is incorrect, please return to the Summary Information age, and make your changes.

Please indicate below into what language(s) the consent form will be translated, and the method of translation you are requesting. **Language(s):** Spanish
☑ Certified Translator, accompanied by an "Affidavit of Accuracy". ***Required for IND/IDE study;*** may be used for any study.

☐ Qualified Translator, according to the IRB SOP "Translation of Study Documents". This process may be used for all studies that do not involve an IDE or IND.

21. Study Personnel Administering the Consent Process: Please identify by name and credentials the individual(s) who will be authorized to describe the research to subjects or their representatives, and to invite their participation. To insure that subjects give complete informed consent and are able to ask and have all questions answered regarding the nature of their participation, the personnel administering the consent must have appropriate training and background.

Roberto Mok, LVN, Study Coordinator
Cherri Harris, LVN, Study Coordinator
Ana Hernandez, MEd, Study Coordinator
Karen Daley, MFTI
Mindy Benson, MSN, PNP
Rachel Gilgoff, MD
Sally Cantrell, PhD, PsyD
Laura Frame, PhD, LCSW

Dayna Long, MD
Maoya Alqassari, BA. Study Coordinator
Rigoberto Del Torro, BA, Study Coordinator

22. Process of Consent: Please discuss how the consent process will be conducted, describing the following elements:

a. The environment and location where the informed consent will be solicited;
   Informed consent will be solicited in person at Claremont Primary Care Clinic, UCSF Benioff Children's Hospital Oakland, in a private exam room. Study staff will review the consent form with the caregiver in their language of preference (English or Spanish). All aspects of the written consent form will be discussed in detail and caregivers and children will be offered an opportunity to have all questions answered. The process will include obtaining adult consent and parent/guardian permission for child participation. Potential subjects will be offered the opportunity to have study staff leave the room so they may privately discuss their participation with family or others, before signing the consent form.
b. Opportunities for the potential subjects to discuss their participation with family or others before signing the consent form;
   The consent process will include obtaining adult consent and parent/guardian permission. It is the caregiver/child dyad that will child participate in the study. Potential subjects will be offered the opportunity to have study staff leave the room so they may privately discuss their participation with family or others, before signing the consent form.
c. How and by whom it will be determined whether the subject or their legally authorized representatives understand the information provided;
   The Study Coordinator will determine capacity to consent based upon a discussion of the study with a prospective adult subject during the consent process, followed by a series of questions to assess the person's understanding of: the purpose of the research, the foreseeable risks and anticipated benefits of study participation; the prospective adult subject's understanding of the voluntary nature of research and the elements of consent, including the right to be informed about appropriate alternative interventions.
d. **The types of forms used** (e.g., adult consent form, parental permission form, combined form, information sheet with waiver of documentation of consent)
   Adult consent form, with oral child 7-11 assent.
   Adult video consent form, with oral child 7-11 assent.

23. Assent of Minor: For subjects age 7 through 17: ☐ N/A (under 7 or adult)

a) Considering the subject's potential capacity and medical condition, what is the suggested age of the minors to provide assent?	7-11
b) Detail below whether the assent should be in writing (a separate assent form signed by the child), and/or obtained orally.	
In addition to consent of the adult subject, oral assent of minors ages 7-11 will be sought and required for participation in the study. In cases of children 7-11 years, once an adult subject has consented, the Study Coordinator will explain that it is important to obtain the child's agreement to participate, as well. The Study Coordinator will then explain in developmentally appropriate terms the nature of the Resiliency Clinic Pilot Study and what his/her experience of it would be; assess the child's understanding of this information and anything that may influence the child's evaluation of the situation; and solicit the child's agreement to participate. Assent of minors ages 7-11 will be obtained orally and documented by the Study Coordinator on the consent form and study file.	
c) Would it would be appropriate to include on the consent form a signature block for adolescents who are able to understand the adult consent form (minimal risk studies only).	○ Yes<br>◉ No
d) Detail any justification for requesting that the IRB waive assent. ☑ N/A	

24. Information Withheld from Subjects: If any information about the research purpose and design of the study will be withheld from subjects, please explain the non-disclosure and describe plans for post-study de-briefing. ☐ N/A

No information about the overall study purpose and design will be withheld. The only information withheld from study subjects will involve the nature of their random assignments. Random assignment #1, into the ACEs Identified/De-Identified/No Screen group, will be withheld until after all other baseline measures are completed. Random assignment #2, into either intervention group, will be withheld until the completion of T1 baseline data collection. The random assignment #2 information will be shared during the T2 study visit, when study staff will offer the family the opportunity to participate in the assigned intervention and schedule the T3 visit.

Risk and Benefit Assessment

25. Potential Risks and Discomforts: Describe any potential risks or likely adverse effects of the drugs, biologics, devices or procedures subjects may encounter in the study. State the potential risks – physical, psychological, social, legal or other – connected with the proposed procedures and assess their likelihood and seriousness.

Number of visits: Participation in the study will involve at least 4 study visits and up to 6 additional clinic visits. Participants may experience an inconvenience by having to show up the for a 2-hour study visit while clinical exam measurements, heel-stick/blood, oral and nasal sampling and questionnaire data are being collected.

Emotional discomfort: Answering the questionnaires and completing measures may make participants feel uncomfortable or raise unpleasant thoughts or feelings. Participants may skip questions. Psychosocial support will be available for any participant upset by the experience.

Randomization risks: Participants will be assigned to a treatment program by chance, and the treatment they receive may prove to be less effective or have more side effects than the other study treatments or other available treatments.
Participation in the Care Coordination intervention is associated with minimal risk of discomfort.
Participation in the Resiliency Clinic: is associated with a low risk of psychological distress. To date, there are no known physical risks associated with a paired psychoeducational/medical visit model. Group participation: If randomized to Group G, Resiliency Clinic, participation will include being part of a group with other families. Each group will have about 6 adult caregivers and 6 children. There is some risk that confidentiality and privacy will not be maintained by other group member(s). All group members will be asked to verbally agree to maintain the confidentiality and privacy of all members. However, because group members are not legally bound to confidentiality, it cannot be assured. Confidentiality will be discussed with all participants when the group begins.

Heel Stick and Venipuncture for Blood Sample: Withdrawal of blood via a heel stick or from a forearm vein (venipuncture) may cause pain and bruising at the site of needle puncture. There is a remote possibility of infection from the venipuncture and some people may faint when blood is drawn.

Nasal swabs: Some people may have mild discomfort with this procedure.

Testing Samples of My Child's Blood for Toxic Stress Chemicals and Genes: At the moment
there is no identifiable chemicals or 'Toxic Stress Gene' and it is unlikely that a specific toxic stress chemical or gene will ever be identified, because toxic stress is considered to be the result of genetic and environmental risk factors. Genetic and chemical marker information that results from this study does not have medical or treatment importance at this time. However, there is a risk that information about taking part in a genetic study may influence insurance companies and/or employers regarding a child's health. To further safeguard participant privacy, genetic and chemical marker information obtained in this study will not be placed in the medical record. Although child name will not be with the sample data, it will include data such as exposure to ACEs, health status, age, race and other traits. It is possible that study finding could one day help people of the same race, ethnicity, or sex as the participant. However, it is also possible through these kinds of studies that genetic traits might come to be associated with the participant's group. In some cases, this could reinforce harmful stereotypes.

Confidentiality: Participating in a survey, donating blood, oral or nasal samples may involve a loss of privacy, but participant information will be handled as confidentially as possible. Study data will be physically and electronically

secured. As with any use of electronic means to store data, there is a risk of breach of data security. No identifiers will be included in any published reports. The UCSF Principal Investigator, Dr. Neeta Thakur of Adversity BioCore Biobank, and select UCSF staff members will have access to information about participants but they will not release any identifying information to researchers using his/her blood, oral or nasal samples.

26. Safety Precautions for Minimizing Risks: Describe the procedures for minimizing any potential risks. Where appropriate, discuss provisions for ensuring necessary medical or professional intervention in the event of adverse effects to the subject.

Participants will be informed prior to consent to anticipate that study visits will take up to 2 hours.

For participants randomized to group intervention, confidentiality will be discussed with all participants when the group begins.

Discomfort as a result of heel stick, venipuncture, oral or nasal swabs will be addressed by Study Coordinators, who are medical providers.

Complete procedures will be in place to protect participant confidentiality in the storage and transfer of data (e.g., encryption, codes, and passwords.)

Participants will be informed that In the event of injury, Dr. Dayna Long will be notified. In the event of emotional distress, Dr. Laura Frame will be notified.

27. Benefit Ratio: What is the risk benefit ratio of this research, compared with available alternatives? Describe the potential benefits the subjects may receive as a result of their participation in the research and what benefits to society may be expected. ***For greater than minimal risk research involving children there must be the prospect of direct benefit to the individual subjects.***

Note: The potential benefits of the research must justify the risks to human subjects. The risk benefit ratio of the research must be at least as favorable for the subjects as that presented by standard treatments for their condition. When comparing the risk/benefit ratio of research with that of available alternatives, the alternative of doing nothing should be included in the analysis.

Participation may inform future research and practice to help mitigate the effects of toxic stress. Participants may also benefit by receiving referrals to needed resources and learning ways to manage stress and promote health. For caregivers, specifically, it could potentially increase their capacity to understand and manage the toxic effects of stress on their children.

Because of the lack of knowledge regarding the clinical significance of genotypes, respondents will not be supplied information about their genotype, and will receive no genetic counseling. In the event that a significant genetic association is discovered, we will notify the clinic and providers of this finding. Notification will come in the form of a letter in which we will summarize our findings and refer interested persons to a detailed description in the press. In addition, PIs/Dr. Thakur and collaborators of this study will make formal presentations of the results at national and local meetings.

28. Therapeutic Alternatives: What therapeutic alternative(s) are reasonably available to potential subjects should they choose not to participate in the study? These may be research or nonresearch based alternatives.

All participants who receive care as usual will be eligible for referral to mental health treatment, basic needs support, and other interventions to reduce and manage stress as needed.

Financial Considerations

29. Payment for Participation: Describe all plans to compensate subjects, including provision of services, and other reimbursements. Describe the conditions that subjects must fulfill to receive full or partial pro-rated payment.

Each participating dyad will be compensated for their time and effort with a total of $300. They will receive $100 for participation in the baseline data collection session (T1), $50 for participation in each of the T2 and T3 sessions, and $100 for participation in the 12-month follow-up data collection session (T4). Compensation will come in form of gift cards.

30. Financial Obligations of Subjects: Will subjects have to pay for any of the tests or treatments that they receive as part of the research? Please clarify who will pay for the procedures associated with the study as well as procedures that may be part of standard clinical care. Clarify that insurance and other third party payers may not cover standard procedures if they are associated with a research project.

All study procedures will be paid for by the study. Standard clinical care that may be received by subjects during study participation will be paid for by insurance or other third party payer as per standard clinic procedures.

31. Emergency Care and Compensation for Research-Related Injury: If the research presents an unknown or greater than minimal risk of illness/injury, the financial liability for the costs of care associated with the potential research related illness/injury must be specified. Industry sponsors are required to cover the cost of treating any injury caused by study drug or procedures. For nonindustry studies if no funds are set aside, please include template language from the consent form.

Research does not present unknown or greater than minimal risk of illness or injury.

Research Methods and Procedures

32. Methodology and Data Collection: Describe the research procedures that will be followed. Please list, in sequence, all study procedures, tests, and treatments required for the study. Please indicate those that are experimental and those that may be considered standard treatment. Include a detailed explanation of any experimental procedures. Include table if available. Describe all activities involving human subjects and explain the frequency and duration of each activity.

See Appendix for Study Design and Flow

T1 Baseline Data Collection

Once eligibility is confirmed and informed consent has been obtained, Study Coordinator will obtain vital signs from child: Blood pressure, resting heart rate, waist and hip circumference, height and weight (see Appendix for Age-Appropriate Biomarker and Specimen Collection Protocol)

Medical provider is informed of patient/caregiver study participation and need for extra time to complete forms prior to well-child visit.

The Study Coordinator will log into a web-enabled REDCap database and enter the child and caregiver names, dates of birth and race/ethnicities, generating one subject ID each for the child and caregiver. The subject ID's will then be entered into a separate REDCap randomization system, and the Study Coordinator will print out the subjects' random assignment #1 and insert this designation into the study folder.

While roomed, caregiver is asked to complete the following forms and measures on an electronic tablet (see Appendix for copies) in the following order. (If necessary due to clinic flow and availability of medical provider, the items marked * below can be completed after biomarker collection). Study Coordinator will offer to read questions to caregiver aloud, or to have caregiver complete forms on their own, or a combination. Paper copies will also be made available as an alternative to the electronic tablet.

Demographic Form

Health Form

PROMIS Scale

If child is randomized to either the Identified/De-Identified Screen Group:

Modified Child ACES Screen(Depending upon random assignment #1) appropriate ACEs Screen (i.e., Identified/De-Identified or NO Screen). The "Identified" ACEs Screen lists each adverse experience and displays, for the caregiver and the provider, each item endorsed on the screen. The "De-Identified" ACEs Screen lists each adverse experience and displays on the screen, for the caregiver and provider, ONLY the total number of adverse experiences although the specific items endorsed are entered into the research database. If child/caregiver is randomized to either Identified/De-Identified Screen group:

A study coordinator gives completed Modified Child ACEs screen (Identified or De-Identified) to medical provider, who reviews.

A medical provider completes well-child check and offers anticipatory guidance to ALL caregiver/child regarding ACEs and toxic stress

After the provider offers anticipatory guidance, the study mental health clinician will briefly ask the caregiver their thoughts about the Modified ACES screener. These questions will include elicititng the caregiver opinion about the way in which they answered the questions as an idenitified screen or de-identified.

If result of screen is ACEs=>1, medical provider lets caregiver know that after biomarker collection, they will meet briefly with mental health clinician to debrief, including an opportunity to discuss specific ACEs (whether they completed an "Identified" or "De-Identified" Screen) if they wish to do so.

If result of screen is ACEs=0,the well-child check is followed by study coordinator completing biomarker collection. They move into the usual care group.

All Caregiver/child meets with mental health clinician, who debriefs about well-child check, ACEs screen and other measures, and anticipatory
guidance from medical provider.

Biomarker Collection:

Summary: Participants will be asked to provide a blood,, and nasal swab sample for genetic and serologic testing of genetic and biologic markers related to adversity and the specified health outcomes. At the moment there are no identifiable chemicals or 'Toxic Stress' gene. It is unlikely that a specific toxic stress chemical or gene will ever be identified, because toxic stress is considered to be the result of genetic and environmental risk factors.

All participants will be asked if their cells may be Cryopreserved (frozen) for future cell immortalization. All participants will be asked if they would be interested in being re-contacted for future studies.

Heel Stick for Dried Blood Spot Collection: Participants that are less than 1 year (12 months) of age, a heel stick will be performed to collect 3-5 dried blood spots on filter paper. This amounts to 375-500 microliters of blood per participant. This procedure is similar to newborn screening test that occurs at birth. Specimens will be stored and later DNA/RNA and protein biomarkers related to stress will be extracted from the samples and analyzed for the presence of genes and biomarkers thought possibly to be related to stress.

Venipuncture for Blood Sample: Venipuncture is performed with a needle and syringe to draw approximately of 7 ml (participant's 1-5 years old) to 11.5 ml (participants > 5 years old) of blood from a vein in the arm. DNA/RNA and plasma will be extracted from the samples and analyzed later for the presence of genes and biomarkers thought possibly to be related to stress.

Collection of Microbiome and DNA using oral and nasal swabs: Buccal mucosal and nasal swabs will be collected from all participants using the MoBio collection tubes. The DNA will be extracted from the samples and later analyzed for the presence of genes and chemicals thought possibly to be related to adversity. The use of human bacteria will be used to determine whether bacteria of specific types of bacteria are associated with adversity.

Measurement of Genetic Ancestry: Although we will use a questionnaire to assess racial and ethnic background, among racially admixed populations such as Latinos or African Americans, most participants will not know their true racial background or racial admixture proportions. Genetic admixture can be measured and adjusted for just like any other quantitative variable. There is evidence that racial/ethnic groups have varied physiological responses to stress. Measuring Genetic Ancestry will allow for better identification of genes or proteins associated with these varies responses. This will enable future studies of adversity through an approach presently known as admixture mapping. Information about racial ancestry will not be shared with study participants.
Freeze Cells (cryopreservation): Cells will be frozen which someday may be used to analyze the type of cells and what types of genes are turned on or off (gene expression).
Cell immortalization: In addition to saving the left over samples, in some instances, the researchers may "immortalize" the cells collected from the child. This will provide additional DNA which can then be studied in future research. Cell immortalization means that the cells may be changed in a laboratory so that they will grow and divide continuously. Immortalized cells are an important resource, and if cells from thechild's sample are changed in this way, they may be used by researchers for many years in the future to develop new drugs, tests, treatments

or products. In some cases these may have potential commercial value. There are no plans to share future profits with the caregiver and child. See Appendix for Age-Appropriate Biomarker and Specimen Collection Protocol.

After completing biomarker collection, the study coordinator will:
1. Enter Study ID into randomization database for random assignment #2
2. File forms, measures & randomization #2 in study folder
3. Scheduling T2 visit 2 weeks after T1

If caregiver/child is randomized to NO ACEs Screen group:

1. Medical provider completes well-child check.
2. Well-child check is followed by Study Coordinator completing biomarker collection.
3. Study Coordinator schedules caregiver/child to return for T2 data visit visit within 2 weeks of T1. Participants will be contacted by email, text, mail and/or phone to remind of upcoming T2 data collection visit and offer to answer any questions or address concerns.

T2 Data Collection Visit

This visit will be video taped. Study Coordinator turns on video camera in exam room. Child and caregiver are roomed for at least 10 minutes, with videotaped child-caregiver interaction during vital signs (below) and any wait time in exam room.

Study Coordinator will obtain vital signs from child: Blood pressure, resting heart rate, waist and hip circumference, height and weight.

After approximately 10 minutes, video camera is turned off.

Caregiver and child are offered the option to have study staff/hospital volunteer play nearby with child for 5 minutes while caregiver completes the STROOP Color and Word Test (administered to the caregiver by the Study Coordinator)

The caregiver will be offered the choice to complete the following measures on their own, or for the Study Coordinator to administer them as brief interviews in their preferred language. Paper copies will also be made available as an alternative to the electronic tablet.

- Behavior Rating Inventory of Executive Function (BRIEF-P or BRIEF-2)
- Perceived Stress Scale (PSS)
- Brief Symptom Inventory-18 (BSI-18)
- Reflecting on Stress and Coping Questionnaire (RSCQ)
- Short Self-Regulation Questionnaire (SSRQ)
- Devereaux Early Childhood Assessment Scale (DECA-Infant/DECA-Toddler/DECA-Preschool)
- Child Behavior Checklist (CBCL 1.5-5 or 6-18)
- Adult ACES Screen
- Assessments for Asthma, Eczema, Rhinitis, Sleep and Social Needs
- Subjective Status Ladder Screener

Estimated time to completion of these 7 caregiver measures is approximately 60-75 minutes total.

While the caregiver is completing the above forms, if the child is age 2-11 years old, the Study Coordinator will administer the Minnesota Executive Function S to the child on an This measure takes approximately 4-6 minutes to complete with the child.

In a randomly selected subset of the total sample (approximately 20%), caregivers will also be asked to complete a second modified child ACEs screen. The purpose of this is for the examination of test-retest reliability.

Total estimated time for T2 visit (vitals, caregiver completed measures, and one child completed measure) is 90 –

120 minutes.

After measures are complete for the caregivers/child dyad who was randomized to the Modified Child ACES screen group and who has a child ACES score >1, the Study Coordinator describes the next randomization to the interventions (Care Coordination/Resiliency Clinic) and answers questions. Study Coordinator schedules first intervention appointment (either Care Coordination or Resiliency Clinic orientation) within 2-4 weeks of T1. Study Coordinator schedules caregiver/child to return for T3 visit within 2 weeks of last intervention appointment (approximately 7-7.5 months following consent). Participants will be contacted by email, text, mail and/or phone to remind of upcoming T3 data collection visit and offer to answer any questions or address concerns.
Following the T2 data collection visit, each group proceeds as follows:

Usual Care 1(No ACEs Screen): receives care as usual in Primary Care.

Usual Care 2(ACEs=0): receives care as usual in Primary Care.

Care Coordination: Experimental Intervention. Caregiver-child dyads will be offered 6 monthly appointments with a Care Coordinator over a 6 month period, each lasting up to 2 hours. The Care Coordination intervention is based upon the previously tested Family Information Navigation Desk (FIND) program in Primary Care at Children's, which screens for basic social needs and connects families to resources. Medical providers write and give prescriptions for Care Coordination services to caregivers. Trained staff and volunteers screen families for social issues affecting their health, including tobacco exposure, as well as for basic social needs such as access to nutrition, housing, and utilities. The Care Coordination intervention will enhance FIND by adding the capacity to provide mental health referrals and connect to supportive resources. (See Appendix for Care Coordination description).

Resiliency Clinic: Experimental Intervention. Caregiver-child dyads will participate in 6 monthly Resiliency Clinic visits over a 6 month period, each lasting a total of 2 hours. During the clinic, six caregiver/child dyads will gather in a large room in Primary Care, where a team of facilitators (medical provider, mental health clinician, developmental specialist) will address health and wellness concerns about the child, including a review of sleep, nutrition, exercise, healthy relationships and mental health, and participate in a 1 hour 20 minute child-caregiver group designed to build and practice skills in mindful self-regulation. The pedagogical methodology used will incorporate deliberately scaffolded activities in which participants are:(a) supported in rallying around a common goal (e.g., reducing the associated effects of stress), (b) participating in naming their present experience (learning about states and how to negotiate them), (c) receiving dynamic and adaptive support "real time" to utilize new skills (mindfulness-based practice), (d) engaging in age appropriate dialogues and interactions (mindful movement, music, and other presence based activities), (e) all with a planned transfer of responsibility to families, as part of the program. (See Appendix - Resiliency Clinic Pilot Sample Curriculum)

Caregivers will be asked at consent to enroll in MyChart and provide their email address, in order to be contacted between visits. Study staff will then communicate with caregivers through EPIC MyChart email. Communications will consist of checking in with caregivers, delivering weekly mini-doses of content related to mindfulness and toxic stress, and reminding them of upcoming clinic visits.

T3 Data Collection Visit

Study Coordinator turns on video camera in exam room. Child and caregiver are roomed for at least 10 minutes, with videotaped child-caregiver interaction during vital signs (below) and any wait time in exam room.

Study Coordinator will obtain vital signs from child: Blood pressure, resting heart rate, waist and hip circumference, height and weight.

After approximately 10 minutes, video camera is turned off.

Caregiver and child are offered the option to have study staff/hospital volunteer play nearby with child for 5 minutes while caregiver completes the STROOP Color and Word Test (administered to the caregiver by the Study Coordinator)

The caregiver will be offered the choice to complete the following measures on their own, or for the Study Coordinator to administer them as brief interviews in their preferred language. Paper copies will also be made available as an alternative to the electronic tablet.

- the Behavior Rating Inventory of Executive Function (BRIEF-P or BRIEF)
- the Perceived Stress Scale (PSS)
- the Brief Symptom Inventory-18 (BSI-18)
- the Reflecting on Stress and Coping Questionnaire (RSCQ)
- the Short Self-Regulation Questionnaire (SSRQ)
- the Devereaux Early Childhood Assessment Scale (DECA-Infant/DECA-Toddler/DECA-Preschool)
- Caregiver Follow up Interview.

Estimated time to completion of these 8 caregiver measures is approximately 75-90 minutes total. While the caregiver is completing the above forms, if the child is age 2-11 years old, the Study Coordinator will administer the following to the child on an Ipad the Minnesota Executive Function Scale.
In a randomly selected subset of the total sample different from the subset identified at T2 (approximately 20%), caregivers will also be asked to complete a second modified child ACEs screen. The purpose of this is for the examination of test-retest reliability.
Total estimated time for visit (vitals, caregiver completed measures, and one child completed measure is 90 – 120 minutes. Study Coordinator schedules caregiver/child to return for T4 visit within 2 weeks of 12 months following date of consent). Participants will be contacted by email, text, mail and/or phone to remind of upcoming T4 data collection visit and offer to answer any questions or address concerns.

<u>T4 Data Collection Visit</u>

Study Coordinator will obtain vital signs from child: Blood pressure, resting heart rate, waist and hip circumference, height and weight.

While roomed, caregiver is asked to complete the following forms and measures on an electronic tablet (see Appendix for copies) in the following order. Study Coordinator will offer to read questions to caregiver aloud, or to have caregiver complete forms on their own, or a combination. Paper copies will also be made available as an alternative to the electronic tablet.

Child Behavior Checklist (CBCL 1.5-5 or 6-18)

Caregiver ACEs Screen.

Modified child ACEs Screen (Identified; adapted to be retrospective including past 12 months)

Demographic form (if any updates apply i.e., household status, education, income)

Health form

Assessments for Asthma, Eczema, Rhinitis, Sleep and Social Needs

Subjective Status Ladder Screener

Study Coordinator will complete biomarker collection (see Heel Stick, Blood Draw, and Cheek and Nasal Swab instructions for T1 above; see also Appendix for Age-Appropriate Biomarker and Specimen Collection Protocol)

Electronic Medical Record
Participants will be asked and consented to provide their medical record number so that we may link relevant clinical data to the current study. Study staff will either request a report from the EHR system, or will perform a cursory chart review at study points T1, T2, T3, and T4 to record the participant's current medication list and medical problems, including those related to mental health, referrals and utilization data. We will also collect information regarding acute health events and associated antibiotic use that have occurred at the time of visit and the preceding 12 months, and up to 12 months following enrollment. Acute health events of interest include: upper respiratory infection, otitis media, conjunctivitis, and urinary tract infections.

33. Surveys, questionnaires, or psychological tests: ☐ N/A
Please describe the provisions for administering these measures, the mode of administration, the setting, and if special training or qualifications are necessary.
Caregiver self-report measures include:

- the Modified Child ACEs Screen
- Caregiver ACEs screen

Behavioral and mental health screens including

--the BRIEF-2 or BRIEF-P
-- the PSS
-- the BSI-18
-- the RSCQ
-- the SSRQ
-- the STROOP
-- CBCL1.5-5 or CBCL 6-18
--the DECA-I or DECA-T or DECA-P2

Health Screens

--Assessments for Asthma, Eczema, Rhinitis, and Sleep

Social Determinants of Health Screens

--Social Needs Assessment

--Subjective Status Ladder Screener

Child (age 2 years and older) completed measure:

Minnesota Executive Function Scale

Each of these measures will be administered by a Study Coordinator, who will offer the caregiver the option to complete the scale independently by reading it to themselves, or to have the Study Coordinator administer it as an interview in their preferred language. If the scale is completed independently the Study Coordinator will be available to answer clarifying questions and insure completeness.

These measures will be completed in the exam room.

The BRIEF-2/BRIEF-P, DECA-I/T/P and the BSI each require Level B qualification for purchase. This may be accomplished by having obtained a degree from an accredited 4-year college or university in psychology, counseling, speech-language pathology, or a closely related field plus satisfactory completion of coursework in test interpretation, psychometrics and measurement theory, educational statistics, or a closely related area; or license or certification from an agency that requires appropriate training and experience in the ethical and competent use of psychological tests. Additionally, certain health care providers may be eligible to purchase selected Level B and/or C instruments within their area of expertise. Specifically, relevant supervised clinical experience using tests (i.e., internship, residency) in combination with formal coursework (i.e., tests and measurement, individual assessment, or equivalent) qualifies a health care provider to purchase certain restricted products. Dr. Frame meetsthese qualifications, and will train and supervise Study Coordinators to administer the measures.

The STROOP requires a level C mental health qualification to purchase the measure, which is met by Dr. Frame. This qualification includes level B requirements and an advanced professional degree (with training in psychological testing) or license/certification from an agency that requires training in the use of psychological test.

The Minnesota Executive Function Scale will be administered by the Study Coordinator to the child on an Ipad, in the exam room. The caregiver may be present and will be seated behind the child to avoid interference with the child’s responses. The MEFS requires official training and certification by Reflection Sciences, to be qualified to administer.

The ACEs Screen, Modified child ACEs Screen, PSS, CBCL (1.5-5 and 6-18), and the Reflecting on Stress and Coping Questionnaire do not require special qualifications to administer, and the PI will provide training.

34. Data Storage: Please complete the following questions regarding data storage:

a. How will the data be collected and recorded? How will the data be coded to protect personal privacy?

Data collected on study subjects will remain completely confidential. Confidentiality of participants will be maintained by handling individual paper and electronic data by ID number, rather than by name, by storing all individual electronic data in a secure REDCap database and paper data in a locked file cabinet, and not disclosing individual data to anyone. Only ID numbers will be displayed on study files, and corresponding names will be kept in a separate, locked file cabinet and password-protected drive. Hard copies of all study files, including questionnaires and measures, will be kept in a locked file cabinet, in a locked room in the basement of 5275 Claremont Avenue.

Computerized records will be stored in secure databases with passwords for authorized investigators. REDCap is a secure, web-based application designed to support data capture for research studies. REDCap is HIPAA-compliant and widely used by UCSF and other researchers.

Data collection for the MEFS, BRIEF, BSI-18, CBCL and DECA measures will be administered via secure web-based electronic systems operated by the owners of each measure (Reflection Sciences; PARinc, Pearson Q-Global; Achenbach and Devereaux, respectively). Data will be coded with ID number rather than by name. For measures requiring birthdate in order to correctly apply an age-graded measure, we will round the birthdate to the first of the individual's closest birth month (i.e., 12/11/16 would be entered as 12/01/16).

Electronic data files will be sent via UCSF secure email to the UCSF statistician for data analysis and summary and stored on a HIPAA protected UCSF server. All transfer of data (which will all be de-identified) between UCSF Benioff Children's and UCSF Parnassus will occur over secure email and stored on HIPAA protected and secure drives. Video will not be sent to UCSF. It will be placed in a locked filing cabinet and the recording downloaded to a secure hard drive at UCSF Benioff Children's Hospital Oakland and then erased from the recording device. Access will be limited to study personnel and non-UCSFBCHO analysts contracted to assist in coding the video data. These analysts will view video over a secure, HIPAA-protected system and will be bound by a written confidentiality agreement.

For the Care Coordination arm, families are going to be screened and connect to basic resources, including mental health resources, using FIND*connect* © FINDconnect is a HIPAA compliant, mobile friendly platform, founded at BCHO.

Biospecimens processed by the ABC Bank at UCSF will be de-identified and issued a separate Specimen ID number that is different from the parent-study Subject ID number. Thus, the parent-study Subject ID will no longer be associated with the specimen. An encrypted key linking the Specimen ID to the Subject ID will be stored on a secure network, will be password protected, and only accessible by Dr. Thakur, and select lab research staff

Participants will be assured of complete confidentiality of genetic and other biological test results. As with all research data, information gathered by the study will be used only for aggregate analysis, and will not be released with any information that identifies research participants. Information about genotypes, in particular, will be coded and unlinked to individual respondent identifiers. The code to link respondents and their genotypes will be securely stored, and accessible only to the database programmer, to Dr. Thakur and her collaborators. Respondents will be informed that genotyping at loci related to adversities is valuable for research purposes and in aggregate form only.

Home addresses will be provided to a non-UCSF collaborator to correlate address with measure of air pollution and census data. Home addresses will be coded and unlinked to individual respondent identifiers.

Video recordings cannot by nature be completely de-identified, however they will be labeled with ID numbers; no identifying information will be included in the label. After recording a study visit, the recorder will be placed in a locked filing cabinet and the recording downloaded to a secure hard drive and then erased from the recording device. Access will be restricted to study personnel.

b. How will the data be stored during the study?

Hard copies of all study files, including questionnaires and transcripts, will be kept in a locked file cabinet, in a locked room in the basement of 5275 Claremont Avenue.
Electronic data files and video recordings will be stored on secure, HIPAA-protected UCSFBCHO and UCSF drives that are maintained in a locked file cabinet.

c. Who will have access to the data and the data codes? If data with subject identifiers will be released, specify the person(s) and agencies to whom this information will be released.

Information provided by the subjects will remain strictly confidential, with access limited to the project staff and, if applicable, State or Federal regulatory personnel.
The only exceptions to confidentiality, which are mandated by law, are detailed in the consent form. No one but select project staff (the PI, Co-I, and study coordinators) will have access to the master list linking subjects' names to code numbers, and all information obtained will be coded. Additional project staff will have access to de-identified data (coded by ID number only) for the purposes of data analysis. This ID will completely separate from any number already existing in clinic (e.g., MRN number, pt ID, etc.).

This code will be verbally recorded on video recordings and listed with all transcripts and completed surveys. The master list will be locked. Publications or presentation of findings will not include information identifying the subjects. There will be no release of identifiable data.

d. What will happen to the data when the study is completed?

Hard-copies containing participant information will be destroyed 7 years after the study closed. De-identified data will be kept on a secure network indefinitely for future analyses that will inform future studies. Biospecimens collected during this study will be deposited in the UCSF ABC Bank for analysis and for indefinite storage.

Data Analysis

35. Statistical Analysis: Please delineate the data analysis plans for this study. Include planned statistical analyses and explanation of determination of sample size. Briefly describe what statistical analysis(es) of which outcome will be applied to address each primary aim. For qualitative research, briefly describe how qualitative data will be analyzed.

Examples of statistical analyses include:

- Calculation of descriptive statistics such as mean, median, SD, range, tallies.
- Examination of graphs such as outcome vs. time, scatterplots of two variables, Kaplan-Meier curves.
- Estimation of differences between two groups with comparison by t-test or Mann-Whitney test.
- Estimation and testing of within-person changes by matched t-test or Wilcoxon signed-rank test.
- Multiple linear regression, logistic regression, or Cox proportional hazards regression.
- Repeated measures models (usually requires the help of a statistician).

All data will be merged and undergo final cleaning by the Consortium's Data Core at UC San Francisco using SAS. Our data analytic plans directly follows from our specific aims.
Sample Size/Power: Sample size and power estimates for comparison of the two ACEs formats in Phase 3 are based on alpha = 0.05 and 2-sided t-tests. Estimates of associations between ACE items with child biomarkers, and behavioral health outcomes in similar studies suggest effect sizes that are small to moderate, with standardized effects ranging from d=0.30 to 0.48. [1,2,3,4] Effect sizes for associations between ACEs or early psychosocial exposures with health diagnoses in related adult and child literatures similarly suggest small to moderate effect sizes of OR=1.6-1.9 across a range of diagnoses (e.g., acute upper respiratory infections, otitis media, viral infections, eczema, UTI, and asthma). [5,6] We intend to obtain a sample of n=555 (n=185 per arm). This sample size will provide power to exceed 0.80 for evaluation of the association between ACEs with the biomarkers, child behavioral health

indicators, and child diagnoses. Within the Identified arm, the ratio of ACEs items to respondents exceeds 5.0 for factor analytic techniques.[7,8]For tests directly comparing between group differences across arms (identified vs. de-identified screening format), this sample size (n = 370 total, n=185 per arm) will additionally allow for detection of an approximate 11% difference in disclosure rates for a given number of ACEs [4] or standardized units mean difference in number of ACEs. At the projected sample size for each group, power to discriminate at this level between the two arms will be at least 80%. The total number of patients who came to CPCC for well visits in 2013 was 8,714. 12% were Spanish speakers. Of all patients, 4,760 were ages 0-3, 971 ages 4-5, and 2,160 ages 6-12. Given the large number of patients seen annually at CPCC, coupled with our previous success implementing the FIND study, we do not expect to have difficulty recruiting this sample size.

Descriptive Analyses & Missing Data: Descriptive analyses will include family demographics, ACEs (parent self-report and report of child), biomarkers, and assessments of children's symptoms, behavior, and physical health. In our analytic plan, we examine ACE scores by: a continuous total score, individual items, and with subscale factor scores and a cut-point score pending the results of the validation study analyses and where appropriate. Data will be inspected for outliers and out-of-range values. Examination of distributions may prompt transformations, where they are defensible and are a component of the best available analysis strategy. Following recommendations by Dube and colleagues, missing ACEs data will be conservatively considered not to have that experience. Likelihood based approaches (e.g. GLMMs) and multiple imputation will be used to handle missing data for other variables . Both approaches fit models to all available data and invoke the relative assumption that the data are missing at random. Background demographics will be examined to describe the sample and will be included in multivariate analysis if they are related to the outcome at $p<.2$, differ between treatment arms, or associated with dropout. Correlations among variables within each area will be examined and redundant measures will be combined or eliminated to avoid multicollinearity.
Specific Aim 1: Cross sectional and longitudinal associations between ACEs, biomarkers, health symptoms and mental/behavioral health indicators in children and parents: Cross sectional associations will be examined with linear mixed models that include intervention group as a predictor. Generalized linear mixed models (GLMMs) will examine the correlations between parent and child ACE scores, biomarkers, and symptoms across time . First, each measure will be modeled separately to determine whether it increased, decreased, or remained constant over time both within and across study arms. Once successfully modeled separately, they will be modeled in two ways: to examine the predictive effect of change in one measure on another over time and vice versa (unidirectional regression model), and to determine whether change in one measure and change in another significantly co-varied together over time (bidirectional regression model). For both models, intervention group will be included as a covariate to assess for differential effects by study arm, as well as key covariates identified in preliminary analyses.
Specific Aim 2: The Impact of Intervention on Child and Caregiver Outcomes: We will fit regression models for cross-sectional and longitudinal outcomes. Demographics will be compared for the usual care, and the intervention arms at baseline. Comparisons of the Usual Care and Intervention arms on changes in biomarkers will be estimated with linear mixed models and generalized linear models with generalized estimating equations (e.g., SAS PROC MIXED ROC GENMOD, SAS Institute Inc., 1999). Similar models will be conducted for additional outcomes including: caregiver understanding of toxic stress and the buffering role of parenting, as well as openness to help-seeking. Generally, we seek to model longitudinally the trajectory of these outcomes as a function of time, intervention group assignment, and group-by-time interaction. We begin by fitting base models, those with minimum covariates and, for longitudinal models, the most restricted residual covariance structures. Additional covariates then will be considered (including demographic variables, referrals and services). Empirical contributions to model selection decisions will include reference to information criteria (e.g., Akaike's). We will examine change from baseline for each outcome as the dependent variable. In addition to testing the groups-by-time interaction term, custom models and contrasts will assess group differences at each time point. Change from baseline to 6 week and 12 month follow up will allow for testing the short and long term effects of the brief targeted intervention. Primary tests will compare participants assigned to the intervention arm vs. participants assigned to usual care with an ACE score of ≥ 1. Participants assigned to the usual care arm with an ACE score of 0 will additionally be explored as a non-exposed control comparison group.
Moderating Analyses: These analyses will further elaborate any observed treatment effects. A first set will determine whether demographic strata, baseline ACE score, biomarkers, or intervention dose effects moderate any intervention effects. Each outcome will be regressed onto measures of intervention, baseline or other covariates and their interactions with intervention group assignment. In these models, a significant interaction

term suggests that the intervention effect is moderated by the corresponding variable.

References

Evans, S. E., Davies, C., & DiLillo, D. (2008). Exposure to domestic violence: A meta-analysis of child and adolescent outcomes. Aggression and Violent Behavior, 13(2), 131-140

Cicchetti, D., Rogosch, F. A., Gunnar, M. R., & Toth, S. L. (2010). The Differential Impacts of Early Physical and Sexual Abuse and Internalizing Problems on Daytime Cortisol Rhythm in School--%u2010Aged Children. Child development, 81(1), 252-269.

Finklehor D. 2013

Theall K, Drury S, Shirtcliff E. Cumulative Neighborhood Risk of Psychosocial Stress and Allostatic Load in Adolescents. Am J Epidemiol. 2012;176(Suppl):S164–S174.

Felitti VJ, Anda RF, Nordenberg D, Williamson DF, Spitz AM, Edwards V, Koss MP, et al. The relationship of adult health status to childhood abuse and household dysfunction. Amer J of Prev Med. 1998; 14:245:258

Karlen, J., Ludvigsson, J., Hedmark, M. Farsjo, A., Therodorsson, E., Faresjo, T. Early Psychosocial exposures, hair cortisol level and disease risk. Pediatrics. 2015. 135, e1450-57

Bryant FB and Tarnold PR. Principle components analysis and exploratory and confirmatory factor analysis. . In Reading and understanding multivariate statistics. Grimm LG and Tarnold RR Eds. Washington, D.C., American Psychological Association, 1995, p. 99-136.

Comrey AL and Lee HB. A first course in factor analysis. Hilsdale, N.J., Erlbaum, 1992

Comments/ Remarks (optional)

This is a large multidisciplinary cross sector research project. We are eager to get start and to learn how to better prevent and manage trauma in our patients.

Attachments

Please list Attachments, Supplements and Appendices, including Version(s) and date(s). Include HIPAA Authorization Forms (if applicable) and any other material to be given to subjects.

- **PEARLS Protocol Summary**
- **Appendix- Specimen Collection AGE 0-1**
- **Appendix- Specimen Collection AGE 1-5**
- **Appendix- Specimen Collection AGE 5plus**
- **Appendix- Dried Blood Spot Poster**
- **Appendix- PEARLS Video Consent Revised**
- **Appendix- PEARLS Measures copy and table**
- **Appendix- PEARLS Flyer**
- **Appendix- PEARLS Flow Revised**
- **Appendix- PEARLS Eligibility Screen Revised**
- **Appendix- PEARLS Design Revised**
- **Appendix- PEARLS Consent Revised**
- **Appendix- HIPAA**

- **Appendix- Health and Demographics Form Revised**
- **Appendix- Data Collection Schedule**
- **Appendix- Caregiver ACE Study Questionnaire Revised**
- **Appendix- Resiliency Clinic Pilot Sample Curriculum**
- **Appendix- BARC Child ACE Questionnaire V8 De-identified**
- **Appendix- BARC Child ACE Questionnaire V8 Identified**
- **Appendix- PROMIS Sclae v1.0**
- **Appendix- PEARLS Interview Questions**
- **Request for Research Records**

Study Abstract (for chori website)

Provide an abstract/full synopsis of this study to be posted on the CHORI Website. Examples are at: *http://www.chori.org/Clinical_Studies/Active_Studies/active_studies_home.html*

Stressful and traumatic experiences in childhood (Adverse Childhood Events, or ACEs) have been associated with poor health outcomes that extend into adulthood. When stress is sustained or severe in the absence of an adequate buffer, the stress response can become dysregulated--a state referred to as toxic stress. Some professional organizations have advocated for ACEs screening to be part of routine medical care. To date, however, no ACEs screening tool has been validated for use with children. Intervening early at critical points in the life course has the potential to allow a child to avoid the negative consequences of these adverse events. **PE**diatric **A**CEs Screening and **R**esi**L**iency **S**tudy (PEARL) aims to (1) To validate the ACEs screening tool by (a) assessing the validity of the tool's association with physical and behavioral health indicators and biomarkers of stress, (b) determining the internal consistency of the tool, and (c) assessing the feasibility of universal implementation of the screening tool in a pediatric health care setting. (2) Examine the relationships between ACEs, stress biomarkers, and symptoms in children and caregivers over time.Test whether providing primary care-based preventive interventions for children at risk of toxic stress can lead to change in biomarkers, self-regulation, behavioral and psychological outcomes for children and caregivers. At the end of the study, we will have validated an ACEs screening tool suitable for use in a pediatric clinic, and we will have evaluated the effectiveness of two different interventions for children exposed to ACEs. The long-term goal is to promote screening for and intervention on adverse childhood events a standard part of pediatric health care delivery.

Research involving children is governed by 45 CFR 56 Subpart D. Children are considered a vulnerable research population because their intellectual and emotional capacities are limited. The regulations specify the following four categories of permissible child research and the requirements that must be met under each category. Please check the applicable category for your research project and indicate how this research project addresses the issues listed in that category. You may use the remarks section and/or attach additional sheets as necessary.

☑ **1. Research not involving greater than minimal risk. §46.404**

The following requirements must be met:

a. Describe how the potential risks are outweighed or balanced by the potential benefits to the subject or society.

The potential benefits to subjects participating in this study include 1) the identification of adverse childhood experiences and therefore the chance to receive support and services through the primary care clinic to mitigate the mental and physical health consequences, 2) the opportunity to learn about the link between ACEs and health, 3) links to ACEs-related referrals and/or the development of skills to manage stress and its health impacts, and 4) for caregivers to increase capacity to understand and manage the potentially toxic effects of stress on their children.

While caregivers may feel that mandated reporting requirements to Child Protective Services and/or law enforcement is a risk, mandated reporting is a benefit to the child being abused or neglected.

Discussing adversity and trauma may be triggering and emotionally charged for a number of caregivers and children, however, the primary care department staff including medical providers and social workers, will be available to help support awareness, education, growth and recovery.

Blood draws to collect bio-specimens will hurt a little and will possibly leave a bruise. However, elucidating the associations between adversity in childhood, biomarkers of stress physiology and lifelong health impacts will provide benefits to society by 1) providing insight into the underlying biochemical and immunologic pathways between stress and health, 2) helping lay the foundation for multi-sector, multi-disciplinary approaches to mental and physical health and well-being, and 3) guiding future prevention work and interventions related to adverse childhood experiences.

b. Indicate how adequate provisions have been made for soliciting assent of the children and permission of the parents or guardians.

The research team conducting this study holds a high standard of respect for all adult and child participants, which includes transparency about the purpose, possible risks and benefits to subjects. Informed consent of adult participants (parents/guardians) will be afforded as much time as is necessary for both parents/guardians and children to be informed about what their participation in the study would entail and given an opportunity to ask questions and have them answered in a developmentally appropriate way. For children ages 7-11, declining assent will constitute declining participation in the study even if parents/guardians consent.

☐ **2. Research involving greater than minimal risk but presenting the prospect of direct benefit to the individual subjects. §46.405**

☐ **3. Research involving greater than minimal risk and no prospect of direct benefit to individual subjects, but likely to yield generalizable knowledge about the subject's disorder or condition. §46.406**

☐ **4. Research not otherwise approvable which presents an opportunity to understand, prevent, or alleviate a serious problem affecting the health or welfare of children. §46.407**
