# Supplementary material for: Screening for adverse childhood experiences in pediatrics: A randomized trial of aggregate-level versus item-level response screening formats
Source: PLoS One. 2022 Dec 15;17(12):e0273491. doi: 10.1371/journal.pone.0273491 (PMC9754205; doi:10.1371/journal.pone.0273491)
Supplement: S3 Appendix — (PDF) [file pone.0273491.s006.pdf]

Study Application (Version 1.2)

1.0 General Information

***Enter the full title of your study:**

PEdiatric ACEs Screening and ResiLiency Study (PEARLS)

***Enter the study number or study alias**

PEARLS
* This field allows you to enter an abbreviated version of the Study Title to quickly identify this study.

2.0 Add Department(s)

2.1 List the departments associated with this study. The Principal Investigator's department should be Primary.:

Primary Dept?	Department Name
◆	**UCSF** - *728050* - FQHC PRIM CARE-CLRMT

3.0 List the key study personnel: (Note: external and affiliated collaborators who are not in the UCSF directory can be identified later in the Qualifications of Key Study Personnel section at the end of the form)

3.1 *Please add a Principal Investigator for the study:

Long, Dayna MD

Select if applicable

- [x] Department Chair
- [ ] Resident
- [ ] Fellow

If the Principal Investigator is a Fellow, the name of the Faculty Advisor must be supplied below.

3.2 If applicable, please select the Research Staff personnel:

A) Additional Investigators

Benson, Mindy S
Other Investigator
Hessler, Danielle, PhD
Co-Principal Investigator
Jeung, Joan J
Other Investigator
Thakur, Neeta MD
Co-Principal Investigator

B) Research Support Staff

Ajero, Nai F
Study Coordinator
Alqassari, Maoya M
Study Coordinator
De La Rosa, Rosemarie
Research Assistant
Harris, Cherri L
Study Coordinator
Mok, Roberto
Study Coordinator
Yee, Morgan M
Data Manager

3.3 *Please add a Study Contact:

Mok, Roberto

The Study Contact(s) will receive all important system notifications along with the Principal Investigator. (e.g. The project contact(s) are typically either the Study Coordinator or the Principal Investigator themselves).

3.4 If applicable, please add a Faculty Advisor/Mentor:

3.5 If applicable, please select the Designated Department Approval(s):

Add the name of the individual authorized to approve and sign off on this protocol from your Department (e.g. the Department Chair or Dean).

4.0 Initial Screening Questions

Updated December 2020 - Revised Common Rule (January 2018) Compliant / COVID-19 - v95

4.1 * PROJECT SUMMARY: (REQUIRED) Give a brief overview of this project (250 words or less). Tell us what this study is about, who is being studied, and what it aims to achieve. If you have an NIH Abstract, paste it here (Click on the orange question mark to the right for more detailed instructions):

Stressful and traumatic experiences in childhood (Adverse Childhood Events, or ACEs) have been associated with poor health outcomes that extend into adulthood. When stress is sustained or severe in the absence of an adequate buffer, the stress response can become dysregulated--a state referred to as toxic stress. Some professional organizations have advocated for ACEs screening to be part of routine medical care. To date, however, no ACEs screening tool has been validated for use with children. Intervening early at critical points in the life course has the potential to allow a child to avoid the negative consequences of these adverse events.

The proposed study has three overarching aims: (1) Examine the relationship between ACEs, stress biomarkers, and symptoms in children and caregivers over time; (2) Validate an ACEs screening in a pediatric health care setting; and (3) Test whether providing primary care-based preventive interventions for children with or at risk for toxic stress can lead to detectable changes in biomarkers, behavior, or health outcomes for children and/or caregivers.

4.2 * HUD DEVICE: (REQUIRED) Does this application involve a Humanitarian Use Device (HUD):

- (•) No
- ( ) Yes, and it includes a research component
- ( ) Yes, and it involves clinical care ONLY

4.3 * TYPE OF RESEARCH: (REQUIRED) Select the option that best fits your project (Click the orange question mark to the right for definitions and guidance):

- ( ) Biomedical research (including medical records review, biospecimen collection and/or use, other healthcare or health outcomes related activities, research database, biospecimen bank, or recruitment registry)
- ( ) Social, behavioral, educational, and/or public policy research
- (•) Hybrid - includes aspects of BOTH types of research (check this option if your research is mainly social /behavioral but also involves specimen collection or blood draws to look at biological measures)

4.4 * SUBJECT CONTACT: (REQUIRED) Does this study involve ANY contact or interactions with participants:

- (•) Yes (including phone, email or web contact)
- ( ) No (limited to medical records review, biological specimen analysis, and/or data analysis)

4.5 * RISK LEVEL: (REQUIRED) What is your estimation of the risk level, including all screening procedures and study activities:

- (•) Minimal risk
- ( ) Greater than minimal risk

4.6 * REVIEW LEVEL: (REQUIRED) Requested review level (Click on the orange question mark to the right for definitions and guidance):

- ( ) Full Committee
- (•) Expedited
- ( ) Exempt

4.7 * EXPEDITED REVIEW CATEGORIES: (REQUIRED) If you think this study qualifies for expedited review, select the regulatory categories that the research falls under: (check all that apply)

- [ ] Category 1: Research using approved drugs or devices being used for their approved indications
- [x] Category 2: Collection of blood samples by finger stick, heel stick, ear stick, or venipuncture in certain populations and within certain amounts
- [x] Category 3: Prospective collection of biological specimens for research purposes by noninvasive means (e.g. buccal swabs, urine, hair and nail clippings, etc.)
- [x] Category 4: Collection of data through noninvasive, routine clinical procedures (e.g. physical sensors such as pulse oximeters, MRI, EKG, EEG, ultrasound, moderate exercise testing, etc. - no sedation, general anesthesia, x-rays or microwaves)
- [x] Category 5: Research involving materials (data, documents, records, or specimens) that have been or will be collected solely for nonresearch purposes
- [x] Category 6: Collection of data from voice, video, digital, or image recordings made for research purposes
- [x] Category 7: Research on individual or group characteristics or behavior or research employing survey, interview, oral history, focus group, program evaluation, human factorsevaluation, or quality assurance methodologies

* Does the collection of blood samples meet requirements outlined by HHS Office for Human Research Protections for **Expedited Review Research Category 2**: **(REQUIRED)**

- For healthy, nonpregnant adults who weigh at least 110 pounds the amounts drawn may not exceed 550 ml in an 8 week period and collection may not occur more frequently than 2 times per week;
- From other adults and children, considering the age, weight, and health of the subjects, the collection procedure, the amount of blood to be collected, and the frequency with which it will be collected, the amount drawn may not exceed the lesser of 50 ml or 3 ml per kg in an 8 week period and collection may not occur more frequently than 2 times per week

(•) Yes ( ) No

4.9 *** DATA/SPECIMEN ANALYSIS ONLY: (REQUIRED) Does this study ONLY involve records review and /or biospecimen analysis (do not check 'Yes' if this is a registry, research or recruitment database, or biospecimen repository):**

( ) Yes (•) No

4.10 *** CLINICAL TRIAL: (REQUIRED)**
Is this a clinical trial:

According to The World Health Organization (WHO) and the International Committee of Medical Journal Editors (ICMJE) a clinical trial is:

- Any research study that prospectively assigns human participants or groups of humans to one or more health-related interventions to evaluate the effects on health outcomes.

ICMJE requires registration of a clinical trial in a public database (such as ClinicalTrials.gov) prior to enrollment, for eventual publication of results in member biomedical journals.
Guidance: Public Law 110-85 requires that all investigators who perform an *applicable clinical trial* must ensure that the trial is registered on a government web site called **ClinicalTrials.gov**.

The FDA requires registration for 'applicable clinical trials,' defined as follows:

- For any trials of drugs and biologics: controlled clinical investigations, other than Phase 1 investigations, of a product subject to FDA regulation.
- For trials of biomedical devices: controlled trials with health outcomes of devices subject to FDA regulation, other than small feasibility studies, and pediatric post-market surveillance.

For additional information on the **ClinicalTrials.gov** registration process at UCSF and the definition of a clinical trial for purposes of registration, visit the **ClinicalTrials.gov section of the UCSF Clinical Research Resource HUB**.

(•) Yes ( ) No

Clinical Trial Registration - 'NCT' number for this trial:

NCT04182906

4.11 * CLINICAL TRIAL PHASE: (REQUIRED) Check the applicable phase(s):

- [ ] Phase 0
- [ ] Phase 1
- [ ] Phase 1/2
- [ ] Phase 2
- [ ] Phase 2/3
- [ ] Phase 3
- [ ] Phase 4
- [x] Not Applicable

4.12 * INVESTIGATOR-INITIATED: (REQUIRED) Is this an investigator-initiated study:

(•) Yes ( ) No

The UCSF IRB recommends use of the Virtual Regulatory Binder to manage your study.

4.13 * CORONAVIRUS RESEARCH: (REQUIRED) Does this study involve research on coronaviruses (COVID-19, SARS, MERS or other):

( ) Yes (•) No

4.15 * CANCER: (REQUIRED) Does this study involve cancer (e.g., the study involves patients with cancer or at risk for cancer, including behavioral research, epidemiological research, public policy research, specimen analysis, and chart reviews):

( ) Yes (•) No

4.16 * RADIATION EXPOSURE: (REQUIRED) Does your protocol involve any radiation exposure to patients /subjects EITHER from standard care OR for research purposes (e.g., x-rays, CT-scans, DEXA, CT-guided biopsy, radiation therapy, or nuclear medicine including PET, MUGA or bone scans):

( ) Yes (•) No

4.17 SCIENTIFIC REVIEW: If this study has undergone scientific or scholarly review, please indicate which entity performed the review (check all that apply):

- [ ] Cancer Center Protocol Review Committee (PRC) (Full approval is required prior to final IRB approval for cancer-related protocols.)
- [ ] CTSI Clinical Research Services (CRS) Advisory Committee
- [ ] CTSI Consultation Services
- [ ] Departmental scientific review
- [ ] Other:

4.18 * STEM CELLS: (REQUIRED) Does this study involve human stem cells (including iPS cells and adult stem cells), gametes or embryos:

(•) No

○ Yes, and requires IRB and GESCR review
○ Yes, and requires GESCR review, but NOT IRB review

4.19 * **FINANCIAL INTERESTS: (REQUIRED) Do you or any other responsible personnel (or the spouse, registered domestic partner and/or dependent children thereof) have financial interests related to this study:**

○ Yes ◉ No

5.0 Funding

5.1 * **FEDERAL FUNDING: (REQUIRED) Is this study currently supported in whole or in part by Federal funding, *even by a subcontract*, OR has it received ANY Federal funding in the past:**

○ Yes ◉ No

5.2 * **DoD INVOLVEMENT: Is this project linked in any way to the Department of Defense (DoD): (REQUIRED)**

○ Yes ◉ No

5.3 **SPONSORS: Identify all sponsors and provide the funding details. If funding comes from a Subcontract, please list only the Prime Sponsor:**

External Sponsors:

View Details	Sponsor Name	Sponsor Type	Awardee Institution	Contract Type:	UCSF RAS "P number" or eProposal number	UCSF RAS System Award Number ("A" + 6 digits)
⊟	Center for Youth Wellness	07	UCSF	Subcontract		A126349

Sponsor Name:	Center for Youth Wellness
Sponsor Type:	07
Sponsor Role:	Funding
**CFDA Number:**	
**Grant/Contract Number:**	
Awardee Institution:	UCSF
**Is Institution the Primary Grant Holder:**	No
**if No, then who is the Primary Grantee?**	
Contract Type:	Subcontract
UCSF RAS "P number" or eProposal number:	
UCSF RAS System Award Number ("A" + 6 digits):	A126349
Grant Number for Studies Not Funded thru UCSF:	

Grant Title:	
PI Name:<br>(If PI is not the same as identified on the study.)	
Significant Discrepancy:	

⊟	Tara Health Foundation	07	CHORI	Subcontract		

Sponsor Name:	Tara Health Foundation
Sponsor Type:	07
Sponsor Role:	Funding
**CFDA Number:**	
**Grant/Contract Number:**	
Awardee Institution:	CHORI
**Is Institution the Primary Grant Holder:**	No
**if No, then who is the Primary Grantee?**	
Contract Type:	Subcontract
UCSF RAS "P number" or eProposal number:	
UCSF RAS System Award Number ("A" + 6 digits):	
Grant Number for Studies Not Funded thru UCSF:	
Grant Title:	
PI Name:<br>(If PI is not the same as identified on the study.)	
Significant Discrepancy:	Funding is through Tara Health Foundation via subaward from Center for Youth Wellness.

Other Funding Sources and Unfunded Research - Gift, Program, Departmental or other Internal Funding (check all that apply):

- ☐ Funded by gift (specify source below)
- ☐ Funded by UCSF or UC-wide program (specify source below)
- ☐ Specific departmental funding (specify source below)
- ☐ Unfunded (miscellaneous departmental funding)
- ☐ Unfunded student project

6.0 Sites, Programs, Resources, and External IRB Review

6.1 * UCSF AND AFFILIATED SITES (check all that apply): (REQUIRED)

- ☑ UCSF Benioff Children's Hospital Oakland (BCHO)
- ☐ UCSF Cancer Center Berkeley
- ☐ UCSF Cancer Center San Mateo
- ☐ UCSF China Basin clinics and facilities
- ☐ UCSF Helen Diller Family Comprehensive Cancer Center
- ☐ UCSF Langley Porter Psychiatric Institute (LPPI)
- ☐ UCSF Medical Center at Mission Bay (Benioff Children's Hospital, the Betty Irene Moore Women's

Hospital, Bakar Cancer Hospital, or outpatient clinics)
- [ ] UCSF Mount Zion
- [ ] UCSF Parnassus (Moffitt-Long hospital, dental clinics or other outpatient clinics)
- [ ] UCSF Other Sites (including Laurel Heights and all the other sites outside the main hospitals and clinics)
- [ ] Fresno - UCSF Fresno OR Community Medical Center (CMC)
- [ ] Gladstone Institutes
- [ ] Institute on Aging (IOA)
- [ ] Jewish Home
- [ ] SF Dept of Public Health (DPH)
- [ ] SF VA Medical Center (SF VAMC)
- [ ] Vitalant (formerly Blood Centers of the Pacific and Blood Systems Research Institute)
- [ ] Zuckerberg San Francisco General (ZSFG)

6.2 LOCATIONS: At what locations will study visits and activities occur:

UCSF Benioff Children's Hospital Oakland, Claremont Clinic at 5220 Claremont Ave, Oakland, CA 94618

6.3 OFF-SITE PROCEDURES: Will any study procedures or tests be conducted off-site by non-UCSF personnel:

○ Yes ◉ No

6.4 RESEARCH PROGRAMS: Check any UCSF research programs this study is associated with:

- [ ] Cancer Center
- [ ] Center for AIDS Prevention Sciences (CAPS)
- [ ] Global Health Sciences
- [ ] Immune Tolerance Network (ITN)
- [ ] Neurosciences Clinical Research Unit (NCRU)
- [ ] Osher Center
- [ ] Positive Health Program

6.5 * CTSI CRS SERVICES: (REQUIRED) Will this study be carried out at one of the UCSF Clinical Research Services (CRS) units or utilize CRS services:

○ Yes ◉ No

6.6 * MULTI-CENTER TRIAL: (REQUIRED) Is this a multi-center or multi-site research trial:

By '**multi-center trial**' we mean a study where the protocol is developed by an lead investigator, an industry sponsor, consortium, a disease-group, etc.,and multiple sites across the nation or in different countries participate in the trial. The local sites do not have any control over the design of the protocol.

○ Yes ◉ No

6.8 OTHER SITE TYPES: Check all the other types of sites not affiliated with UCSF with which you are cooperating or collaborating on this project:

Do NOT check any boxes below if this is a multi-center clinical trial, UCSF is just one of the sites, and neither UCSF nor one of its faculty-linked affiliates (SF VAMC, Gladstone, ZSFG) are the coordinating center.

☐ Other UC Campus

☐ Other institution

☐ Other community-based site

☐ Foreign Country

☐ Sovereign Native American nation (e.g. Navajo Nation, Oglala Sioux Tribe, Havasupai, etc.)

6.14 * RELYING ON AN EXTERNAL IRB: (REQUIRED) Does this application include a request to rely on an external IRB (a central IRB (other than the NCI CIRB) or an external IRB (other UC campus, commercial, or institutional):

○ Yes ◉ No

7.0 Research Plan and Procedures

7.1 HYPOTHESIS: Describe the hypothesis or what the study hopes to prove:

Children with higher Adverse Childhood Events (ACE) scores will on average have worse health symptoms and indicators such as, more frequent infections, poorly controlled asthma and behavioral difficulties.
ACEs disclosure rates and strength of associations with key outcomes will vary based on different ACEs screening formats.
Higher ACE scores will be associated with various biomarkers of stress physiology including immune function and the neuroendocrine system.
Primary Care-based preventive interventions for toxic stress will be positively associated with changes in biomarkers, self-regulation, health and well-being.

7.2 AIMS: List the specific aims:

SPECIFIC AIMS

- **To validate the ACEs screening tool by (a) assessing the validity of the tool's association with physical and behavioral health indicators and biomarkers of stress, (b) determining the internal consistency of the tool, and (c) assessing the feasibility of universal implementation of the screening tool in a pediatric health care setting.**
- **Examine the relationships between ACEs, stress biomarkers, and symptoms in children and caregivers over time.**
- **Test whether providing primary care-based preventive interventions for children at risk of toxic stress can lead to change in biomarkers, self-regulation, behavioral and psychological outcomes for children and caregivers.**

7.3 DESIGN: Briefly describe the study design (e.g., observational, interventional, randomized, placebo-controlled, blinded, cross-over, cross-sectional, longitudinal, pharmacokinetic, etc.):

The PEARL study uses an experimental design to (a) validate the modified ACEs Screening Tool and (b) assess the impact of Anticipatory Guidance plus Preventive Interventions on biomarkers, physical health and mental health. The study utilizes a sample of children and caregivers receiving health care services at the UCSFBCHO Primary Care Clinic. Participants will be recruited from the population of children receiving well-child clinic visits, and randomized at two time points. (See Appendix for BARC ACEs Study Design).

The first randomization (1) will take place just after baseline data collection, into one of three ACEs Screen groups (No Screen, Identified Screen, De-Identified Screen), with the No Screen group proceeding to standard usual Care, and the Identified/De-Identified Screen groups proceeding to Anticipatory Guidance. The second randomization is for study subjects who have an ACES =>1, taking place a month after Anticipatory Guidance at T2, into one of two Preventive Intervention groups (Care Coordination or Resiliency Clinic). Baseline (T1) and 12-month follow-up (T4) data (biomarkers, health, child mental

/behavioral health) will be collected on all participants. At T2 and T3 data will be collected on measures of self-regulation, co-regulation, mental/behavioral health, and understanding of toxic stress.

Participants will be asked to provide a blood, and nasal swab sample. Study questionnaires and psychological measures will be administered; clinical measurements including heart rate, blood pressure, height, weight, and waist-hip circumference will be obtained; and, specimens (blood, oral, and nasal samples) will be collected on-site and sent to UCSF for processing and storage. All serologic assays, plasma /DNA/RNA extraction, genetic and biomarker testing will take place at UCSF in the Adversity BioCore (ABC) Bank (PI: Thakur).

Our goal is to obtain blood, and nasal swab samples for measurement of serum IgE, fibrinogen, CBC w/ differentials, and for DNA/RNA extraction for analysis of telomere length, candidate genes, and social and environmental risk factors for specific health outcomes including atopic diseases (asthma, rhinitis, eczema), acute infections (upper respiratory infections, otitis media, conjunctivitis, and urinary tract infections), developmental and behavioral outcomes in the presence of adversities as measured by the ACE score.

In addition to the above measurement, biomarkers of the inflammatory response (assorted cytokines) and of the neuroendocrine response (assorted lipid and hormonal measures) will be measured on blood specimens. Lastly, to examine nasal microbiome, we will deep sequence the 16s rRNA gene from DNA from nasal and oral samples of participants.

7.4 BACKGROUND AND SIGNIFICANCE: Briefly provide the background and significance of this study (e.g. why is this study needed) (space limit: one half page):

If this is a first in humans study, please summarize the safety data from the animal studies. For pediatric drug or device studies, please identify if this is the first study in pediatric populations.

Adverse Childhood Experiences (ACES) are stressful or traumatic events experienced in childhood that have been associated with poor health outcomes that extend into adulthood. The term “ACEs” was coined in 1998 following the publication of the Adverse Childhood Experiences Study (ACE study), which found that ACEs were common within the population, were associated with negative health outcomes in adulthood, and these associations occurred in a dose-response relationship (Felitti et al., 1998). Despite research showing a clear link between childhood adversity and poor health outcomes in adulthood, the pediatric medical community does not have a validated screening tool to identify children at risk. In addition, there are limited evidence-based interventions for the physiological consequences of toxic stress. Traditionally, interventions for traumatic stress have been mental health focused and little is known about whether these same interventions can effectively prevent and address the lifelong effects of toxic stress on physical disease. Lastly, while the concept of traumatic, or “toxic,” stress has been used to imply a mechanistic link between adversity, stress physiology and future poor health outcomes, the underlying mechanisms by which trauma leads to poor health are not well understood. This study begins to address these gaps. Using the medical model of identify, evaluate, educate and treat, this project will:

- Validate a pediatric ACE screening tool to identify children exposed to adversity,
- Provide anticipatory guidance to all families to further evaluate risk and educate caregivers about ACEs and toxic stress,
- Offer one of two clinic-based preventive interventions:
- care coordination to ensure that families get connected with the specific ACES-related services, or
- resiliency clinic that will target toxic stress physiology by teaching children and caregivers mindful self-regulation,
- Collect bio-specimens on all patients to begin to make correlations between ACEs, stress physiology and health outcomes.

Since the ACEs study, on-going research has supported similar findings in studies of children and adolescents. One study reported that over 90% of urban adolescent girls endorsed having at least one adverse experience, and 85% had specifically witnessed violence in their community (Lipschitz, 2000). Another study found that 67% of youth (mean age of 8) screened positive for at least one Adverse Childhood Experience (ACE). (Burke, 2011). ACEs in children have been correlated with fair or poor general health (Bethell, Newacheck, Hawes, & Halfon, 2014; Flaherty et al., 2013), illness requiring a doctor (Flaherty et al., 2013), fair or poor dental health (Bright, Alford, Hinojosa, Knapp, & Fernandez-

Baca, 2015), lifetime asthma risk (Bethell et al., 2014; Wing, Gjelsvik, Nocera, & McQuaid, 2015), ADHD (Bethell et al., 2014), autism (Bethell et al., 2014), and being overweight or obese (Bethell et al., 2014; Burke, Hellman, Scott, Weems, & Carrion, 2011). In addition, studies on ACEs during childhood and adolescence have found an association between ACEs and violent behavior (delinquent behavior, bullying, physical fighting, dating violence, weapon-carrying) (Duke, Pettingell, McMorris, & Borowsky, 2010) and learning difficulties (Burke et al., 2011).

Given these health outcomes associated with childhood adversity, the American Academy of Pediatrics (AAP) policy statement calls on pediatricians to actively screen for precipitants of toxic stress(Garner et al., 2012). Pediatric providers offer a unique opportunity for identifying and ameliorating toxic stress. Pediatricians see children at regular intervals, are trained to provide anticipatory guidance to prevent and educate families about a wide variety of public health issues, and understand the important role of parents and communities in determining a child's well-being (Garner et al., 2012). Yet, there is no validated, prospective, age-appropriate ACEs screening tool. The first arm of this study aims to validate a pediatric ACEs screening tool. The creation of a scientifically validated, standard screening tool is a crucial step that will facilitate widespread screening for ACEs in clinical pediatric settings.

Screening has limited utility in the absence of effective long-term interventions, and to date there are limited evidence-based interventions for the physiological and physical health consequences of toxic stress. This study will provide all families with ACEs-specific anticipatory guidance. Families with one or more ACEs will then get randomized to one of two interventions: care-coordination or the resiliency clinic. Both of these interventions will specifically address ACEs-related issues, and will be evaluated based on mental and physical health outcomes as well as biomarker profiles before and after the intervention.

Care coordination will be based on the Family Information & Navigation Desk (FIND) Program model. The overarching goal of care-coordination is to routinely identify a family's unmet basic social needs, in this case based on a modified ACE screener, and then connect the family to appropriate community resources. This model moves beyond a focus on biomedical and risk-behavior explanations of health to understand the root causes of health inequities and provides a preventive approach to population health. The goal is to target the social and environmental factors that profoundly impact health. A recent study of the FIND program found that families who had received care-coordination with appropriate referrals had significantly decreased social needs four months after starting the program (Gottlieb, 2016). More importantly, caregivers reported significantly improved children's overall health status four months later (Gottlieb, 2016).

The resiliency clinic is a monthly, mindfulness-based, caregiver-child group intervention focused on understanding toxic stress, and the development of self-regulation and co-regulation skills in caregivers and children identified as exposed to ACEs. The curriculum is based on existing models of mindfulness intervention (Kabat-Zinn, 2005; Liehr & Diaz, 2010; Gilkerson & Gray, 2014). The structure of the caregiver-child group will be based upon 30 years of experience at UCSF Benioff Children's Hospital Oakland's Early Intervention Services, and group collaboration from pediatric medical providers, developmental and behavioral specialists, and mental health specialists. While there are studies evaluating the buffering effect of a supportive caregiver (Dozier, Peloso, Lewis, Laurenceau, & Levine, 2008; Shonkoff et al., 2012; Scheering & Zeanah, 2001), the physical and mental health benefits of mindfulness-based interventions (Black, Semple Pokhrel & Grenard, 2011; Hölzel et al., 2011;Chambers, Gullone, & Allen, 2009; Burg, Wolf, & Michalak, 2012; Linehan, 1993; Roemer, 2015; Slopen, McLaughlin, & Shonkoff, 2014), the impact of mindfulness and self-awareness training for providers (Erikson on Children, 2015; Gilkerson et al., 2016), the effectiveness of mindfulness-based interventions in adult Primary Care (Demarzo, Montero-Marin, Cuijpers, et al., 2015), the health benefits of group pediatric visits targeted to specific medical conditions such as diabetes (Edelman, Gierisch, McDuffie, Oddone, & Williams, 2015; Housden, Wong & Dawes, 2013; Wall-Haas, Kulbok, Kirchgessner, & Rovnyak, 2012) and the power of group mental health interventions (Jones, Hutchings, Bywater, & Earmes, 2007; Meezan & O'Keefe, 1998; Gross, Fogg, & Tucker, 1995; Fristad, Goldberg-Arnold, & Gavazzi, 2003; Frame, Conley, & Berrick, 2006), to our knowledge an intervention that builds on the strengths of all of these isolated interventions has not been tested in a pediatric primary care setting.

The entire research project will be anchored by the collection of bio-specimens on each child. According to the Life Course Health Development Model, "health is a consequence of multiple determinants operating in nested genetic, biological, behavioral, social, and economic contexts that change as a person develops" (Halfon, 2002) and chaos (defined as crowding, unpredictability, etc.) of the surrounding environment leads to deleterious health effects (Kamp 2013). The normal stress response to acute life events induces several physiological responses that aid in adaptation and survival. Chronic exposure to adversity may result in a disruption of these normal stress pathways and is known as the toxic stress response (TSR). Several pathways have been suggested and include inflammatory and non-inflammatory mechanisms (disrupted neuroendocrine and/or autonomic nervous system functioning), epigenetic modification, and

alteration of the body's microbiome. These pathways are sensitive to individual differences and adaptation mechanisms and aberrations in any of these pathways may negatively affect disease outcomes. An increased understanding of the multiple pathways of stress may unveil causal mechanisms that can lead to novel clinical interventions and allow for better targeting of these interventions. The biomarkers selected for this proposal are representative of the hypothesized pathways to disease and were selected based on relevance, clinical availability, and novelty (Seeman, 1997; Seeman 2001; Karlamangla, 2002).

The collection of bio-specimens on all patients enrolled in the study will deepen our understanding of the correlations between ACEs, stress physiology and health outcomes. It will ground all aspects of this project in the underlying bio-chemical and genetic links between adversity and health outcomes. Biomarkers may help evaluate the concurrent validity of the pediatric ACEs screening tool and may offer insight on the pathways to poor health outcomes as a result of exposure to ACEs. Evaluating biomarker patterns and health outcomes in relation to ACE scores may allow for the establishment of a meaningful threshold for a clinical cut-point to the ACE score. Measuring biomarkers and therefore stress physiology before and after interventions may lead to more targeted and appropriate referrals and treatment modalities.

Ultimately, this study will help advance ACE screening in the pediatric clinic setting, offer families and providers further direction in choosing ACEs-related interventions, provide insight into the underlying biochemical patterns associated with adverse childhood experiences, and lay a strong foundation for future work elucidating the specific underlying mechanisms causing childhood adversity to lead to poor mental and physical health outcomes.

7.5 PRELIMINARY STUDIES: Briefly summarize any preliminary studies relevant to your proposed research (space limit: one half page):

The term "ACEs" was coined in 1998 following the publication of the Adverse Childhood Experiences Study (ACE study), which found that ACEs were common within the population, were associated with negative health outcomes in adulthood, and these associations occurred in a dose-response relationship (Felitti et al., 1998).

Since the ACEs study, on-going research has supported similar findings in studies of children and adolescents. One study reported that over 90% of urban adolescent girls endorsed having at least one adverse experience, and 85% had specifically witnessed violence in their community (Lipschitz, 2000). Another study found that 67% of youth (mean age of 8) screened positive for at least one Adverse Childhood Experience (ACE). (Burke, 2011). ACEs in children have been correlated with fair or poor general health (Bethell, Newacheck, Hawes, & Halfon, 2014; Flaherty et al., 2013), illness requiring a doctor (Flaherty et al., 2013), fair or poor dental health (Bright, Alford, Hinojosa, Knapp, & Fernandez-Baca, 2015), lifetime asthma risk (Bethell et al., 2014; Wing, Gjelsvik, Nocera, & McQuaid, 2015), ADHD (Bethell et al., 2014), autism (Bethell et al., 2014), and being overweight or obese (Bethell et al., 2014; Burke, Hellman, Scott, Weems, & Carrion, 2011). In addition, studies on ACEs during childhood and adolescence have found an association between ACEs and violent behavior (delinquent behavior, bullying, physical fighting, dating violence, weapon-carrying) (Duke, Pettingell, McMorris, & Borowsky, 2010) and learning difficulties (Burke et al., 2011).

7.6 * TREATMENT PROTOCOL: Is this a treatment study, i.e. does this study intend to provide treatment to individuals with a medical or psychological condition: (REQUIRED)

○ Yes ◉ No

7.7 * BILLABLE PROCEDURES: Does this study involve any procedures, lab tests or imaging studies that have a CPT code and could be billable to patients, their insurance, Medi-Cal, Medicare, or any other entity (answer 'Yes' even if the study is going to pay for all the procedures): (REQUIRED)

○ Yes ◉ No

If you are not sure if your study involves billable procedures, send an email to the UCSF Office of Clinical Research (OCR) for help answering this question.

7.8 * COMMON RESEARCH ACTIVITIES: Types of research activities that will be carried out. Check all that apply and describe in more detail in the 'Procedures / Methods' section: (REQUIRED)

- [x] Interviews, questionnaires, surveys
- [ ] Educational or cognitive tests
- [ ] Focus groups
- [ ] Social media-based research activities
- [ ] Observation
- [ ] Fitness tests or other exertion activities
- [ ] Use of mobile health apps or other apps
- [ ] Collection of data from wearable tech such as Fitbit, Apple Watch, Garmin, motion actigraphs, etc.)
- [ ] Non-invasive imaging or testing (MRI, EEG, pulse oximetry, etc.)
- [ ] Imaging procedures or treatment procedures that involve radiation (x-rays, CT scans, CT-guided biopsies, DEXA scans, MUGA or PET scan)
- [ ] Administration of contrast agent
- [x] Randomization to one intervention versus another
- [ ] Use of placebo
- [ ] Biopsy conducted solely for research purposes
- [ ] Sham surgical procedure
- [ ] None of the above

7.9 * PROCEDURES / METHODS: (REQUIRED)

Describe the research methods and study activities taking place at each site (e.g. what will participants be asked to do and what will members of the study team do?). If there will be multiple participant groups or study sites, explain what will happen with each group or study sites.

If some of the activities would occur even if the person were not in the study, as in the case of treatment or tests performed for diagnostic purposes,
clearly differentiate between those activities that will be done solely for research purposes and those that are happening as part of routine care.

Please call our office at 415-476-1814 and ask to speak to someone on the Expedited Review team if you need help differentiating between what parts are research and what parts aren't.

Study procedures are complete. Enrollment is closed. Data analysis only at time of transfer.

See Appendix for Study Design and Flow

T1 Baseline Data Collection

1. Once eligibility is confirmed and informed consent has been obtained, Study Coordinator will obtain vital signs from child: Blood pressure, resting heart rate, waist and hip circumference, height and weight (see Appendix for Age-Appropriate Biomarker and Specimen Collection Protocol)
2. Medical provider is informed of patient/caregiver study participation and need for extra time to complete forms prior to well-child visit.
3. The Study Coordinator will log into a web-enabled REDCap database and enter the child and caregiver names, dates of birth and race/ethnicities, generating one subject ID each for the child and caregiver. The subject ID's will then be entered into a separate REDCap randomization system, and the Study Coordinator will print out the subjects' random assignment #1 and insert this designation into the study folder.
4. While roomed, caregiver is asked to complete the following forms and measures on an electronic tablet (see Appendix for copies) in the following order. (If necessary due to clinic flow and availability of medical provider, the items marked * below can be completed after biomarker collection). Study Coordinator will offer to read questions to caregiver aloud, or to have caregiver complete forms on their own, or a combination. Paper copies will also be made available as an alternative to the electronic tablet.

1. Demographic Form
2. Health Form
3. PROMIS Scale

If child is randomized to either the Identified/De-Identified Screen Group:

1.
    1. Modified Child ACES Screen(Depending upon random assignment #1) appropriate ACEs Screen (i.e., Identified/De-Identified or NO Screen). The "Identified" ACEs Screen lists each adverse experience and displays, for the caregiver and the provider, each item endorsed on the screen. The "De-Identified" ACEs Screen lists each adverse experience and displays on the screen, for the caregiver and provider, ONLY the total number of adverse experiences although the specific items endorsed are entered into the research database. If child /caregiver is randomized to either Identified/De-Identified Screen group:
    2. A study coordinator gives completed Modified Child ACEs screen (Identified or De-Identified) to medical provider, who reviews.
    3. A medical provider completes well-child check and offers anticipatory guidance to ALL caregiver/child regarding ACEs and toxic stress
    4. After the provider offers anticipatory guidance, the study mental health clinician will briefly ask the caregiver their thoughts about the Modified ACES screener. These questions will include elicititng the caregiver opinion about the way in which they answered the questions as an idenitified screen or de-identified.
    5. If result of screen is ACEs=>1, medical provider lets caregiver know that after biomarker collection, they will meet briefly with mental health clinician to debrief, including an opportunity to discuss specific ACEs (whether they completed an "Identified" or "De-Identified" Screen) if they wish to do so.
    6. <u>If result of screen is ACEs=0,</u>the well-child check is followed by study coordinator completing biomarker collection. They move into the usual care group.
    7. All Caregiver/child meets with mental health clinician, who debriefs about well-child check, ACEs screen and other measures, and anticipatory guidance from medical provider.

<u>Biomarker Collection:</u>

Summary: Participants will be asked to provide a blood,, and nasal swab sample for genetic and serologic testing of genetic and biologic markers related to adversity and the specified health outcomes. At the moment there are no identifiable chemicals or 'Toxic Stress' gene. It is unlikely that a specific toxic stress chemical or gene will ever be identified, because toxic stress is considered to be the result of genetic and environmental risk factors.

All participants will be asked if their cells may be Cryopreserved (frozen) for future cell immortalization. All participants will be asked if they would be interested in being re-contacted for future studies.

Heel Stick for Dried Blood Spot Collection: Participants that are less than 1 year (12 months) of age, a heel stick will be performed to collect 3-5 dried blood spots on filter paper. This amounts to 375-500 microliters of blood per participant. This procedure is similar to newborn screening test that occurs at birth. Specimens will be stored and later DNA/RNA and protein biomarkers related to stress will be extracted from the samples and analyzed for the presence of genes and biomarkers thought possibly to be related to stress.

Venipuncture for Blood Sample: Venipuncture is performed with a needle and syringe to draw approximately of 7 ml (participant's 1-5 years old) to 11.5 ml (participants > 5 years old) of blood from a vein in the arm. DNA/RNA and plasma will be extracted from the samples and analyzed later for the presence of genes and biomarkers thought possibly to be related to stress.

Collection of Microbiome and DNA using oral and nasal swabs: Buccal mucosal and nasal swabs will be collected from all participants using the MoBio collection tubes. The DNA will be extracted from the samples and later analyzed for the presence of genes and chemicals thought possibly to be related to adversity. The use of human bacteria will be used to determine whether bacteria of specific types of bacteria are associated with adversity.

Measurement of Genetic Ancestry: Although we will use a questionnaire to assess racial and ethnic background, among racially admixed populations such as Latinos or African Americans, most participants will not know their true racial background or racial admixture proportions. Genetic admixture can be measured and adjusted for just like any other quantitative variable. There is evidence that racial/ethnic groups have varied physiological responses to stress. Measuring Genetic Ancestry will allow for better identification of genes or proteins associated with these varies responses. This will enable future studies of adversity through an approach presently known as admixture mapping. Information about racial ancestry will not be shared with study participants.

Freeze Cells (cryopreservation): Cells will be frozen which someday may be used to analyze the type of cells and what types of genes are turned on or off (gene expression).

Cell immortalization: In addition to saving the left over samples, in some instances, the researchers may "immortalize" the cells collected from the child. This will provide additional DNA which can then be studied in future research. Cell immortalization means that the cells may be changed in a laboratory so that they will grow and divide continuously. Immortalized cells are an important resource, and if cells from thechild's sample are changed in this way, they may be used by researchers for many years in the future to develop new drugs, tests, treatments or products. In some cases these may have potential commercial value. There are no plans to share future profits with the caregiver and child. See Appendix for Age-Appropriate Biomarker and Specimen Collection Protocol.

After completing biomarker collection, the study coordinator will:
1. Enter Study ID into randomization database for random assignment #2
2. File forms, measures & randomization #2 in study folder
3. Scheduling T2 visit 2 weeks after T1

<u>If caregiver/child is randomized to NO ACEs Screen group</u>:

1. Medical provider completes well-child check.
2. Well-child check is followed by Study Coordinator completing biomarker collection.
3. Study Coordinator schedules caregiver/child to return for T2 data visit visit within 2 weeks of T1.
Participants will be contacted by email, text, mail and/or phone to remind of upcoming T2 data collection visit and offer to answer any questions or address concerns.

<u>T2 Data Collection Visit</u>

1. This visit will be video taped. Study Coordinator turns on video camera in exam room. Child and caregiver are roomed for at least 10 minutes, with videotaped child-caregiver interaction during vital signs (below) and any wait time in exam room.
2. Study Coordinator will obtain vital signs from child: Blood pressure, resting heart rate, waist and hip circumference, height and weight.
3. After approximately 10 minutes, video camera is turned off.
4. Caregiver and child are offered the option to have study staff/hospital volunteer play nearby with child for 5 minutes while caregiver completes the STROOP Color and Word Test (administered to the caregiver by the Study Coordinator)
5. The caregiver will be offered the choice to complete the following measures on their own, or for the Study Coordinator to administer them as brief interviews in their preferred language. Paper copies will also be made available as an alternative to the electronic tablet.

- Behavior Rating Inventory of Executive Function (BRIEF-P or BRIEF-2)
- Perceived Stress Scale (PSS)
- Brief Symptom Inventory-18 (BSI-18)
- Reflecting on Stress and Coping Questionnaire (RSCQ)
- Short Self-Regulation Questionnaire (SSRQ)
- Devereaux Early Childhood Assessment Scale (DECA-Infant/DECA-Toddler/DECA-Preschool)
- Child Behavior Checklist (CBCL 1.5-5 or 6-18)
- Adult ACES Screen
- Assessments for Asthma, Eczema, Rhinitis, Sleep and Social Needs
- Subjective Status Ladder Screener

Estimated time to completion of these 7 caregiver measures is approximately 60-75 minutes total.

While the caregiver is completing the above forms, if the child is age 2-11 years old, the Study Coordinator will administer the Minnesota Executive Function S to the child on an This measure takes approximately 4-6 minutes to complete with the child.

In a randomly selected subset of the total sample (approximately 20%), caregivers will also be asked to complete a second modified child ACEs screen. The purpose of this is for the examination of test-retest reliability.

Total estimated time for T2 visit (vitals, caregiver completed measures, and one child completed measure) is 90 – 120 minutes.

After measures are complete for the caregivers/child dyad who was randomized to the Modified Child ACES screen group and who has a child ACES score >1, the Study Coordinator describes the next randomization to the interventions (Care Coordination/Resiliency Clinic) and answers questions. Study Coordinator schedules first intervention appointment (either Care Coordination or Resiliency Clinic orientation) within 2-4 weeks of T1. Study Coordinator schedules caregiver/child to return for T3 visit within 2 weeks of last intervention appointment (approximately 7-7.5 months following consent). Participants will be contacted by email, text, mail and/or phone to remind of upcoming T3 data collection visit and offer to answer any questions or address concerns.

Following the T2 data collection visit, each group proceeds as follows:

1. Usual Care 1(No ACEs Screen): receives care as usual in Primary Care.
2. Usual Care 2(ACEs=0): receives care as usual in Primary Care.
3. Care Coordination: Experimental Intervention. Caregiver-child dyads will be offered 6 monthly appointments with a Care Coordinator over a 6 month period, each lasting up to 2 hours. The Care Coordination intervention is based upon the previously tested Family Information Navigation Desk (FIND) program in Primary Care at Children's, which screens for basic social needs and connects families to resources. Medical providers write and give prescriptions for Care Coordination services to caregivers. Trained staff and volunteers screen families for social issues affecting their health, including tobacco exposure, as well as for basic social needs such as access to nutrition, housing, and utilities. The Care Coordination intervention will enhance FIND by adding the capacity to provide mental health referrals and connect to supportive resources. (See Appendix for Care Coordination description).
4. Resiliency Clinic: Experimental Intervention. Caregiver-child dyads will participate in 6 monthly Resiliency Clinic visits over a 6 month period, each lasting a total of 2 hours. During the clinic, six caregiver/child dyads will gather in a large room in Primary Care, where a team of facilitators (medical provider, mental health clinician, developmental specialist) will address health and wellness concerns about the child, including a review of sleep, nutrition, exercise, healthy relationships and mental health, and participate in a 1 hour 20 minute child-caregiver group designed to build and practice skills in mindful self-regulation. The pedagogical methodology used will incorporate deliberately scaffolded activities in which participants are:(a) supported in rallying around a common goal (e.g., reducing the associated effects of stress), (b) participating in naming their present experience (learning about states and how to negotiate them), (c) receiving dynamic and adaptive support "real time" to utilize new skills (mindfulness-based practice), (d) engaging in age appropriate dialogues and interactions (mindful movement, music, and other presence based activities), (e) all with a planned transfer of responsibility to families, as part of the program. (See Appendix - Resiliency Clinic Pilot Sample Curriculum)

Caregivers will be asked at consent to enroll in MyChart and provide their email address, in order to be contacted between visits. Study staff will then communicate with caregivers through EPIC MyChart email. Communications will consist of checking in with caregivers, delivering weekly mini-doses of content related to mindfulness and toxic stress, and reminding them of upcoming clinic visits.

T3 Data Collection Visit

1. Study Coordinator turns on video camera in exam room. Child and caregiver are roomed for at least 10 minutes, with videotaped child-caregiver interaction during vital signs (below) and any wait time in exam room.
2. Study Coordinator will obtain vital signs from child: Blood pressure, resting heart rate, waist and hip circumference, height and weight.
3. After approximately 10 minutes, video camera is turned off.
4. Caregiver and child are offered the option to have study staff/hospital volunteer play nearby with child for 5 minutes while caregiver completes the STROOP Color and Word Test (administered to the caregiver by the Study Coordinator)
5. The caregiver will be offered the choice to complete the following measures on their own, or for the Study Coordinator to administer them as brief interviews in their preferred language. Paper copies will also be made available as an alternative to the electronic tablet.

- the Behavior Rating Inventory of Executive Function (BRIEF-P or BRIEF)
- the Perceived Stress Scale (PSS)
- the Brief Symptom Inventory-18 (BSI-18)
- the Reflecting on Stress and Coping Questionnaire (RSCQ)
- the Short Self-Regulation Questionnaire (SSRQ)
- the Devereaux Early Childhood Assessment Scale (DECA-Infant/DECA-Toddler/DECA-Preschool)
- Caregiver Follow up Interview.

Estimated time to completion of these 8 caregiver measures is approximately 75-90 minutes total. While

the caregiver is completing the above forms, if the child is age 2-11 years old, the Study Coordinator will administer the following to the child on an Ipad the Minnesota Executive Function Scale.

In a randomly selected subset of the total sample different from the subset identified at T2 (approximately 20%), caregivers will also be asked to complete a second modified child ACEs screen. The purpose of this is for the examination of test-retest reliability.

Total estimated time for visit (vitals, caregiver completed measures, and one child completed measure is 90 – 120 minutes. Study Coordinator schedules caregiver/child to return for T4 visit within 2 weeks of 12 months following date of consent). Participants will be contacted by email, text, mail and/or phone to remind of upcoming T4 data collection visit and offer to answer any questions or address concerns.

T4 Data Collection Visit

1. Study Coordinator will obtain vital signs from child: Blood pressure, resting heart rate, waist and hip circumference, height and weight.
2. While roomed, caregiver is asked to complete the following forms and measures on an electronic tablet (see Appendix for copies) in the following order. Study Coordinator will offer to read questions to caregiver aloud, or to have caregiver complete forms on their own, or a combination. Paper copies will also be made available as an alternative to the electronic tablet.
3. Child Behavior Checklist (CBCL 1.5-5 or 6-18)
4. Caregiver ACEs Screen.
5. Modified child ACEs Screen (Identified; adapted to be retrospective including past 12 months)
6. Demographic form (if any updates apply i.e., household status, education, income)
7. Health form
8. Assessments for Asthma, Eczema, Rhinitis, Sleep and Social Needs
9. Subjective Status Ladder Screener
10. Study Coordinator will complete biomarker collection (see Heel Stick, Blood Draw, and Cheek and Nasal Swab instructions for T1 above; see also Appendix for Age-Appropriate Biomarker and Specimen Collection Protocol)

Electronic Medical Record

Participants will be asked and consented to provide their medical record number so that we may link relevant clinical data to the current study. Study staff will either request a report from the EHR system, or will perform a cursory chart review at study points T1, T2, T3, and T4 to record the participant's current medication list and medical problems, including those related to mental health, referrals and utilization data. We will also collect information regarding acute health events and associated antibiotic use that have occurred at the time of visit and the preceding 12 months, and up to 12 months following enrollment. Acute health events of interest include: upper respiratory infection, otitis media, conjunctivitis, and urinary tract infections.

7.11 INSTRUMENTS: List all questionnaires, surveys, interview, or focus group guides that will be used for this study:

If the instruments are not complete or not available because they will be developed as part of this study, describe the basic content or include an outline and submit the final versions to the IRB with a modification for approval prior to use.

Caregiver self-report measures include:

- the Modified Child ACEs Screen

- Caregiver ACEs screen

Behavioral and mental health screens including

--the BRIEF-2 or BRIEF-P

-- the PSS

-- the BSI-18

-- the RSCQ

-- the SSRQ

-- the STROOP

-- CBCL1.5-5 or CBCL 6-18

--the DECA-I or DECA-T or DECA-P2

Health Screens

--Assessments for Asthma, Eczema, Rhinitis, and Sleep

Social Determinants of Health Screens

--Social Needs Assessment

--Subjective Status Ladder Screener

Child (age 2 years and older) completed measure:

Minnesota Executive Function Scale

Each of these measures will be administered by a Study Coordinator, who will offer the caregiver the option to complete the scale independently by reading it to themselves, or to have the Study Coordinator administer it as an interview in their preferred language. If the scale is completed independently the Study Coordinator will be available to answer clarifying questions and insure completeness.

These measures will be completed in the exam room.

The BRIEF-2/BRIEF-P, DECA-I/T/P and the BSI each require Level B qualification for purchase. This may be accomplished by having obtained a degree from an accredited 4-year college or university in psychology, counseling, speech-language pathology, or a closely related field plus satisfactory completion of coursework in test interpretation, psychometrics and measurement theory, educational statistics, or a closely related area; or license or certification from an agency that requires appropriate training and experience in the ethical and competent use of psychological tests. Additionally, certain health care providers may be eligible to purchase selected Level B and/or C instruments within their area of expertise. Specifically, relevant supervised clinical experience using tests (i.e., internship, residency) in combination with formal coursework (i.e., tests and measurement, individual assessment, or equivalent) qualifies a health care provider to purchase certain restricted products. Dr. Frame meetsthese qualifications, and will train and supervise Study Coordinators to administer the measures.

The STROOP requires a level C mental health qualification to purchase the measure, which is met by Dr. Frame. This qualification includes level B requirements and an advanced professional degree (with training in psychological testing) or license/certification from an agency that requires training in the use of psychological test.

The Minnesota Executive Function Scale will be administered by the Study Coordinator to the child on an Ipad, in the exam room. The caregiver may be present and will be seated behind the child to avoid interference with the child’s responses. The MEFS requires official training and certification by Reflection Sciences, to be qualified to administer.

The ACEs Screen, Modified child ACEs Screen, PSS, CBCL (1.5-5 and 6-18), and the Reflecting on Stress and Coping Questionnaire do not require special qualifications to administer, and the PI will provide training.

Attach any unpublished instruments in the 'Other Study Documents' section of the Initial Review Submission Packet form after completing the study application. Published instruments should NOT be attached.

7.12 * BIOSPECIMEN COLLECTION: Are you drawing any blood or collecting other biosamples (e.g. tissue, buccal swabs, urine, saliva, hair, etc.) for analysis under this protocol and/or storage for future research: (REQUIRED)

(●) Yes ( ) No

* Could this study generate genetic data that may be broadly shared (e.g., submitted to NIH in compliance with **Genomic Data Sharing (GDS)/Genome-Wide Association Studies (GWAS)** requirements): **(REQUIRED)**

(●) Yes ( ) No

Please make sure your consent form includes the recommended genomic data sharing language.

7.13 STATISTICAL METHODS: Briefly summarize the methods and types of analyses that will be performed:

All data will be merged and undergo final cleaning by the Consortium's Data Core at UC San Francisco using SAS. Our data analytic plans directly follows from our specific aims.

Sample Size/Power: Sample size and power estimates for comparison of the two ACEs formats in Phase 3 are based on alpha = 0.05 and 2-sided t-tests. Estimates of associations between ACE items with child biomarkers, and behavioral health outcomes in similar studies suggest effect sizes that are small to moderate, with standardized effects ranging from d=0.30 to 0.48. [1,2,3,4] Effect sizes for associations between ACEs or early psychosocial exposures with health diagnoses in related adult and child literatures similarly suggest small to moderate effect sizes of OR=1.6-1.9 across a range of diagnoses (e.g., acute upper respiratory infections, otitis media, viral infections, eczema, UTI, and asthma). [5,6] We intend to obtain a sample of n=555 (n=185 per arm). This sample size will provide power to exceed 0.80 for evaluation of the association between ACEs with the biomarkers, child behavioral health indicators, and child diagnoses. Within the Identified arm, the ratio of ACEs items to respondents exceeds 5.0 for factor analytic techniques.[7,8]For tests directly comparing between group differences across arms (identified vs. de-identified screening format), this sample size (n = 370 total, n=185 per arm) will additionally allow for detection of an approximate 11% difference in disclosure rates for a given number of ACEs [4] or standardized units mean difference in number of ACEs. At the projected sample size for each group, power to discriminate at this level between the two arms will be at least 80%. The total number of patients who came to CPCC for well visits in 2013 was 8,714. 12% were Spanish speakers. Of all patients, 4,760 were ages 0-3, 971 ages 4-5, and 2,160 ages 6-12. Given the large number of patients seen annually at CPCC, coupled with our previous success implementing the FIND study, we do not expect to have difficulty recruiting this sample size.

Descriptive Analyses & Missing Data: Descriptive analyses will include family demographics, ACEs (parent self-report and report of child), biomarkers, and assessments of children's symptoms, behavior, and physical health. In our analytic plan, we examine ACE scores by: a continuous total score, individual items, and with subscale factor scores and a cut-point score pending the results of the validation study analyses and where appropriate. Data will be inspected for outliers and out-of-range values. Examination of distributions may prompt transformations, where they are defensible and are a component of the best available analysis strategy. Following recommendations by Dube and colleagues, missing ACEs data will be conservatively considered not to have that experience. Likelihood based approaches (e.g. GLMMs) and multiple imputation will be used to handle missing data for other variables . Both approaches fit models to all available data and invoke the relative assumption that the data are missing at random. Background demographics will be examined to describe the sample and will be included in multivariate analysis if they are related to the outcome at $p<.2$, differ between treatment arms, or associated with dropout. Correlations among variables within each area will be examined and redundant measures will be combined or eliminated to avoid multicollinearity.

Specific Aim 1: Cross sectional and longitudinal associations between ACEs, biomarkers, health symptoms and mental/behavioral health indicators in children and parents: Cross sectional associations will be examined with linear mixed models that include intervention group as a predictor. Generalized linear mixed models (GLMMs) will examine the correlations between parent and child ACE scores, biomarkers, and symptoms across time . First, each measure will be modeled separately to determine whether it increased, decreased, or remained constant over time both within and across study arms. Once successfully modeled separately, they will be modeled in two ways: to examine the predictive effect of change in one measure on another over time and vice versa (unidirectional regression model), and to determine whether change in one measure and change in another significantly co-varied together over time (bidirectional regression model). For both models, intervention group will be included as a covariate to assess for differential effects by study arm, as well as key covariates identified in preliminary analyses.

Specific Aim 2: The Impact of Intervention on Child and Caregiver Outcomes: We will fit regression models for cross-sectional and longitudinal outcomes. Demographics will be compared for the usual care, and the intervention arms at baseline. Comparisons of the Usual Care and Intervention arms on changes in biomarkers will be estimated with linear mixed models and generalized linear models with generalized estimating equations (e.g., SAS PROC MIXED ROC GENMOD, SAS Institute Inc., 1999). Similar models will be conducted for additional outcomes including: caregiver understanding of toxic stress and the buffering role of parenting, as well as openness to help-seeking. Generally, we seek to model longitudinally the trajectory of these outcomes as a function of time, intervention group assignment, and group-by-time interaction. We begin by fitting base models, those with minimum covariates and, for longitudinal models, the most restricted residual covariance structures. Additional covariates then will be considered (including demographic variables, referrals and services). Empirical contributions to model selection decisions will include reference to information criteria (e.g., Akaike's). We will examine change from baseline for each outcome as the dependent variable. In addition to testing the groups-by-time interaction term, custom models and contrasts will assess group differences at each time point. Change from baseline to 6 week and 12 month follow up will allow for testing the short and long term effects of the brief targeted intervention. Primary tests will compare participants assigned to the intervention arm vs. participants assigned to usual care with an ACE score of ≥ 1. Participants assigned to the usual care arm with an ACE score of 0 will additionally be explored as a non-exposed control comparison group.

Moderating Analyses: These analyses will further elaborate any observed treatment effects. A first set will determine whether demographic strata, baseline ACE score, biomarkers, or intervention dose effects moderate any intervention effects. Each outcome will be regressed onto measures of intervention, baseline or other covariates and their interactions with intervention group assignment. In these models, a significant interaction term suggests that the intervention effect is moderated by the corresponding variable.

References

1. Evans, S. E., Davies, C., & DiLillo, D. (2008). Exposure to domestic violence: A meta-analysis of child

and adolescent outcomes. Aggression and Violent Behavior, 13(2), 131-140

2. Cicchetti, D., Rogosch, F. A., Gunnar, M. R., & Toth, S. L. (2010). The Differential Impacts of Early

Physical and Sexual Abuse and Internalizing Problems on Daytime Cortisol Rhythm in School--%u2010Aged

Children. Child development, 81(1), 252-269.

3. Finklehor D. 2013

4. Theall K, Drury S, Shirtcliff E. Cumulative Neighborhood Risk of Psychosocial Stress and Allostatic

Load in Adolescents. Am J Epidemiol. 2012;176(Suppl):S164–S174.

5. Felitti VJ, Anda RF, Nordenberg D, Williamson DF, Spitz AM, Edwards V, Koss MP, et al. The

relationship of adult health status to childhood abuse and household dysfunction. Amer J of Prev Med.

1998; 14:245:258

6. Karlen, J., Ludvigsson, J., Hedmark, M. Farsjo, A., Therodorsson, E., Faresjo, T. Early Psychosocial exposures, hair cortisol level and disease risk. Pediatrics. 2015. 135, e1450-57

7. Bryant FB and Tarnold PR. Principle components analysis and exploratory and confirmatory factor analysis. . In Reading and understanding multivariate statistics. Grimm LG and Tarnold RR Eds. Washington, D.C., American Psychological Association, 1995, p. 99-136.

8. Comrey AL and Lee HB. A first course in factor analysis. Hilsdale, N.J., Erlbaum, 1992

7.14 REFERENCES: List only the 5-10 most relevant references (a separate bibliography can be attached for reference purposes if this study involves novel approaches, agents, or an emerging technology that the IRB may not be familiar with):

References

1. Black, D. S., Semple, R. J., Pokhrel, P., & Grenard, J. L. (2011). Component Processes of Executive Function—Mindfulness, Self-control, and Working Memory—and Their Relationships with Mental and Behavioral Health. Mindfulness, 2(3), 179–185. http://doi.org/10.1007/s12671-011-0057-2

2. Bethell CD, Newacheck P, Hawes E, Halfon N. Adverse child- hood experiences: Assessing the impact on health and school engagement and the mitigating role of resilience. Health Aff (Millwood). 2014;33(12): 2106-2115. doi:10.1377/ hlthaff.2014.0914. %u2028

3. Bright MA, Alford SM, Hinojosa MS, Knapp C, Fernandez-Ba- ca DE. Adverse childhood experiences and dental health in children and adolescents. Community Dent Oral Epidemiol. 2015;43(3):193-199. doi: 10.1111/cdoe.12137. %u2028

4. Burke NJ, Hellman JL, Scott BG, Weems CF, Carrion VG. The impact of adverse childhood experiences on an urban pediatric population. Child Abuse Negl. 2011;35(6):408-413.

5. Burg, J. M., Wolf, O. T., & Johannes Michalak. (2012). Mindfulness as Self-Regulated Attention. Swiss Journal of Psychology, 71(3), 135–139. http://doi.org/10.1024/1421-0185/a000080

6. Chambers, R., Gullone, E., & Allen, N. B. (2009). Mindful emotion regulation: An integrative review. Clinical Psychology Review, 29(6), 560–572. http://doi.org/10.1016/j.cpr.2009.06.005

7. Demarzo, M. M. P., Montero-Marin, J., Cuijpers, P., Zabaleta-del-Olmo, E., Mahtani, K. R., Vellinga, A., … García-Campayo, J. (2015). The Efficacy of Mindfulness-Based Interventions in Primary Care: A Meta-Analytic Review. Annals of Family Medicine, 13(6), 573–582. http://doi.org/10.1370/afm.1863

8. Dozier, M., Peloso, E., Lewis, E., Laurenceau, J., & Levine, S. (2008). Effects of an attachment-based intervention on the cortisol production of infants and toddlers in foster care. Development and Psychopathology, 20(3), 845–59.

9. Duke NN, Pettingell SL, McMorris BJ, Borowsky IW. Ad- olescent violence perpetration: Associations with multiple types of adverse childhood experiences. Pediatrics. 2010;125(4):e778-e786. doi:10.1542/peds. 2009-0597. %u2028

10. Edelman, D., Gierisch, J. M., McDuffie, J. R., Oddone, E., & Williams, J. W. (2015). Shared Medical Appointments for Patients with Diabetes Mellitus: A Systematic Review. Journal of General Internal Medicine, 30(1), 99–106. http://doi.org/10.1007/s11606-014-2978-7

11. Erikson on Children. (2015). A FAN for all seasons. Spring 2015

12. Felitti, V. J., Anda, R. F., Nordenberg, D., Williamson, D. F., Spitz, A. M., Edwards, V., … Marks, J. S. (1998). Relationship of childhood abuse and household dysfunction to many of the leading causes of death in adults: The Adverse Childhood Experiences (ACE) Study. American Journal of Preventive Medicine, 14 (4), 245–258.

13. Flaherty EG, Thompson R, Dubowitz H, et al. Adverse child- hood experiences and child health in early adolescence. JAMA Pediatr. 2013;167(7):622-629. %u2028

14. Frame, L., Conley, A., & Berrick, J. D. (2006). “The Real Work is What They Do Together”: Peer Support and Birth Parent Change. Families in Society: The Journal of Contemporary Social Services, 87(4), 509–520. http://doi.org/10.1606/1044-3894.3566

15. Fristad, M. A., Goldberg-Arnold, J. S., & Gavazzi, S. M. (2003). Multi-family psycho-education groups in the treatment of children with mood disorders. Journal of Marital and Family Therapy, 29(4), 491–504.

16. Garner, A. S., Shonkoff, J. P., Siegel, B. S., Dobbins, M. I., Earls, M. F., Garner, A. S., … Wood, D. L. (2012). Early childhood adversity, toxic stress, and the role of the pediatrician: Translating developmental science into lifelong health. PEDIATRICS, 129(1), e224–e231. http://doi.org/10.1542/peds.2011-2662

17. Gilkerson, L., & Gray, L. (2014). Fussy babies: Early challenges in regulation, impact on the dyad and family, and longer-term implications. Alexandria, VA: In K. Brandt, B. Perry, S. Seligman, & E. Tronick (Eds.), Infant and Early Childhood Mental Health. American Psychiatric Publishing.

18. Gilkerson, L., Justice, R., Gray, L., Barnes, M., Osta, A., Pryce, J., & Wildman, A. (2016, January). Facilitating Attuned Interactions in Primary Care. Poster presented at the Erikson Institute, Chicago, IL.

19. Gottlieb LM, Hessler D, Long D, Laves E, Burns AR, Amaya A, Sweeney P, Schudel C, Adler NE. Effects of Social Needs Screening and In-Person Service Navigation on Child Health: A Randomized Clinical Trial. JAMA Pediatr. Published online September 06, 2016.

20. Gross, D., Fogg, L., & Tucker, S. (1995). The efficacy of parent training for promoting positive parent-toddler relationships. Research in Nursing & Health, 18(6), 489–499.

21. Halfon, N., & Hochstein, M. (2002). Life Course Health Development: An Integrated Framework for Developing Health, Policy, and Research. The Milbank Quarterly, 80(3), 433–479. http://doi.org/10.1111/1468-0009.00019

22. Hölzel, B. K., Lazar, S. W., Gard, T., Schuman-Olivier, Z., Vago, D. R., & Ott, U. (2011). How Does Mindfulness Meditation Work? Proposing Mechanisms of Action From a Conceptual and Neural Perspective. Perspectives on Psychological Science, 6(6), 537–559. http://doi.org/10.1177/1745691611419671

23. Housden, L., Wong, S. T., & Dawes, M. (2013). Effectiveness of group medical visits for improving diabetes care: a systematic review and meta-analysis. Canadian Medical Association Journal, cmaj.130053. http://doi.org/10.1503/cmaj.130053

24. Jones, K., Daley, D., Hutchings, J., Bywater, T., & Eames, C. (2007). Efficacy of the Incredible Years Basic parent training programme as an early intervention for children with conduct problems and ADHD. Child: Care, Health and Development, 33(6), 749–756. http://doi.org/10.1111/j.1365-2214.2007.00747.x

25. Kabat-Zinn, J. (2005). Full catastrophe living: Using the wisdom of your body and mind to face stress, pain, and illness: Fifteenth anniversary edition. New York: Bantam Dell.

26. Kamp Dush CM, Schmeer KK, Taylor M. Chaos as a social determinant of child health: Reciprocal associations? Soc Sci Med 2013.

27. Karlamangla AS, Singer BH, McEwen BS, Rowe JW, Seeman TE. Allostatic load as a predictor of functional decline. MacArthur studies of successful aging. J Clin Epidemiol 2002;55:696-710.

28. Liehr, P., & Diaz, N. (2010). A Pilot Study Examining the Effect of Mindfulness on Depression and Anxiety for Minority Children. Archives of Psychiatric Nursing, 24(1), 69–71. http://doi.org/10.1016/j.apnu.2009.10.001

29. Linehan, M. M. (1993). Skills training manual for treating borderline personality disorder (Vol. xii). New York, NY, US: Guilford Press.

30. Lipschitz DS, Rasmusson AM, Anyan W, Cromwell P, Southwick SM. (2000) Clinical and functional correlates of posttraumatic stress disorder in urban adolescent girls at a primary care clinic. J Am Acad Child Adolesc Psychiatry, 39 (9), 1104-1111.

31. Meezan, W., & O’Keefe, M. (1998). Evaluating the Effectiveness of Multifamily Group Therapy in Child Abuse and Neglect. Research on Social Work Practice, 8(3), 330–353. http://doi.org/10.1177/104973159800800306

32. Roemer, L., Williston, S. K., & Rollins, L. G. (2015). Mindfulness and emotion regulation. Current Opinion in Psychology, 3, 52–57. http://doi.org/10.1016/j.copsyc.2015.02.006

33. Scheering, M. S., & Zeanah, C. H. (2001). A relational perspective on PTSD in early childhood. Journal of Traumatic Stress, 14(4), 799–815. http://doi.org/10.1023/A:1013002507972

34. Seeman TE, Singer BH, Rowe JW, Horwitz RI, McEwen BS. Price of adaptation--allostatic load and its health consequences. MacArthur studies of successful aging. Arch Intern Med 1997;157:2259-68.%u2028

35. Seeman TE, McEwen BS, Rowe JW, Singer BH. Allostatic load as a marker of cumulative biological risk: MacArthur studies of successful aging. Proceedings of the National Academy of Sciences of the United States of America 2001;98:4770-5.%u2028

36. Shonkoff, J. P., Garner, A. S., Siegel, B. S., Dobbins, M. I., Earls, M. F., McGuinn, L., … The Committee on Psychosocial Aspects of Child and Family Health, Committee on Early Childhood, Adoption, and Dependent Care, and Section on Developmental and Behavioral Pediatrics. (2012). The lifelong effects of early childhood adversity and toxic stress. Pediatrics, 129(1), e232–e246.

37. Slopen, N., McLaughlin, K. A., & Shonkoff, J. P. (2014). Interventions to improve cortisol regulation in children: A systematic review. Pediatrics, 133(2), 312–326.

38. Wall-Haas, C. L., Kulbok, P., Kirchgessner, J., & Rovnyak, V. (2012). Shared Medical Appointments: Facilitating Care for Children With Asthma and Their Caregivers. Journal of Pediatric Health Care, 26(1), 37–44. http://doi.org/10.1016/j.pedhc.2010.06.007

39. Wing R, Gjelsvik A, Nocera M, McQuaid EL. Association be- tween adverse childhood experiences in the home and pediatric asthma. Ann Allergy Asthma Immunol. 2015;114(5):379-384.

8.0 Biospecimen Collection and/or Bank Administration

8.1 * TYPE OF SPECIMENS (check all that apply): (REQUIRED)

- [x] Blood (provide amount below)
- [ ] Tissue (describe below)
- [x] Other type of biospecimen, such as sputum, cerebrospinal fluid, buccal swabs, etc. (describe below)
- [ ] Existing/archival materials (name source below)

Briefly describe the types of biospecimens that will be collected. Provide the amount of blood, if applicable. For leftover/existing/archival material, identify the source:

Biomarker Collection:

Summary: Participants will be asked to provide a blood,, and nasal swab sample for genetic and serologic testing of genetic and biologic markers related to adversity and the specified health outcomes. At the moment there are no identifiable chemicals or 'Toxic Stress' gene. It is unlikely that a specific toxic stress chemical or gene will ever be identified, because toxic stress is considered to be the result of genetic and environmental risk factors.

All participants will be asked if their cells may be Cryopreserved (frozen) for future cell immortalization. All participants will be asked if they would be interested in being re-contacted for future studies.

Heel Stick for Dried Blood Spot Collection: Participants that are less than 1 year (12 months) of age, a heel stick will be performed to collect 3-5 dried blood spots on filter paper. This amounts to 375-500 microliters of blood per participant. This procedure is similar to newborn screening test that occurs at birth. Specimens will be stored and later DNA/RNA and protein biomarkers related to stress will be extracted from the samples and analyzed for the presence of genes and biomarkers thought possibly to be related to stress.

Venipuncture for Blood Sample: Venipuncture is performed with a needle and syringe to draw approximately of 7 ml (participant's 1-5 years old) to 11.5 ml (participants > 5 years old) of blood from a vein in the arm. DNA/RNA and plasma will be extracted from the samples and analyzed later for the presence of genes and biomarkers thought possibly to be related to stress.

Collection of Microbiome and DNA using oral and nasal swabs: Buccal mucosal and nasal swabs will be collected from all participants using the MoBio collection tubes. The DNA will be extracted from the samples and later analyzed for the presence of genes and chemicals thought possibly to be related to adversity. The use of human bacteria will be used to determine whether bacteria of specific types of bacteria are associated with adversity.

Measurement of Genetic Ancestry: Although we will use a questionnaire to assess racial and ethnic background, among racially admixed populations such as Latinos or African Americans, most participants will not know their true racial background or racial admixture proportions. Genetic admixture can be measured and adjusted for just like any other quantitative variable. There is evidence that racial/ethnic groups have varied physiological responses to stress. Measuring Genetic Ancestry will allow for better identification of genes or proteins associated with these varies responses. This will enable future studies of adversity through an approach presently known as admixture mapping. Information about racial ancestry will not be shared with study participants.

Freeze Cells (cryopreservation): Cells will be frozen which someday may be used to analyze the type of cells and what types of genes are turned on or off (gene expression).

Cell immortalization: In addition to saving the left over samples, in some instances, the researchers may "immortalize" the cells collected from the child. This will provide additional DNA which can then be studied in future research. Cell immortalization means that the cells may be changed in a laboratory so that they will grow and divide continuously. Immortalized cells are an important resource, and if cells from thechild's sample are changed in this way, they may be used by researchers for many years in the future to develop new drugs, tests, treatments or products. In some cases these may have potential commercial value. There are no plans to share future profits with the caregiver and child. See Appendix for Age-Appropriate Biomarker and Specimen Collection Protocol.

Stool Sample: Parent will be provided a home stool collection kit at T1 and will bring the sample at their next schedule visit (T2).

8.3 * SPECIMENS ARE: (check all that apply): (REQUIRED)

- [ ] Leftover specimens from a clinical diagnostic or therapeutic procedure
- [x] Specimens collected for research purposes only (including extra samples taken during a clinical procedure)
- [ ] Other

8.4 * FUTURE SPECIMEN USE: Will any specimens or portions of specimens be retained after the study is over for possible use in future research studies: (REQUIRED)

(•) Yes ( ) No

8.5 * SPECIMEN BANKING - CONSENT METHOD: Consent for retaining specimens for future research studies will be obtained via (check all that apply): (REQUIRED)

- [x] Specimen section within a main research study consent form
- [ ] Separate specimen consent form
- [ ] UCSF surgical consent form with tissue donation brochure

8.6 * SPECIMEN DESTINATION: Indicate where specimens will ultimately be stored: (REQUIRED)

Outside Entities: Indicate where specimens will be sent if they will not remain at UCSF (choose at least one; check all that apply):

- [ ] Cooperative group bank
- [ ] NIH
- [ ] Other university or collaborator
- [ ] Industry sponsor
- [ ] Other
- [x] N/A - all specimens will remain at UCSF

Internal Storage: If specimens will remain at UCSF, in what kind of facility will they reside (choose at least one; check all that apply):

- [ ] UCSF repository/bank being established under this protocol
- [x] Existing UCSF specimen repository/bank with IRB approval
- [ ] National cooperative group bank housed at UCSF
- [ ] Other location at UCSF (please describe)
- [ ] N/A - no specimens will be retained at UCSF facilities

Please provide the name of the department, the program, and the physical location where the specimens will be housed. If the specimens will be stored in an already established bank, provide the name of the bank and its iRIS approval number.:

UCSF Mission Bay Campus, Adversity BioCore (ABC) Biobank, 1550 4th Street, Bldg 19B, Room 582, San Francisco, CA 94143 Contact: Neeta Thakur, neeta.thakur@ucsf.edu, 415 514-9931

8.8 * CLINICAL FOLLOW-UP DATA: Will clinical follow-up data be linked to specimens (i.e., will medical record information continue to be abstracted after the specimen is collected): (REQUIRED)

( ) Yes (•) No

Provide duration of follow-up or 'indefinitely':

8.9 * UCSF-BANKED SPECIMENS - LINKING AND SHARING OF IDENTIFIERS: (REQUIRED)

- [x] Samples are completely de-identified before being added to the bank/repository. There is no way to link the specimens back to the subjects.
- [ ] Samples are coded and researchers are able to link the specimens to specific subjects.
- [ ] Samples are stored with direct identifiers in the repository.

If there is truly no way to link the specimens back to the subject's identity, the consent form should specifically say that participants will not be able to request destruction of remaining samples after the study is over.

8.11 DISTRIBUTION: Specimens banked at UCSF may be made available to (check all that apply):

- [x] UCSF researchers
- [x] Non-UCSF researchers
- [ ] Industry
- [ ] None of the above - specimens will be retained and used within our own research program

8.12 UTILIZATION REVIEW: Is there a formal utilization review process for distribution of specimens:

( ) Yes (•) No

9.0 Drugs and Devices

9.1 * DRUGS AND/OR BIOLOGICS: Are you STUDYING any drugs and/or biologics that are either approved or unapproved: (REQUIRED)

( ) Yes (•) No

If you have questions about FDA requirements for drug or device research, you can send an email to request a consult.

Note: This question is frequently answered incorrectly. If any drugs or biologics, approved or unapproved, are being administered under this protocol, you should check 'Yes' unless you are *absolutely* sure that NONE of the drugs are part of the research protocol. Tip: Ask the PI or the sponsor if you are not sure how to answer this question.

9.3 * MEDICAL DEVICES: Are you STUDYING any medical devices, in vitro diagnostics, or assays that are either approved or unapproved:(REQUIRED)

○ Yes ◉ No

If you have questions about FDA requirements for drug or device research, you can send an email to request a consult.

10.0 Sample Size and Eligibility Criteria

10.1 ENROLLMENT TARGET: How many people will you enroll:

700

If there are multiple participant groups, indicate how many people will be in each group:

The first randomization (1) will take place just after baseline data collection, into one of three ACEs Screen groups (No Screen, Identified Screen, De-Identified Screen), with the No Screen group proceeding to standard usual Care, and the Identified/De-Identified Screen groups proceeding to Anticipatory Guidance. The number of participants in each group will be equally divided - approximately 233 per group.

The second randomization is for study subjects who have an ACES =>1, taking place a month after Anticipatory Guidance at T2, into one of two Preventive Intervention groups (Care Coordination or Resiliency Clinic). The number of participants will also be equally divided amongst the two Intervention groups.

At the time of transfer, 1443 subjects were enrolled. 888 failed screening. 318 completed the study and 237 withdrew.

Enrollment in closed. Study procedures are complete. The study is in data analysis phase.

10.3 SAMPLE SIZE JUSTIFICATION: Explain how and why the number of people was chosen. For multi-site studies, this is referring to the number that will be enrolled across all sites:

Sample Size/Power: Sample size and power estimates for comparison of the two ACEs formats in Phase 3 are based on alpha = 0.05 and 2-sided t-tests. Estimates of associations between ACE items with child biomarkers, and behavioral health outcomes in similar studies suggest effect sizes that are small to moderate, with standardized effects ranging from d=0.30 to 0.48. [61,62,63,64] Effect sizes for associations between ACEs or early psychosocial exposures with health diagnoses in related adult and child literatures similarly suggest small to moderate effect sizes of OR=1.6-1.9 across a range of diagnoses (e.g., acute upper respiratory infections, otitis media, viral infections, eczema, UTI, and asthma). [65,66] We intend to obtain a sample of n=555 (n=185 per arm). This sample size will provide power to exceed 0.80 for evaluation of the association between ACEs with the biomarkers, child behavioral health indicators, and child diagnoses. Within the Identified arm, the ratio of ACEs items to respondents exceeds 5.0 for factor analytic techniques.[67,68]For tests directly comparing between group differences across arms (identified vs. de-identified screening format), this sample size (n = 370 total, n=185 per arm) will additionally allow for detection of an approximate 11% difference in disclosure rates for a given number of ACEs69 or standardized

units mean difference in number of ACEs. At the projected sample size for each group, power to discriminate at this level between the two arms will be at least 80%. The total number of patients who came to CPCC for well visits in 2013 was 8,714. 12% were Spanish speakers. Of all patients, 4,760 were ages 0-3, 971 ages 4-5, and 2,160 ages 6-12. Given the large number of patients seen annually at CPCC, coupled with our previous success implementing the FIND study, we do not expect to have difficulty recruiting this sample size.

10.4 * PARTICIPANT AGE RANGE: Eligible age ranges: (REQUIRED)

- [x] 0-6 years
- [x] 7-12 years
- [ ] 13-17 years
- [ ] 18-64 years
- [ ] 65+

10.5 * STUDY POPULATIONS: Data will be collected from or about the following types of people (check all that apply): (REQUIRED)

- [ ] Inpatients
- [x] Outpatients
- [ ] Family members or caregivers
- [ ] Providers
- [ ] People who have a condition but who are not being seen as patients
- [ ] Healthy volunteers
- [ ] Students
- [ ] Staff of UCSF or affiliated institutions
- [ ] None of the above

10.6 * SPECIAL SUBJECT GROUPS: Check the populations that may be enrolled: (REQUIRED)

- [x] Children / Minors
- [ ] Adult subjects unable to consent for themselves
- [ ] Adult subjects unable to consent for themselves (emergency setting)
- [ ] Subjects with diminished capacity to consent
- [ ] Subjects unable to read, speak or understand English
- [ ] Pregnant women
- [ ] Fetuses
- [ ] Neonates
- [ ] Prisoners
- [ ] Economically or educationally disadvantaged persons
- [ ] None of the above

If not already addressed in the Background and Significance questions in the Research Plan section or elsewhere, explain why it is appropriate to include the types of subjects checked above in this particular study:

Describe the additional safeguards that have been included in the study to protect the rights and welfare of these subjects and minimize coercion or undue influence:

Here are some examples:

- evaluating capacity to consent for individuals who may be decisionally impaired (specify how)
- calibrating payment amounts to be non-coercive for the financially disadvantaged

- conducting more in-depth evaluations of subjects' understanding of the study and the voluntary nature of participation
- involving advocates in the consent process

More information and other safeguards are described here: **Vulnerable Subject Populations** and **Recruiting Staff and Students**.

10.7 INCLUSION CRITERIA: Briefly describe the population(s) that will be involved in this study. Include anyone that data will be collected from or about (e.g. patients, healthy controls, caregivers, providers, administrators, students, parents, family members, etc.):

Inclusion Criteria:

1. Children ages 3 months-11 years receiving well-child clinic services at UCSF Benioff Children's Hospital Oakland's Primary Care department, and
2. Parents/primary caregivers of the children above, ages 18 and over, who are also legal guardians capable of consent.
3. English and Spanish speaking

10.8 EXCLUSION CRITERIA: List any exclusion criteria (e.g. reasons why someone would not be included in the study):

Exclusion Criteria:

1. Children who are dependents of the court
2. Caregiver active drug or alcohol use or dependence that, in the opinion of the site investigator, would interfere with full participation in the study
3. Child or caregiver serious illness (mental or physical; requiring systemic treatment and/or hospitalization) or significant developmental disability that would interfere with full participation in the study
4. Inability or unwillingness of subject or legal guardian/representative to give written informed consent.

10.9 * RESEARCH CONDUCTED ON PATIENT CARE WARDS: Do any study activities take place on any patient care units including inpatient wards, peri- or post-operative care units, operating rooms, or in the Emergency Department at UCSF Health medical facilities: (REQUIRED)

○ Yes ◉ No

10.11 * EMERGENCY DEPARTMENT: Does your protocol or study involve any of the following patient related activities in the emergency department (e.g. subject identification, recruitment, consent, blood draws, specimen retrieval, involvement of ED staff (nursing, tech, and/or physician), or any other ED based procedures): (REQUIRED)

○ Yes ◉ No

11.0 Inclusion of Minors in Research

11.1 REGULATORY CATEGORIES OF RESEARCH: Select all the regulatory categories that apply:

- [x] No greater than minimal risk (45 CFR 46.404, 21 CFR 50.51)
- [ ] Greater than minimal risk but presenting prospect of direct benefit (45 CFR 46.405, 21 CFR 50.52)
- [ ] Greater than minimal risk (though only a minor increase over minimal risk) and no prospect of direct benefit but likely to yield generalizable knowledge about the subjects disorder or condition (45 CFR

46.406, 21 CFR 50.53)

☐ Research not otherwise approvable which presents an opportunity to understand, prevent, or alleviate a serious problem affecting the health or welfare of children (45 CFR 46.407, 21 CFR 50.54)

Explain why the research in this study falls under the above category or categories:

The potential benefits to subjects participating in this study include 1) the identification of adverse childhood experiences and therefore the chance to receive support and services through the primary care clinic to mitigate the mental and physical health consequences, 2) the opportunity to learn about the link between ACEs and health, 3) links to ACEs-related referrals and/or the development of skills to manage stress and its health impacts, and 4) for caregivers to increase capacity to understand and manage the potentially toxic effects of stress on their children.

While caregivers may feel that mandated reporting requirements to Child Protective Services and/or law enforcement is a risk, mandated reporting is a benefit to the child being abused or neglected.

Discussing adversity and trauma may be triggering and emotionally charged for a number of caregivers and children, however, the primary care department staff including medical providers and social workers, will be available to help support awareness, education, growth and recovery.

Blood draws to collect bio-specimens will hurt a little and will possibly leave a bruise. However, elucidating the associations between adversity in childhood, biomarkers of stress physiology and lifelong health impacts will provide benefits to society by 1) providing insight into the underlying biochemical and immunologic pathways between stress and health, 2) helping lay the foundation for multi-sector, multi-disciplinary approaches to mental and physical health and well-being, and 3) guiding future prevention work and interventions related to adverse childhood experiences.

11.2

MINORS CONSENTING: Will this study enroll minors who can legally consent for themselves (as in the case of emancipated minors or minors being treated for pregnancy or drug use without their parents knowing). This is different from agreeing to be in the study even when their parents are the ones providing 'official' consent, which we refer to as 'providing assent':

Note: This is very rare and the answer is usually 'No.'

○ Yes ◉ No

11.3

PARENTAL PERMISSION VS. WAIVER: Please review the guidance to see under what circumstances the IRB can waive parental permission.

☑ Parental permission will be obtained

☐ Waiver of parental permission is requested: The waiver meets the provisions for a waiver of consent (i.e., the research poses minimal risk, it could not practicably be carried out without the waiver of parental permission, AND the waiver will not adversely affect the rights and welfare of the minor participants (45 CFR 46.116(d))

☐ Waiver of parental permission is requested: Parental permission is not a reasonable requirement to protect the minor (e.g. neglected or abused children) or parental knowledge of the study may endanger the health or welfare of the minor (45 CRF 46.408(c))

Provide details on the other protections that will be in place:

The research team conducting this study holds a high standard of respect for all adult and child participants, which includes transparency about the purpose, possible risks and benefits to subjects. Informed consent of adult participants (parents/guardians) will be afforded as much time as is necessary for both parents/guardians and children to be informed about what their participation in the study would entail and given an opportunity to ask questions and have them answered in a developmentally appropriate way. For children ages 7-11, declining assent will constitute declining participation in the study even if parents/guardians consent.

11.4 ASSENT OF MINORS OR WAIVER: Please review the guidance to see under what circumstances the IRB can waive assent.

- [x] Assent of children developmentally and psychologically able to provide assent will be obtained
- [ ] Waiver of assent is requested: The capability of some or all of the children is so limited that they cannot reasonably be consulted
- [ ] Waiver of assent is requested: The research holds out a prospect of direct benefit that is important to the health or well-being of the children and is available only in the context of the research
- [ ] Waiver of assent is requested: The activities involving the minor are limited to chart review or the something equally innocuous
- [ ] Waiver of assent is requested: It is not culturally appropriate to involve the minor in the decision to participate (e.g. some foreign research)

11.5 DOCUMENTATION OF PERMISSION AND ASSENT: (select all that will be used):

- [x] Permission form addressed to the parents
- [ ] Simplified assent form addressed to the child, 7-12 years old (parents get separate form)
- [ ] Assent form addressed to the child, 13 years and older (for subjects and parents)
- [ ] Assent form addressed to the child, 13 years and older (parents get separate form)

Check one:

- (•) One parent's signature will be obtained
- ( ) Two parents' signatures will be obtained

If this study is approvable under regulatory category .405 and you plan to get permission from only one parent, explain why you think one parent's permission is sufficient:

11.6 WARDS OF THE STATE: Might this study enroll wards of the state:

( ) Yes (•) No

12.0 Recruitment and Consent

12.1 * COMPETITIVE ENROLLMENT: Is this a competitive enrollment clinical trial? By competitive enrollment, we mean that sites who do not enroll participants early may not get to participate at all: (REQUIRED)

( ) Yes (•) No

12.2 * SUBJECT IDENTIFICATION METHODS: What kinds of methods will be used to identify potential participants for recruitment (check all that apply): (REQUIRED)

- [x] Review of patients' conditions, history, test results, etc. (includes patients seen in clinic, scheduled for surgery, a procedure, imaging, or tests, or seen in the Emergency Department as well as searching through medical record data for possible cohort identification)
- [ ] Already approved recruitment registry
- [x] Re-contact of participants from the investigators' previous studies
- [ ] Referrals from colleagues (attach the 'Dear Colleague' letter or other recruitment materials you will provide to colleagues)
- [x] Referrals from the community / word of mouth
- [x] Advertisements (flyers, brochures, radio or t.v. ads, posting on clinical research sites or social media, presentation of the study at community events/media, etc.)
- [ ] Online recruiting tool (describe below)
- [ ] CTSI Recruitment Services unit
- [ ] Posting on UCSF Clinical Trials, ClinicalTrials.gov or other publicly available clinical trial website
- [ ] Other method (describe below)

Attach your recruitment materials (e.g., flyers, ads, recruitment letter templates, email text, etc.) in the Other Study Documents section of the Initial Review Submission Packet Form.

* Provide details about the subject identification methods: **(REQUIRED)**

Potentially eligible study subjects will be identified in the UCSFBCHO EPIC, the electronic medical records system. Each afternoon study staff will review scheduled well appointments and identify those who meet the age requirements, according to current recruitment needs. Each day's list of potential study subjects will be identified on the clinic providers schedule with a sticker indicating the patient and caregiver are eligible for recruitment. When the patient is checked into Primary Care for their appointment, registration staff will notify the Study Coordinator(s) of their presence by paging them. Study staff will then approach the caregiver and child while they are waiting for their clinic appointment, and provide them with a study flyer (see Appendix). If the family is interested, the Study Coordinator will invite them to further discuss the study and review the consent materials.

* Did all the participants of previous studies provide permission to be contacted for future studies: **(REQUIRED)**

(•) Yes ( ) No

12.3 * SEARCHING OF MEDICAL RECORDS: (REQUIRED)

Whose patients are they:

- [x] Investigators' own patients or patients seen within the same practice
- [ ] Patients not under the care of the investigators

How and by whom will records be accessed and searched (check all that apply):

- [x] Self-search in APeX or other medical records source
- [ ] Self-search using UCSF's Research Cohort Selection Tool
- [ ] CTSI Consultation Service Recruitment Services
- [ ] UCSF Academic Research Services (ARS)
- [ ] University of California Research Exchange (UC ReX)
- [ ] Other method (describe below)

12.4 DETERMINATION OF ELIGIBILITY: How, when, and by whom will eligibility for recruitment be determined:

Eligibility will be determined by trained study personnel using the eligibility screening form. Families that meet the eligibility criteria will be presented the consent form.

At time of transfer, study is closed to enrollment and in the data analysis phase.

12.5 * INITIATION OF CONTACT: Who initiates contact (check all that apply): (REQUIRED)

- [x] Investigators/study team
- [ ] UCSF recruitment unit (e.g. CTSI Consultation Services)
- [ ] Potential participant
- [ ] Other (explain below)

12.6 * HOW IS CONTACT INITIATED: (check all that apply): (REQUIRED)

- [x] In person
- [ ] Phone
- [ ] Letter / email
- [ ] Website or app

☐ Other (explain below)

12.7 RECRUITMENT PLAN: Based on the checkboxes you chose above, please provide a narrative describing your recruitment plan. We want to know:

- **Who is conducting the search for potential participants, and how?**
- **How are potential subjects being approached for recruitment? By whom, and when?**

If there will be more than one participant group (e.g. patients, healthy controls, caregivers, family members, providers, etc.), provide details about the recruitment plans for each group. (Recommended length - 100-250 words)

Recruitment will be done solely within the Claremont Clinic. Providers will talk to potentially eligible families to see if they might be interested and study flyers will be posted and made available throughout the clinic. The study team will also be actively recruiting in the clinic as families come in to clinic for their scheduled visits.

At time of transfer, study is closed to enrollment and in the data analysis phase.

12.8 * CONSENT METHODS: How will permission to participate (i.e., informed consent) be obtained from each potential participant. If there will be multiple groups and different plans for consenting each, check all that apply. See the orange Help bubble to the right for more detailed guidance. Participants will (check all that apply): (REQUIRED)

☑ Sign a paper consent form at the end of the consent discussion (signed consent)
☐ Sign an electronic consent form using DocuSign (signed consent)
☐ Provide online consent through an app, a website, or a survey tool such as Qualtrics or REDCap (waiver of signed consent)
☐ Be told about the study and be given a handout/information sheet and be asked if they agree to participate (verbal consent - waiver of signed consent)
☐ Complete the study activities and turn in materials, as in the case of a completed survey that is placed in a drop box or mailed to the study team (implied consent - waiver of signed consent)
☐ Not be able to provide consent and will have a family member consent for them, as in the case of a critically ill or unconscious patient (surrogate consent)
☐ Not able to provide consent (emergency medicine, greater than minimal risk waiver/alteration of consent - requires an approved community consultation plan)
☐ Not able to provide consent (emergency medicine, minimal risk waiver/alteration of consent)
☐ Not know about the study, as in the case of chart reviews or observations of public behavior (waiver of consent)
☐ Other method (describe below)

Attach your consent form, information sheet, or electronic consent text in the Informed Consent Documents section of the Initial Review Submission Packet Form.

12.9 * CONSENT PROCESS: Describe the process for obtaining informed consent, including details such as who will have the consent discussion and when participants will be asked to sign the consent form in relation to finding out about the study: (REQUIRED) We encourage researchers to review our guidance on obtaining and documenting informed consent.

- **If there are multiple groups being consented differently, provide details about the consent process for each group.**
- **If you are relying on verbal or implied consent, provide details about how that will happen.**
- **For studies using online recruitment and consent or consent via mail, provide details here.**

Informed consent will be solicited in person at Claremont Primary Care Clinic, UCSF Benioff Children's Hospital Oakland, in a private exam room. Study staff will review the consent form with the caregiver in their language of preference (English or Spanish). All aspects of the written consent form will be discussed in detail and caregivers and children will be offered an opportunity to have all questions answered. The process will include obtaining adult consent and parent/guardian permission for child

participation. Potential subjects will be offered the opportunity to have study staff leave the room so they may privately discuss their participation with family or others, before signing the consent form.

The consent process will include obtaining adult consent and parent/guardian permission. It is the caregiver /child dyad that will child participate in the study. Potential subjects will be offered the opportunity to have study staff leave the room so they may privately discuss their participation with family or others, before signing the consent form.

In addition to consent of the adult subject, oral assent of minors ages 7-11 will be sought and required for participation in the study. In cases of children 7-11 years, once an adult subject has consented, the Study Coordinator will explain that it is important to obtain the child's agreement to participate, as well. The Study Coordinator will then explain in developmentally appropriate terms the nature of the Resiliency Clinic Pilot Study and what his/her experience of it would be; assess the child's understanding of this information and anything that may influence the child's evaluation of the situation; and solicit the child's agreement to participate. Assent of minors ages 7-11 will be obtained orally and documented by the Study Coordinator on the consent form and study file.

The Study Coordinator will determine capacity to consent based upon a discussion of the study with a prospective adult subject during the consent process, followed by a series of questions to assess the person's understanding of: the purpose of the research, the foreseeable risks and anticipated benefits of study participation; the prospective adult subject's understanding of the voluntary nature of research and the elements of consent, including the right to be informed about appropriate alternative interventions.

At time of transfer, study is closed to enrollment and in the data analysis phase.

* It is important that the people obtaining consent are qualified to do so. Briefly describe the training and experience these individuals have in obtaining informed consent: **(REQUIRED)**

Mindy Benson, PNP, MS will conduct and oversee all consenting. Mindy has been a study coordinator or investigator since 1993. She is fully trained and experienced with administering consents. CITI trained.
Assisting Mindy will be:
Roberto Mok, LVN. Robert has been a study coordinator since 2009. He is fully trained and experienced with administering consents. CITI trained.

Cherri Harris, LVN. Cherri has been a study coordinator since 2013. She is fully trained and experienced with administering consents. CITI trained.

Maoya Alqassari, BA. Study coordinator since 2014. She is fully trained and experienced with administering consents. CITI trained.

Rigoberto Del Torro, BA, Study coordinator since 2015. He is fully trained and experienced with administering consents. CITI trained.

Nai Pharm, BA, Study coordinator since 2017. She is fully trained and experienced with administering consents. CITI trained.

12.10 * CONSENT COMPREHENSION: Indicate how the study team will assess and enhance the subjects' understanding of study procedures, risks, and benefits prior to signing the consent form (check all that apply): (REQUIRED)   Tip: Review the Consent Comprehension - Learning Notes in the Help bubble at the right for specific questions that can be asked to assess comprehension, consider using the UCSF Decision-Making Capacity Assesment Tool, and review our guidance on obtaining written or verbal informed consent for more detail on how to conduct the assessment.

- [x] The study team will engage the potential participant in a dialogue, using open-ended questions about the nature of the study or the experimental treatment, the risks and benefits of participating, and the voluntary nature of participation
- [ ] Potential participants will be asked or shown a series of questions to assess their understanding of the study purpose, procedures, risks and benefits, as well as the voluntary nature of participation (especially appropriate when the consent process happens online or through a mobile health app)
- [ ] Other method (describe below):

Provide details of the other approaches that will be used, if using another method to assess comprehension:

12.11 *** DECEPTION: Does this study rely on some deception or misinformation about what the researchers are observing to get valid data? (REQUIRED)**

◯ Yes ◉ No

12.14 **TIME: What is the estimated time commitment for participants (per visit and in total):**

After consenting to be in the study:

Visit 1 as it's done in conjunction with the scheduled clinic visit may add up to 30 minutes.
Visit 2 may take up to 2 1/2 hours.
Visit 3 may take up to 1 1/2 hours.
Visit 4 may take up to 2 1/2 hours.

IMPORTANT TIP: Ensure this information is consistent with the information provided in the consent form.

12.17 **OTHER ALTERNATIVES: Describe other alternatives to study participation, if any, that are available to prospective subjects:**

13.0 Waiver of Consent/Authorization for Recruitment Purposes

This section is required when medical records may be reviewed to determine eligibility for recruitment.

13.1 *** PRACTICABILITY OF OBTAINING CONSENT PRIOR TO ACCESS: Study personnel need to access protected health information (PHI) during the recruitment process and it is not practicable to obtain informed consent until potential subjects have been identified: (REQUIRED)**

◉ Yes

If **no**, a waiver of consent/authorization is NOT needed.

13.2 *** RISK TO PRIVACY: A waiver for screening of health records to identify potential subjects poses no more than minimal risk to privacy for participants:**

◉ Yes

If **no**, a waiver of authorization can NOT be granted.

13.3 *** RIGHTS/WELFARE: Screening health records prior to obtaining consent will not adversely affect subjects' rights and welfare:**

◉ Yes

If **no**, a waiver of authorization can NOT be granted.

13.4 *** IDENTIFIERS: Check all the identifiers that will be collected prior to obtaining informed consent:**

- [x] Names
- [x] Dates

- [ ] Postal addresses
- [ ] Phone numbers
- [ ] Fax numbers
- [ ] Email addresses
- [ ] Social Security Numbers*
- [x] Medical record numbers
- [ ] Health plan numbers
- [ ] Account numbers
- [ ] License or certificate numbers
- [ ] Vehicle ID numbers
- [ ] Device identifiers or serial numbers
- [ ] Web URLs
- [ ] IP address numbers
- [ ] Biometric identifiers
- [ ] Facial photos or other identifiable images
- [ ] Any other unique identifier
- [ ] None

Note: HIPAA rules require that you collect the minimum necessary.

13.5 * HEALTH INFORMATION: Describe any health information that will be collected prior to obtaining informed consent:

Health information that is relevant to the study's inclusion and exclusion criteria will be used to determine participant's eligibility. Basically, children between 3 months and 11 years of age who are being seen for a well-child visit.

Note: HIPAA requires that you collect the minimum necessary.

13.6 * DATA RETENTION/DESTRUCTION PLAN: Describe your plan to destroy any identifiable data collected to determine eligibility for recruitment. This should be done at the earliest opportunity. If you plan to retain identifiable recruitment data, provide the justification for doing so:

There's no plan to keep any recruitment data since we are visually screening in APEX. No recruitment data will be collected and retained.

14.0 Risks and Benefits

14.1 RESEARCH-RELATED RISKS: Check if your study involves any of these specific research-related risks to participants that may need to be disclosed in the consent form:

- [x] Physical discomforts or pain
- [ ] Risks to employment, or social or legal standing
- [x] Risk that the study team may observe possible evidence of child abuse, elder abuse, or a threat to self or others that they are required to report

For reportable information, include details of the reporting plan below. (See the Help link for Mandated Reporter child and elder abuse resources.)

* For any boxes checked above, describe how you will minimize these risks and discomforts, e.g., adding or increasing the frequency of monitoring, additional screening to identify and exclude people with diminished kidney or liver function, or modification of procedures such as changing imaging studies to avoid giving contrast agent to people who are more likely to suffer side effects from it, etc.: **(REQUIRED)**

Discomfort as a result of heel stick, venipuncture, oral or nasal swabs will be addressed by Study Coordinators, who are medical providers. Participants will be informed that In the event of injury, Dr. Dayna Long will be notified.

In certain situations involving the safety of participant, participant's child and others, we are required by law to disclose information to appropriate authorities and others. These situations include:

- Suspected child abuse and neglect
- Suspected elder/dependent adult abuse and neglect
- If you threaten to harm another person, that person and/or the police must be informed
- When necessary, if you pose a serious threat to your own health and safety, such as being suicidal.

Should mandated reporting become necessary, we will make every effort to involve the participant, work with the participant and keep the participant informed.

14.2 * RISKS: Describe any anticipated risks and discomforts not listed above: (REQUIRED)

Number of visits: Participation in the study will involve at least 4 study visits and up to 6 additional clinic visits. Participants may experience an inconvenience by having to show up the for a 2-hour study visit while clinical exam measurements, heel-stick/blood, oral and nasal sampling and questionnaire data are being collected.

Emotional discomfort: Answering the questionnaires and completing measures may make participants feel uncomfortable or raise unpleasant thoughts or feelings. Participants may skip questions. Psychosocial support will be available for any participant upset by the experience.

Randomization risks: Participants will be assigned to a treatment program by chance, and the treatment they receive may prove to be less effective or have more side effects than the other study treatments or other available treatments.

Participation in the Care Coordination intervention is associated with minimal risk of discomfort.

Participation in the Resiliency Clinic: is associated with a low risk of psychological distress. To date, there are no known physical risks associated with a paired psychoeducational/medical visit model. Group participation: If randomized to Group G, Resiliency Clinic, participation will include being part of a group with other families. Each group will have about 6 adult caregivers and 6 children. There is some risk that confidentiality and privacy will not be maintained by other group member(s). All group members will be asked to verbally agree to maintain the confidentiality and privacy of all members. However, because group members are not legally bound to confidentiality, it cannot be assured. Confidentiality will be discussed with all participants when the group begins.

Heel Stick and Venipuncture for Blood Sample: Withdrawal of blood via a heel stick or from a forearm vein (venipuncture) may cause pain and bruising at the site of needle puncture. There is a remote possibility of infection from the venipuncture and some people may faint when blood is drawn.

Nasal swabs: Some people may have mild discomfort with this procedure.

Testing Samples of My Child’s Blood for Toxic Stress Chemicals and Genes: At the moment

there is no identifiable chemicals or ‘Toxic Stress Gene’ and it is unlikely that a specific toxic stress chemical or gene will ever be identified, because toxic stress is considered to be the result of genetic and environmental risk factors. Genetic and chemical marker information that results from this study does not have medical or treatment importance at this time. However, there is a risk that information about taking part in a genetic study may influence insurance companies and/or employers regarding a child’s health. To further safeguard participant privacy, genetic and chemical marker information obtained in this study will not be placed in the medical record. Although child name will not be with the sample data, it will include data such as exposure to ACEs, health status, age, race and other traits. It is possible that study finding could one day help people of the same race, ethnicity, or sex as the participant. However, it is also possible through these kinds of studies that genetic traits might come to be associated with the participant’ s group. In some cases, this could reinforce harmful stereotypes.

Confidentiality: Participating in a survey, donating blood, oral or nasal samples may involve a loss of privacy, but participant information will be handled as confidentially as possible. Study data will be physically and electronically secured. As with any use of electronic means to store data, there is a risk of breach of data security. No identifiers will be included in any published reports. The UCSF Principal Investigator, Dr. Neeta Thakur of Adversity BioCore Biobank, and select UCSF staff members will have access to information about participants but they will not release any identifying information to researchers using his/her blood, oral or nasal samples.

14.3 MINIMIZING RISKS: Describe the steps you have taken to minimize the risks/discomforts to subjects. Examples include:

- **designing the study to make use of procedures involving less risk when appropriate**
- **minimizing study procedures by taking advantage of clinical procedures conducted on the study participants**
- **mitigating risks by planning special monitoring or conducting supportive interventions for the study**
- **having a plan for evaluation and possible referral of subjects who report suicidal ideation**

Participants will be informed prior to consent to anticipate that study visits will take up to 2 hours.

For participants randomized to group intervention, confidentiality will be discussed with all participants when the group begins.

Discomfort as a result of heel stick, venipuncture, oral or nasal swabs will be addressed by Study Coordinators, who are medical providers.

Complete procedures will be in place to protect participant confidentiality in the storage and transfer of data (e.g., encryption, codes, and passwords.)

Participants will be informed that In the event of injury, Dr. Dayna Long will be notified. In the event of emotional distress, a mental health clinician will be notified.

14.5 * BENEFITS: (REQUIRED) Note: These are the benefits that the IRB will consider during their review. They are not necessarily appropriate to include in the consent form.

Possible immediate and/or direct benefits to participants and society at large (check all that apply):

- [x] Positive health outcome (e.g. improvement of condition, relief of pain, increased mobility, etc.)
- [ ] Closer follow-up than standard care may lead to improved outcomes or patient engagement
- [ ] Health and lifestyle changes may occur as a result of participation
- [x] Knowledge may be gained about their health and health conditions
- [ ] Feeling of contribution to knowledge in the health or social sciences field
- [x] The research presents a reasonable opportunity to further the understanding, prevention, or alleviation of a serious problem affecting the health or welfare of children
- [ ] Other benefit (describe below)
- [ ] None

14.6 RISK TO BENEFIT RATIO: Explain why the risks to subjects are reasonable in relation to anticipated benefits, if any, to the participant or society:

Participation may inform future research and practice to help mitigate the effects of toxic stress. Participants may also benefit by receiving referrals to needed resources and learning ways to manage stress and promote health. For caregivers, specifically, it could potentially increase their capacity to understand and manage the toxic effects of stress on their children.

Because of the lack of knowledge regarding the clinical significance of genotypes, respondents will not be supplied information about their genotype, and will receive no genetic counseling. In the event that a significant genetic association is discovered, we will notify the clinic and providers of this finding. Notification will come in the form of a letter in which we will summarize our findings and refer interested persons to a detailed description in the press. In addition, PIs/Dr. Thakur and collaborators of this study will make formal presentations of the results at national and local meetings.

14.7 * DATA AND SAFETY MONITORING: Do you have a Data and Safety Monitoring Plan (DSMP) for this study (A DSMP is required for Greater than Minimal Risk research): (Click the Help link for guidance

on risk determination) (REQUIRED)

○ Yes ◉ No

This is not required for minimal risk research but the UCSF IRB strongly recommends one to ensure the data collected are adequate to meet the research aims:

15.0 Confidentiality, Privacy, and Data Security

15.1 PROTECTING PRIVACY: Indicate how subject privacy will be protected:

- [x] Conduct conversations about the research in a private room
- [x] Ask the subject how they wish to be communicated with – what phone numbers can be called, can messages be left, can they receive mail about the study at home, etc.
- [x] Take special measures to ensure that data collected about sensitive issues do not get added to their medical records or shared with others without the subject's permission
- [ ] Other methods (describe below)

15.2 SENSITIVE DATA: Do any of the instruments ask about illegal or stigmatized behavior:

○ Yes ◉ No

15.3 SIGNIFICANT CONSEQUENCES OF A LOSS OF PRIVACY OR CONFIDENTIALITY: Could a breach of privacy or confidentiality result in any significant consequences to participants, such as criminal or civil liability, loss of state or federal benefits, or be damaging to the participant's financial standing, employability, or reputation:

○ Yes ◉ No

15.4 EXTRA CONFIDENTIALITY MEASURES: Explain any extra steps that will be taken to assure confidentiality and protect identifiable information from improper use and disclosure, if any:

Data collected on study subjects will remain completely confidential. Confidentiality of participants will be maintained by handling individual paper and electronic data by ID number, rather than by name, by storing all individual electronic data in a secure REDCap database and paper data in a locked file cabinet, and not disclosing individual data to anyone. Only ID numbers will be displayed on study files, and corresponding names will be kept in a separate, locked file cabinet and password-protected drive. Hard copies of all study files, including questionnaires and measures, will be kept in a locked file cabinet, in a locked room in the basement of 5275 Claremont Avenue.

Computerized records will be stored in secure databases with passwords for authorized investigators. REDCap is a secure, web-based application designed to support data capture for research studies. REDCap is HIPAA-compliant and widely used by UCSF and other researchers.

Electronic data files will be sent via UCSF secure email to the UCSF statistician for data analysis and summary and stored on a HIPAA protected UCSF server. All transfer of data (which will all be de-identified) between UCSF Benioff Children's and UCSF Parnassus will occur over secure email and stored on HIPAA protected and secure drives. Video will not be sent to UCSF. It will be placed in a locked filing cabinet and the recording downloaded to a secure hard drive at UCSF Benioff Children's Hospital Oakland and then erased from the recording device. Access will be limited to study personnel and non-UCSFBCHO analysts contracted to assist in coding the video data. These analysts will view video over a secure, HIPAA-protected system and will be bound by a written confidentiality agreement.

For the Care Coordination arm, families are going to be screened and connect to basic resources, including mental health resources, using FINDconnect © FINDconnect is a HIPAA compliant, mobile friendly platform, founded at BCHO.

Biospecimens processed by the ABC Bank at UCSF will be de-identified and issued a separate Specimen ID number that is different from the parent-study Subject ID number. Thus, the parent-study Subject ID will

no longer be associated with the specimen. An encrypted key linking the Specimen ID to the Subject ID will be stored on a secure network, will be password protected, and only accessible by Dr. Thakur, and select lab research staff.

Participants will be assured of complete confidentiality of genetic and other biological test results. As with all research data, information gathered by the study will be used only for aggregate analysis, and will not be released with any information that identifies research participants. Information about genotypes, in particular, will be coded and unlinked to individual respondent identifiers. The code to link respondents and their genotypes will be securely stored, and accessible only to the database programmer, to Dr. Thakur and her collaborators. Respondents will be informed that genotyping at loci related to adversities is valuable for research purposes and in aggregate form only.

Home addresses will be provided to a non-UCSF collaborator to correlate address with measure of air pollution and census data. Home addresses will be coded and unlinked to individual respondent identifiers.

Video recordings cannot by nature be completely de-identified, however they will be labeled with ID numbers; no identifying information will be included in the label. After recording a study visit, the recorder will be placed in a locked filing cabinet and the recording downloaded to a secure hard drive and then erased from the recording device. Access will be restricted to study personnel.

Hard copies of all study files, including questionnaires and transcripts, will be kept in a locked file cabinet, in a locked room in the basement of 5275 Claremont Avenue.

Electronic data files and video recordings will be stored on secure, HIPAA-protected UCSFBCHO and UCSF drives.

Information provided by the subjects will remain strictly confidential, with access limited to the project staff and, if applicable, State or Federal regulatory personnel.

The only exceptions to confidentiality, which are mandated by law, are detailed in the consent form. No one but select project staff (the PI, Co-I, and study coordinators) will have access to the master list linking subjects' names to code numbers, and all information obtained will be coded. Additional project staff will have access to de-identified data (coded by ID number only) for the purposes of data analysis. This ID will completely separate from any number already existing in clinic (e.g., MRN number, pt ID, etc.).

This code will be verbally recorded on video recordings and listed with all transcripts and completed surveys. The master list will be locked. Publications or presentation of findings will not include information identifying the subjects. There will be no release of identifiable data.

Hard-copies containing participant information will be destroyed 7 years after the study closed. De-identified data will be kept on a secure network indefinitely for future analyses that will inform future studies. Biospecimens collected during this study will be deposited in the UCSF ABC Bank for analysis and for indefinite storage.

15.5 * REPORTABILITY: Do you anticipate that this study may collect information that State or Federal law requires to be reported to other officials, such as elder abuse, child abuse, or threat to self or others: (REQUIRED)

(●) Yes ( ) No

The confidentiality and privacy section of the consent form should include this as a possible risk of participation.

* Describe the types of reportable information the research team may encounter and provide the details of the reporting plan: **(REQUIRED)**

In certain situations involving the safety of participant, participant's child and others, we are required by law to disclose information to appropriate authorities and others. These situations include:

- Suspected child abuse and neglect
- Suspected elder/dependent adult abuse and neglect
- If you threaten to harm another person, that person and/or the police must be informed
- When necessary, if you pose a serious threat to your own health and safety, such as being suicidal.

Should mandated reporting become necessary, we will make every effort to involve the participant, work with the participant and keep the participant informed.

15.6 CERTIFICATE OF CONFIDENTIALITY: Will this study obtain a Certificate of Confidentiality:

○ Yes ◉ No

15.7 SHARING OF RESEARCH RESULTS: Will there be any sharing of EXPERIMENTAL research test results with subjects or their care providers:

○ Yes ◉ No

15.9 * HIPAA APPLICABILITY: Study data will be: (REQUIRED)

- [x] Derived from a medical record (e.g. APeX, OnCore, etc. Identify source below)
- [ ] Added to the hospital or clinical medical record
- [ ] Created or collected as part of health care
- [ ] Used to make health care decisions
- [ ] Obtained from the subject, including interviews, questionnaires
- [ ] Obtained ONLY from a foreign country or countries
- [ ] Obtained ONLY from records open to the public
- [ ] Obtained from existing research records
- [ ] None of the above
- [ ] Derived from the Integrated Data Repository (IDR) or The Health Record Data Service (THREDS) at SFGH

In addition to signing a consent form, each subject will have to sign the UCSF Research Subject Authorization Form (HIPAA Form). Upload the HIPAA Authorization Form in the Other Study Documents section of the Initial Review Submission Packet Form. Failure to have patients sign the HIPAA Authorization is one of the most common findings from QIU Routine Site Visits. Please call the IRB office at 415-476-1814 if you have questions about HIPAA research requirements.

If derived from a medical record, identify source:

APEX

15.10 * IDENTIFIERS: Check all identifiers that will be collected and included in the research records, even temporarily: (REQUIRED)

- [x] Names
- [x] Dates
- [x] Postal addresses (if only requesting/receiving zip codes check Yes to the Zip Code question below instead of checking this box)
- [x] Phone numbers
- [ ] Fax numbers
- [ ] Email addresses
- [ ] Social Security Numbers*
- [x] Medical record numbers
- [ ] Health plan numbers
- [ ] Account numbers

- [ ] License or certificate numbers
- [ ] Vehicle ID numbers
- [ ] Device identifiers or serial numbers
- [ ] Web URLs
- [ ] IP address numbers
- [ ] Biometric identifiers
- [x] Facial photos or other identifiable images
- [ ] Any other unique identifier
- [ ] None

* Could study records include *ANY* photos or images (even 'unidentifiable' ones): **(REQUIRED)**

○ Yes ◉ No

15.11 * ZIP CODES: Some research data sets include zip codes. Will you be receiving data with zip codes as the only portion of an address: (REQUIRED)

Checking 'Yes' here means that you will not be requesting access to any other data element of a patient's address. If you are requesting other parts of an address such as street names and address numbers, check 'No' here and check the box for 'Postal addresses' in the list of 18 PHI Identifiers in the previous question.

◉ Yes ○ No

15.12 * PATIENT RECORDS: Will health information or other clinical data be accessed from UCSF Health, Benioff Children's Hospital Oakland, or Zuckerberg San Francisco General (ZSFG): (REQUIRED)

◉ Yes ○ No

15.13 * CLINICAL DATA - GENERAL DESCRIPTION: Provide a general description of the types of clinical data that you are requesting access to: (REQUIRED)

Participants will be asked and consented to provide their medical record number so that we may link relevant clinical data to the current study. Study staff will either request a report from the EHR system, or will perform a cursory chart review at study points T1, T2, T3, and T4 to record the participant's current medication list and medical problems, including those related to mental health, referrals and utilization data. We will also collect information regarding acute health events and associated antibiotic use that have occurred at the time of visit and the preceding 12 months, and up to 12 months following enrollment. Acute health events of interest include: upper respiratory infection, otitis media, conjunctivitis, and urinary tract infections.

15.14 * CHART/CLINIC NOTES AND OTHER FREE TEXT FIELDS: Will the medical record data include any information extracted from free text fields: (REQUIRED)

○ Yes ◉ No

15.15 * HIPAA - PERMISSION TO ACCESS SENSITIVE DATA: Does the research require access to any of the following types of health information from the medical record: (check all that apply) (REQUIRED)

- [ ] Drug or alcohol abuse, diagnosis or treatment
- [ ] HIV/AIDS testing information
- [ ] Genetic testing information
- [x] Mental health diagnosis or treatment

☐ None of the above

Important note: Ensure that participants initial the corresponding line(s) in Section C of the HIPAA authorization form during the consent process.

15.16 * ACCESS TO OTHER SENSITIVE OR PROTECTED DATA: Are you requesting access to any sensitive health data not protected under HIPAA (any other health history that patients would expect to be kept private such as records relating to treatment for obesity, STDs, compulsive behaviors, embarrassing health conditions, sexual orientation and practices, etc.): (REQUIRED)

○ Yes ◉ No

15.18 * IDENTIFIABILITY OF FINAL DATA SET: (REQUIRED)

Which type of data set are you requesting IRB approval for:

A de-identified data set does not include ANY of the 18 HIPAA identifiers in the list above or any free text fields.

A limited data set (LDS) is described as health information that excludes direct identifiers but that may include:

- City
- State
- ZIP Code
- Elements of date (including dates such as admission, discharge, service, month and year)
- Other numbers, characteristics, or codes not listed as direct identifiers, including ages in years, months or days or hours

Identifiable data sets include direct identifiers and/or information from free text fields.

Review the **HIPAA FAQs on the IRB website** for more details about identifiability of data sets.

◉ De-identified data set
○ Limited data set
○ Identifiable data set without direct identifiers (includes free text fields)
○ Identifiable data set with direct identifiers (may or may not also include free text fields)

15.19 * DATA COLLECTION AND STORAGE: (check all that apply): (REQUIRED)

Collection methods:

☐ Electronic case report form systems (eCRFs), such as OnCore or sponsor-provided clinical trial management portal
☑ UCSF ITS approved Web-based online survey tools: Qualtrics or RedCap
☑ Other web-based online surveys or computer-assisted interview tool
☐ Mobile applications (mobile or tablet-based)
☐ Text Messaging
☐ Wearable devices
☑ Audio/video recordings
☐ Photographs
☑ Paper-based (surveys, logs, diaries, etc.)
☐ Other:

* What online survey or computer assisted interview tool will you use: **(REQUIRED)**

- [ ] Qualtrics (Recommended)
- [x] RedCAP (Recommended)
- [ ] Survey Monkey (NOT recommended and may require UCSF ITS Security review)
- [x] Other

* What's the name of the survey tool and who is it owned by: **(REQUIRED)**

Data collection for the MEFS, BRIEF, BSI-18, CBCL and DECA measures will be administered via secure web-based electronic systems operated by the owners of each measure (Reflection Sciences; PARinc, Pearson Q-Global; Achenbach and Devereaux, respectively). Data will be coded with ID number rather than by name. For measures requiring birthdate in order to correctly apply an age-graded measure, we will round the birthdate to the first of the individual's closest birth month (i.e., 12/11/16 would be entered as 12/01/16).

If the survey tool is not provided by the study sponsor, and the survey tool stores data on a server, vendor, cloud, or 3rd party, contact datasecurity@ucsf.edu to determine if you need to complete a security assessment.

* Data will be collected/stored in systems owned by (check all that apply): **(REQUIRED)**

- [ ] Study sponsor
- [x] UCSF data center (including OnCore, RedCap, Qualtrics, and MyResearch)
- [ ] UCSF encrypted server, workstation, or laptop residing outside of UCSF data center
- [ ] Personal devices, such as laptops or tablets that are not owned or managed by UCSF
- [ ] SF VAMC
- [ ] Zuckerberg San Francisco General Hospital
- [x] Benioff Children's Hospital Oakland
- [ ] Langley Porter Psychiatric Institution
- [ ] Other UCSF affiliate clinic or location (specify below)
- [ ] Cloud vendor such as Amazon Web Services (AWS), Salesforce, etc. (specify below)
- [ ] Other academic institution
- [ ] 3rd party vendor (business entity)
- [ ] Other (explain below)

15.20 * ADDITION OF RECORDS TO A REGISTRY: Will patient records reviewed under this approval be added to a research database, repository, or registry (either already existing or established under this protocol): (REQUIRED)

(●) Yes ( ) No

This activity generally requires patient consent and HIPAA Authorization. A Waiver of Consent/Authorization may be granted for patients who are deceased or lost to follow up, but ongoing patients should be consented at their next clinic visit prior to accessing their health records or they may provide consent and HIPAA authorization for research use of their health information by mail or through a certified E-Signature system such as DocuSign. You may be asked to revise your consent plans.

15.21 * DATA SHARING: During the lifecycle of data collection, transmission, and storage, will identifiable information be shared with or be accessible to anyone outside of UCSF: (REQUIRED)

( ) Yes (●) No

16.0 Financial Considerations

16.1 * PAYMENT: Will subjects be paid for participation, reimbursed for time or expenses, or receive any other kind of compensation: (REQUIRED)

(•) Yes ( ) No

16.2 PAYMENT METHODS: Subjects payment or compensation method (check all that apply):

Payments will be (check all that apply):

- [ ] Cash
- [ ] Check
- [ ] Gift card
- [x] Debit card
- [ ] UCSF Research Subject Payment Card
- [ ] Reimbursement for parking and other expenses
- [ ] Other:

16.3 PAYMENT SCHEDULE: Describe the schedule and amounts of payments, including the total subjects can receive for completing the study:

- **If there are multiple visits over time, explain how payments will be prorated for partial completion**
- **If deviating from recommendations in Subject Payment Guidelines, include specific justification below**

Each participating dyad will be compensated for their time and effort with a total of $300. They will receive $100 for participation in the baseline data collection session (T1), $50 for participation in each of the T2 and T3 sessions, and $100 for participation in the 12-month follow-up data collection session (T4). Compensation will come in form of a debit card.

16.4 COSTS TO SUBJECTS: Will subjects or their insurance be charged for any study activities:

( ) Yes (•) No

17.0 Other Approvals and Registrations

17.4 OTHER APPROVALS: Indicate if this study involves other regulated materials and requires approval and/or authorization from the following regulatory committees:

- [ ] Institutional Biological Safety Committee (IBC)

Specify BUA #:

- [ ] Institutional Animal Care and Use Committee (IACUC)

Specify IACUC #:

- [ ] Controlled Substances

18.0 Qualifications of Key Study Personnel and Affiliated Personnel

NEW: January 2019 - Affiliated personnel who do not need access to iRIS no longer need to get a UCSF ID. Instead, add them below in the Affiliated Personnel table below.

18.1 **Qualifications of Key Study Personnel:**

Instructions:

For UCSF Key Study Personnel (KSP)* listed in **Section 3.0,** select the KSP from the drop down list and add a description of their study responsibilities, qualifications and training. In study responsibilities, identify every individual who will be involved in the consent process. Under qualifications, please include:

- Academic Title
- Institutional Affiliation (UCSF, SFGH, VAMC, etc.)
- Department
- Certifications

NOTE: This information is required and your application will be considered incomplete without it. If this study involves invasive or risky procedures, or procedures requiring special training or certification, please identify who will be conducting these procedures and provide details about their qualifications and training. Click the orange question mark for more information and examples.

Training Requirements:
The IRB requires that all Key Study Personnel complete Human Subjects Protection Training through **CITI** prior to approval of a new study, or a modification in which KSP are being added. More information on the CITI training requirement can be found on our **website**.

* **Definition of Key Study Personnel and CITI Training Requirements (Nov, 2015):** UCSF Key Study Personnel include the Principal Investigator, other investigators and research personnel who are directly involved in conducting research with study participants or who are directly involved in using study participants' identifiable private information during the course of the research. Key Personnel also include faculty mentors/advisors who provide direct oversight to Postdoctoral Fellows, Residents and Clinical Fellows serving as PI on the IRB application.

KSP Name	Description of Study Responsibilities - Briefly describe what will each person be doing on the study. If there are procedures requiring special expertise or certification, identify who will be carrying these out. Also identify who will be obtaining informed consent.	Qualifications, Licensure, and Training
Long, Dayna MD	Principal Investigator: Oversees all aspects of the proposed study including design, data collection, analysis, recruitment, informed consent and reporting of results.	Dr. Long is an MD at UCSF Benioff Children's Hospital Oakland. She has over 15 years of experience in conducting studies. She is the

			PI in several NIH asthma and social determinants of health studies
	Benson, Mindy S	Co-Principal Investigator: Will oversee management of the study as delegated by the PI. Will assist with recruitment and informed consent.	Pediatric Nurse Practicioner at UCSF Benioff Children's Hospital Oakland for more than 20 years. She also has more than 26 years in clinical research ranging in HIV, asthma and social determinants of health studies.
	Thakur, Neeta MD	Co-Principal Investigator: Will oversee management of the study as delegated by the PI. Will be responsible in collection and storage of biomarkers.	Dr. Thakur is a pulmonary and critical care physician at UCSF. She has over 10 years of experience in conducting studies. She is the PI in several asthma and social determinants of health studies.
	Jeung, Joan J	Co-Principal Investigator: Will oversee management of the study as delegated by the PI. Will assist with recruitment and informed consent.	Dr. Joan Jeung is a pediatrician with special training in developmental and behavioral pediatric medicine. Her research interests focus on ways to address the unmet behavioral, developmental and mental health needs of children and teens, especially within primary care settings.
	Mok, Roberto	Nurse/Study coordinator: Will do recruitment and consenting as well as performing all study procedures.	Study coordinator/LVN for over 15 years at UCSF Benioff Children's Hospital Oakland. CITI certification.
	Harris, Cherri L	Nurse/Study coordinator: Will do recruitment and consenting as well as performing all study procedures.	Study coordinator/LVN for over 15 years at UCSF Benioff Children's Hospital Oakland. CITI certification.
	Yee, Morgan M	Data Manager/Analyst: Will be responsible for the data integrity of the study	Data Analyst, completing cross-sectional and longitudinal analyses
	De La Rosa, Rosemarie	She will work with Dr. Thakur to geocode addresses of PEARL participants.	Dr. Delarosa is a post-doctoral fellow with training in environmental health sciences and toxicology.

18.2 Affiliated Personnel:

Instructions:

This section is for personnel who are not listed in **Section 3.0: Grant Key Personnel Access to the Study** because their names were not found in the User Directory when both the iRIS Database and MyAccess directories were searched. Add any study personnel who fit ALL of the following criteria in the table below:

- They meet the definition of Key Study Personnel (see above), **and**
- They are associated with a UCSF-affiliated institution (e.g., VAMC, Gladstone, Institute on Aging, Vitalant, NCIRE, SFDPH, or ZSFG), **and**
- They do not have a UCSF ID, **and**
- They do not need access to the study application and other study materials in iRIS.

Note: Attach a **CITI Certificate** for all persons listed below in the **Other Study Documents** section of the **Initial Review Submission Packet Form** after completing the **Study Application**.

Click the orange question mark icon to the right for more information on who to include and who not to include in this section.

Do not list personnel from outside sites/non-UCSF-affiliated institutions. Contacts for those sites (i.e. other institution, community-based site, foreign country, or Sovereign Native American nation) should be listed in the **Outside Sites** section of the application.

If there are no personnel on your study that meet the above criteria, leave this section blank.

Name	Institution	Telephone	E-mail	Role
No External Personnel has been added to this IRB Study				

Please describe the study responsibilities and qualifications of each affiliated person listed above:

19.0 End of Study Application

End of Study Application Form

To continue working on the Study Application:
Click on the section you need to edit in the left-hand menu. Remember to save through the entire Study Application after making changes.

If you are done working on the Study Application:
Important: Before proceeding, please go back to Section 4.0 Initial Screening Questions and **Save and Continue** through the form to make sure all the relevant sections and questions have been included. If you've changed any answers since you started, the branching may have changed. Your application will be incomplete and it will have to be returned for corrections.

Once you are sure the form is complete, click **Save and Continue**. If this is a new study, you will automatically enter the **Initial Review Submission Packet Form**, where you can attach **consent forms** or other **study documents**. Review the **Initial Review Submission Checklist** for a list of required attachments.

Answer all questions and attach all required documents to speed up your approval.

The UCSF IRB welcomes feedback about the IRB Study Application Form. Please click the link to answer a **survey** about the application form.
