# Supplementary material for: Screening for adverse childhood experiences in pediatrics: A randomized trial of aggregate-level versus item-level response screening formats
Source: PLoS One. 2022 Dec 15;17(12):e0273491. doi: 10.1371/journal.pone.0273491 (PMC9754205; doi:10.1371/journal.pone.0273491)
Supplement: S4 Appendix — (DOCX) [file pone.0273491.s007.docx]

**PEARLS Amendments 2016-2021**

**Summary of amendments and changes to the protocol**

1. **Personnel**
2. **Protocol**
3. Blood draw moved at T2
4. Added Curriculum for Resilience Clinic Intervention
5. Follow up window extended to 3 months
6. Additional ways to contact families: text and appointment cards added to phone calls.
7. Added measures: PANAS and cognitive interviews
8. Changes to language in the data collection forms

**Personnel:**

**Amendment** 01/11/2021

Dr. Joan Jeung is the new Director for the resilience clinic intervention.

**Amendment** 03/01/2019

Removing both Laura Frame, Rachel Gilgoff and Adam Davis as co-investigators and Ana Hernandez as study coordinator as they're no longer involved with the study**.**

**Protocol:**

**Amendment 02/04/2020**

Hosting a family reunion on Saturday, 6/20/2020 for all PEARLS participants and family as a way to thank them for their participation and to share study updates. There will be various activities for both children and adults and food will be provided (see attached agenda).

Included is the letter "Letter to Family" that will be sent to their address of record.

As for the included power point presentation, this is not the final presentation but information from this file will be used for the presentation.

**Amendment 04/12/2019**

This is a "thank you" letter for participants who complete the exit questionnaire (amendment approval 3/19/2019) who would want their payment of $25 sent to them**.**

**Amendment 03/19/2019**

The study team has decided to add an exit interview to be completed by participants after they have completed their T4 visit. The exit interview will ask 4 questions in total about caretakers' experience with the PEARLS study including what they learned, what were the barriers to participation and about what we could have done differently. The interviewer will be blinded as to what intervention the participate was randomized to and any other study data about the participant.

The study team hopes that the information gained by the exit interview will help us to improve our research in the future.

**Amendment 08/24/2017**

1. We would like to start giving caregivers who have been randomized to the Resiliency Clinic an appointment card. (Card attached.)

2. We would like to add texting and phone calls as a means of communication with study participants. This will be added to the consent form. (Updated version attached).

We had planned to communicate with participants on a regular basis month via messages in MyChart. However, some families don’t want to use MyChart. We would like to change the protocol slightly by offering to send messages between research visits via MyChart, GroupWise text, or phone call.

Messaging may be used for the following reasons:

Reminder messages may be sent with the time and date of future study-related visits.

Generic messages may be sent to re-enforce learning done during the intervention arms of the study (care coordination sessions or resiliency clinic visits).

Messages will not contain any personal information.

Study staff may call families to remind them of upcoming visits. These phone calls will also follow a generic messaging format.

Confidentiality/Privacy/Safety/Consent:

We are submitting a revised study consent to offer families the option to receive messages by MyChart, text and/or phone. Families who do not consent to any form of communication will still be able to participate in all other aspects of the study including Care Coordination and Resiliency Clinic.

Families that have already been consented will be asked to re-consent so that they may have the option to receive messages by MyChart, text and/or phone call.

3. Adding new cognitive interview questions.

We have asked over 70 families the existing cognitive interview questions (which evaluated caregivers’ perceptions and attitudes towards identified versus de-identified ACE screening). We have obtained enough data on those questions, and would like to start asking new questions. The new questions focus on caregivers’ perceptions and attitudes towards anticipatory guidance on ACEs.

**Amendment 02/13/2017**

1. We made several minor changes to the data collect forms in terms of language. All these changes to the data collection forms have track changes and are uploaded for review. For instance, we streamlined the health assessment form and simplified the chart review form.
2. We moved around the timing of the data collection for patient ease. We minimized the data being collected at T1 and added to T2. We moved the adult surveys about adverse childhood experiences to T4.
3. We are adding stool sample collection to T2. W uploaded the protocol for sample collection and added language to the consent.
Stool collection for microbiome.

PEARLS aims to elucidate the pathway from chronic stress to poor health outcomes. We know that chronic stress can affect a variety of biological processes, including the neuro-endocrine and inflammatory systems. There is a growing body of evidence that chronic stress and poverty can change an individual’s microbiome, the bacteria, viruses and fungi that inhabit the body on a regular basis and contribute to its function. Currently, we are measuring the nasal and oral microbiome, with this modification we will extend this measurement to the gut. There is an ever-growing body of literature connecting chronic stress to modifications of the gut microbiome (gut-brain axis), and it has been hypothesized that these changes are important for health, including atopic diseases, such as asthma and eczema, and for metabolic diseases. To measure the gut microbiome, we will ask participants to collect a stool samples with the kits provided (see participant instructions for details). These samples will be brought back, in the appropriate container, at visit T2. Participants that do not bring back their samples will be provided with mailing instructions, including self-addressed and stamped samples. Samples brought to visit 2 will be sent to the Adversity BioCore (ABC) Bank at UCSF along with the other biospecimens collected that day. For participants mailing in their samples, these will be mailed directly to the ABC Bank at UCSF.

4. We finished the curriculum for the resilience clinic intervention for 3-5 year olds and 6 to 11 year olds and have uploaded the curriculum for patients randomized to this arm of the  study. We are still working on the 0-2 year old curriculum and will upload it in the next revision.

5. We added language for the provider anticipatory guidance that is to inform the discussion that the pediatricians will have with families at T1 after the families are enrolled, consented and have completed the survey about their child's adverse childhood experiences. The anticipatory guidance scripts give the providers tips about how to talk to families about trauma and resilience. Pediatricians will pass out the Connected Families Connected Kids handout.

6. We had changes in staffing. Ana Hernandez is no longer a co-I and we added Roberto Mok as a study coordinator.

7. Appointment Reminder forms for each visit will be given to participant.  These forms are under filenames:  Visit 2 Appointment Reminder.doc; Visit 3 Appointment Reminder.doc; Visit 4 Appointment Reminder.doc.

8. Added two forms for Biospecimen (blood, nasal, mouth, and stool) to document the collection of the specimen and to send off to the lab.  These forms are for Study Personnel to complete.

9. For families randomized to the Care Coordination arm of the RCT, if they screen for concerns about the child's mental health, the child will be referred by study staff to existing internal mental health services. A form (Dear Primary Care Provider_1.doc) was created to document the referral and to communicate with the primary care provider that the referral was ordered by study staff. 

10. Updated recruitment flyer to reflect payment for each visit.  My apologies for not able to do track changes as this flyer was originally done on MS Publisher.

**Amendment 03/02/2017**

1.  We would like to start giving caregivers who have been randomized to the Resiliency Clinic an appointment card.

2.  We would like to add texting and phone calls as a means of communication with study participants. This will be added to the consent form. (Updated version attached).

We had planned to communicate with participants on a regular basis month via messages in MyChart.  However, some families don’t want to use MyChart. We would like to change the protocol slightly by offering to send messages between research visits via MyChart, GroupWise text, or phone call.

Messaging may be used for the following reasons:

- Reminder messages may be sent with the time and date of future study-related visits.
- Generic messages may be sent to re-enforce learning done during the intervention arms of the study (care coordination sessions or resiliency clinic visits).
- Messages will not contain any personal information.
- Study staff may call families to remind them of upcoming visits. These phone calls will also follow a generic messaging format.

Confidentiality/Privacy/Safety/Consent:

We are submitting a revised study consent to offer families the option to receive messages by MyChart, text and/or phone. Families who do not consent to any form of communication will still be able to participate in all other aspects of the study including Care Coordination and Resiliency Clinic.

Families that have already been consented will be asked to re-consent so that they may have the option to receive messages by MyChart, text and/or phone call.  

3.  Adding new cognitive interview questions.

We have asked over 70 families the existing cognitive interview questions (which evaluated caregivers’ perceptions and attitudes towards identified versus de-identified ACE screening). We have obtained enough data on those questions, and would like to start asking new questions. The new questions focus on caregivers’ perceptions and attitudes towards anticipatory guidance on ACEs

**Amendment 03/02/2017**

We refined the language for the anticipatory guidance worksheet that will be given to providers.  This worksheet will help prepare providers to speak with families about the ACE screen results during their clinical visit.
